# Choice across 10 pharmacologic combination strategies for type 2 diabetes: a cost-effectiveness analysis

**DOI:** 10.1186/s12916-020-01837-x

**Published:** 2020-12-03

**Authors:** Shuyan Gu, Lizheng Shi, Hui Shao, Xiaoyong Wang, Xiaoqian Hu, Yuxuan Gu, Hengjin Dong

**Affiliations:** 1 Center for Health Policy and Management Studies, School of Government, Nanjing University, Nanjing, Jiangsu China; 2 Center for Health Policy Studies, School of Public Health, School of Medicine, Zhejiang University, Hangzhou, Zhejiang China; 3 Department of Global Health Management and Policy, School of Public Health and Tropical Medicine, Tulane University, New Orleans, LA USA; 4 Department of Pharmaceutical Outcomes and Policy, College of Pharmacy, University of Florida, Gainesville, FL USA; 5 Health Insurance Office, Shandong Provincial Hospital Affiliated to Shandong First Medical University, Jinan, Shandong China; 6 Current address: College of Politics and Public Administration, Qingdao University, Qingdao, Shandong China

**Keywords:** Type 2 diabetes, Cost-effectiveness, Metformin, Sulfonylurea, Thiazolidinedione, α-Glucosidase inhibitor, Glinide, DPP-4 inhibitor, Insulin, GLP-1 receptor agonist

## Abstract

**Background:**

Clinical guidelines recommend a stepped-escalation treatment strategy for type 2 diabetes (T2DM). Across multiple treatment strategies varying in efficacy and costs, no clinical or economic studies directly compared them. This study aims to estimate and compare the cost-effectiveness of 10 commonly used pharmacologic combination strategies for T2DM.

**Methods:**

Based on Chinese guideline and practice, 10 three-stepwise add-on strategies were identified, which start with metformin, then switch to metformin plus one oral drug (i.e., sulfonylurea, thiazolidinedione, α-glucosidase inhibitor, glinide, or DPP-4 inhibitor) as second line, and finally switch to metformin plus one injection (i.e., insulin or GLP-1 receptor agonist) as third line. A cohort of 10,000 Chinese patients with newly diagnosed T2DM was established. From a healthcare system perspective, the Cardiff model was used to estimate the cost-effectiveness of the strategies, with clinical data sourced from a systematic review and indirect treatment comparison of 324 trials, costs from claims data of 1164 T2DM patients, and utilities from an EQ-5D study. Outcome measures include costs, quality-adjusted life-years (QALYs), incremental cost-effectiveness ratios (ICERs), and net monetary benefits (NMBs).

**Results:**

Over 40-year simulation, the costs accumulated for a patient ranged from $7661 with strategy 1 to $14,273 with strategy 10, while the QALY gains ranged from 13.965 with strategy 1 to 14.117 with strategy 8. Strategy 7 was dominant over seven strategies (strategies 2~6, 9~10) with higher QALYs but lower costs. Additionally, at a willingness-to-pay threshold of $30,787/QALY (i.e., 3 times GDP/capita for China), strategy 7 was cost-effective compared with strategy 1 (ICER of strategy 7 vs. 1, $3371/QALY) and strategy 8 (ICER of strategy 8 vs. 7, $132,790/QALY). Ranking the strategies by ICERs and NMBs, strategy 7 provided the best value for money when compared to all other strategies, followed by strategies 5, 9, 8, 1, 3, 6, 10, 2, and 4. Scenario analyses showed that patients insist on pharmacologic treatments increased their QALYs (0.456~0.653) at an acceptable range of cost increase (ICERs, $1450/QALY~$12,360/QALY) or even at cost saving compared with those not receive treatments.

**Conclusions:**

This study provides evidence-based references for diabetes management. Our findings can be used to design the essential drug formulary, infer clinical practice, and help the decision-maker design reimbursement policy.

**Supplementary information:**

The online version contains supplementary material available at 10.1186/s12916-020-01837-x.

## Background

Type 2 diabetes (T2DM) as a chronic progressive disease imposes a substantial disease burden on patients and the healthcare system [1]. China has the world’s largest diabetes epidemic, with 116.4 million adults with diabetes in 2019 [1]. Total diabetes-related health expenditure reached $109.0 billion, ranking second to the USA globally [1]. How to properly reduce medical expenses along with improving patients’ health has become a common concern of patients, governments, and the society. Chinese clinical guidelines advocate a stepwise failure-driven treatment strategy for blood glucose lowering that leads to the sequential addition of therapies [2]. Metformin is the preferred initial therapy, which is recommended to be maintained throughout the treatment [2]. Once metformin fails to achieve glucose target, multiple oral glucose-lowering drugs were available as add-on therapy to metformin, such as sulfonylurea, thiazolidinedione, α-glucosidase inhibitor, glinide, and dipeptidyl peptidase 4 (DPP-4) inhibitor. If oral dual therapy fails to effectively control glucose, injections like insulin or glucagon-like peptide 1 (GLP-1) receptor agonist can be added onto metformin [2].

A number of choices for add-on glucose-lowering treatment have increased uncertainty regarding the optimal treatment path, while increasing the complexity of treatment choice in clinical practice. A nationwide survey of treatment pattern of oral drugs users in China reported that metformin was used by 53.7% of T2DM patients, followed by sulfonylureas (42.7%), α-glucosidase inhibitors (35.9%), glinides (27.5%), thiazolidinediones (17.2%), and DPP-4 inhibitors (0.8%) [3]. Dual combination therapy was more commonly used than monotherapy, of which metformin plus sulfonylureas was the most common (27.7%), followed by metformin plus glinides [3]. Medication adherence was unsatisfactory in China with 31.9% of the patients altering treatment regimens within 1 year. The main reasons cited for treatment alterations were insufficient efficacy (21.9%), adverse reaction (4.3%), and treatment budget (1.7%) [3]. Poor adherence would in turn impede treatment effect, further to increase the risks of diabetes-related complications and healthcare costs [4–6]. Lack of a comprehensive understanding of the efficacy, safety, and costs of the existing treatments is a potential reason for the failure to choose appropriate treatment.

Informed decisions regarding optimal prescribing and reimbursement of glucose-lowering drugs by healthcare payers require information about efficacy, costs, and cost-effectiveness. It is important to study the costs and benefits of the existing glucose-lowering treatments, to help clinicians and decision-makers choose the most cost-effective treatments. However, clinical evidence of head-to-head studies that directly compared all the glucose-lowering treatments against each other can hardly be fully obtained [7]. Besides, T2DM treatment is a lifetime task that often requires a sequential use of drug monotherapy and a combination of drugs to ensure intensive glycemic control. Thus, it is of value to conduct economic evaluations based on a lifelong treatment strategy, to evaluate the therapies used by a patient from the start of medication to the end of life. This could help us comprehensively understand the impacts of different treatments on disease burden of patients. However, economic evaluations of glucose-lowering treatments in China were mostly short-term cost-effectiveness analyses conducted alongside the clinical trials, and several long-term modeling studies focused on several individual drugs as monotherapy or add-on therapy at a specific treatment stage. There was a lack of clinical or economic studies that directly compared all the existing lifelong treatment strategies against each other within one study.

Therefore, this study aimed to estimate and compare the lifetime cost-effectiveness of 10 commonly used pharmacologic combination strategies for patients with newly diagnosed T2DM in China, so as to provide unified hierarchies of evidence for the holistic management of T2DM.

## Methods

From a healthcare system perspective, the cost-effectiveness of the pharmacologic combination strategies was evaluated using an existing stochastic simulation model, the Cardiff diabetes model.

### Model overview

**Fig. 1:**
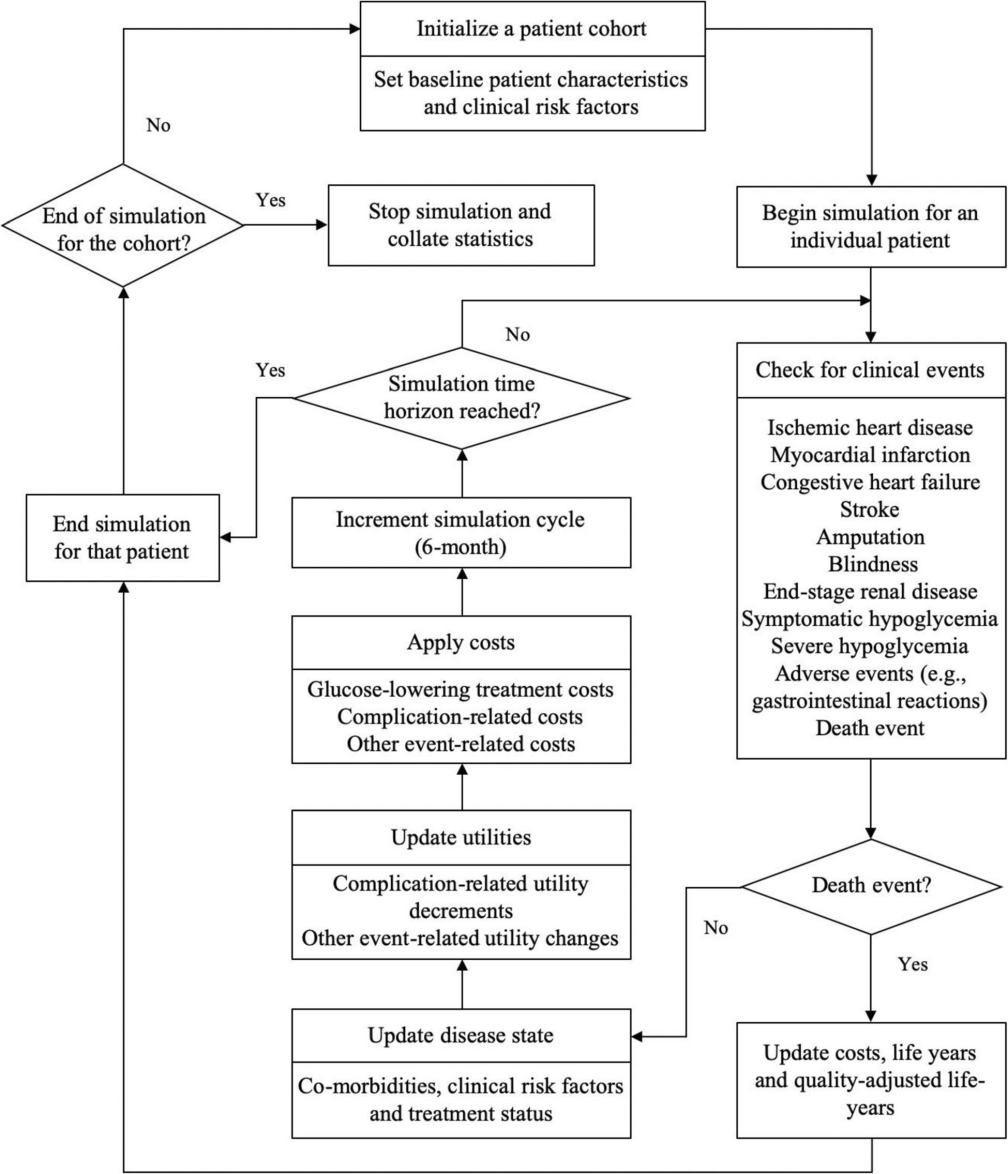
Logical flow of patient simulation process in the Cardiff diabetes model

The Cardiff diabetes model is a patient-level fixed-time increment, Monte Carlo micro-simulation model, which is designed to evaluate the cost-effectiveness of comparable treatment strategies in diabetes [8–14]. Each treatment strategy is comprised of three therapy lines. The model simulates multiple disease courses and predicts the occurrences of diabetes-related complications and death based on the United Kingdom Prospective Diabetes Studies (UKPDS) 68 or 82 risk equations, combined with patient characteristics, clinical risk factors, and treatment-induced changes in the clinical risk factors [13, 14]. The risk factors include glycosylated hemoglobin Alc (HbA1c), cholesterol, blood pressure, and weight. The natural progressions of HbA1c, cholesterol, and blood pressure are modeled via the implementation of UKPDS 68 risk equations, and that of weight is modeled linearly based on a weight gain of 0.1 kg per year by default. The logical flow of the model is shown in Fig. 1. Diabetes-related complications include macrovascular events (i.e., ischemic heart disease, myocardial infarction, congestive heart failure, and stroke) and microvascular events (i.e., blindness, end-stage renal disease, and amputation). Model inputs include patient profiles of the initial cohort, treatment effects and pharmacy costs of glucose-lowering treatments, and costs and utility changes associated with diabetes-related events.

The UKPDS 68 risk equations were used in the base-case analysis, and the UKPDS 82 risk equations were tested in the sensitivity analysis. A lifetime horizon of 40 years was simulated with a discount rate of 3% for costs and benefits [15]. Outcome measures include costs, quality-adjusted life-years (QALYs), incremental cost-effectiveness ratios (ICERs), and net monetary benefits (NMBs). The ICER is calculated as incremental costs divided by incremental QALYs of two treatment strategies. As this study had multiple treatment strategies, they were ranked as follows: (i) rank the strategies in order of costs and compare each strategy to its adjacent and lower-cost alternative; (ii) rule out strongly dominated alternatives (i.e., with higher cost but lower QALY compared with its adjacent); (iii) calculate ICERs based on the comparisons of moving to increasingly costly and increasingly effective alternatives; (iv) if the ICER associated with moving to more costly alternative falls, then the lower-cost alternative used to calculate the ICER is extendedly dominated and is ruled out; (v) recalculate ICERs based on comparisons of moving to increasingly costly but increasingly effective alternatives that are neither strongly nor extendedly dominated; and (vi) compare the ICER with willingness-to-pay/QALY—if it is within willingness-to-pay/QALY, the higher-cost alternative used to calculate the ICER is cost-effective; otherwise, the lower-cost one is cost-effective [16]. The NMB is calculated as QALYs times willingness-to-pay/QALY, minus costs of each strategy. It is a rearrangement of the cost-effectiveness decision rule that summarizes the difference between the economic value of health benefits and the costs [16–18]. The larger the NMB value, the more cost-effective the strategy is [16–18]. This study used a willingness-to-pay threshold of ¥212,676 ($30,787)/QALY (3 times gross domestic product per capita of China, 2019) [19] according to the Chinese guideline [20].

### Patient cohort and patient profiles

An initial patient cohort of 10,000 Chinese adult patients with newly diagnosed T2DM was established. Patient profiles of the cohort were sourced from a nationwide prospective cohort study [21, 22]. The study recruited 5770 Chinese patients with newly diagnosed T2DM from 79 hospitals across six geographic regions of China, which had comprehensive baseline patient profiles. As it only reported body mass index (BMI) and weight of the patients, height was calculated using BMI and weight (Table 1) [21, 22].

**Table 1:** Model inputs: patient profiles, treatment effects, costs, and utility changes

**Patient profiles of the initial cohort**	**Value**		**Source**								
**Patient characteristics**	**Mean**	**SE**									
Age, year	55.7	0.17	[21, 22]								
Female proportion, %	45.8	0.70									
Duration of diabetes, year	0.50	–									
Height, meter^**a**^	1.65	0.02									
Smokers proportion, %	22	0.50									
**Modifiable risk factors**	**Mean**	**SE**									
HbA1c, %	8.40	0.03	[21, 22]								
TC, mg/dl^**b**^	193.05	0.66									
HDL-C, mg/dl^**b**^	46.33	0.20									
LDL-C, mg/dl^**b**^	111.97	0.51									
SBP, mmHg	129	0.18									
Weight, kg^**c**^	68.20	2.42									
**Treatment effects of first therapy**^**d**^	**Metformin**		**Source**								
	**Mean**	**SE**									
HbA1c change, %	− 1.57	0.22	Meta-analysis and indirect treatment comparison								
TC change, mg/dl^**b**^	− 30.50	6.30									
HDL-C change, mg/dl^**b**^	10.04	2.36									
SBP change, mmHg	− 0.36	2.26									
Weight change, kg^**e**^	− 7.51	1.09									
Symptomatic hypoglycemia, %^**f**^	1.81	1.04									
Severe hypoglycemia, %^**f**^	0.04	0.02									
Gastrointestinal reactions, %	2.15	1.06									
**Treatment effects of second therapy**^**d**^	**Metformin + sulfonylurea**		**Metformin + thiazolidinedione**		**Metformin + α-glucosidase inhibitor**		**Metformin + glinide**		**Metformin + DPP-4 inhibitor**		**Source**
	**Mean**	**SE**	**Mean**	**SE**	**Mean**	**SE**	**Mean**	**SE**	**Mean**	**SE**	
HbA1c change, %	− 2.61	0.18	− 2.40	0.22	− 2.41	0.28	− 2.64	0.25	− 2.52	0.22	Meta-analysis and indirect treatment comparison
TC change, mg/dl^**b**^	− 6.56	2.36	− 8.88	4.81	− 31.66	12.06	− 2.70	5.10	− 24.32	5.46	
HDL-C change, mg/dl^**b**^	− 1.16	3.25	5.41	5.73	11.58	5.52	− 4.63	6.50	2.70	4.24	
SBP change, mmHg	− 3.80	1.17	− 4.64	3.06	− 3.68	3.02	–	–	− 6.22	2.52	
Weight change, kg^**e**^	− 0.95	1.21	− 1.42	1.69	− 2.70	1.60	− 8.06	1.43	− 4.14	1.36	
Symptomatic hypoglycemia, %^**f**^	4.46	0.57	1.52	0.98	0	1.37	0	1.19	0.54	1.06	
Severe hypoglycemia, %^**f**^	0.09	0.01	0.03	0.02	0	0.03	0	0.02	0.01	0.02	
Gastrointestinal reactions, %	4.62	0.59	7.62	1.41	9.62	1.55	7.62	1.74	6.62	1.27	
**Treatment effects of third therapy**^**d**^	**Metformin + insulin**		**Metformin + GLP-1 receptor agonist**		**Source**						
	**Mean**	**SE**	**Mean**	**SE**							
HbA1c change, %	− 2.51	0.29	− 2.89	0.20	Meta-analysis and indirect treatment comparison						
TC change, mg/dl^**b**^	− 69.50	12.15	− 55.60	7.88							
HDL-C change, mg/dl^**b**^	–	–	0	5.71							
SBP change, mmHg	–	–	− 7.38	1.62							
Weight change, kg^**e**^	− 4.22	2.69	− 7.54	0.92							
Symptomatic hypoglycemia, %^**f**^	3.47	0.58	3.47	0.58							
Severe hypoglycemia, %^**f**^	0.07	0.01	0.07	0.01							
Gastrointestinal reactions, %	11.78	2.22	6.78	0.98							
**Costs of glucose-lowering treatments**	**Cost, ¥ ($)**^**g,i**^		**Individual drug**		**Cost, ¥ ($)**^**g,h**^	**Source**					
Metformin	679.05 (98.30)		Metformin		679.05 (98.30)	[23]					
Sulfonylurea	632.74 (91.60)		Glyburide		2.70 (0.39)						
			Glimepiride		1210.95 (175.30)						
			Gliclazide		414.59 (60.02)						
			Glipizide		902.72 (130.68)						
Thiazolidinedione	1148.06 (166.19)		Rosiglitazone		938.83 (135.90)						
			Pioglitazone		1357.28 (196.48)						
α-Glucosidase inhibitor	1815.60 (262.83)		Acarbose		1830.98 (265.05)						
			Voglibose		1356.07 (196.30)						
			Miglitol		2259.77 (327.12)						
Glinide	1124.13 (162.73)		Repaglinide		1259.80 (182.37)						
			Nateglinide		988.46 (143.09)						
DPP-4 inhibitor	3069.95 (444.41)		Sitagliptin		2814.34 (407.40)						
			Saxagliptin		3017.02 (436.74)						
			Vildagliptin		3192.47 (462.14)						
			Linagliptin		3137.21 (454.14)						
			Alogliptin		3188.72 (461.60)						
GLP-1 receptor agonist	14,578.94 (2110.44)		Exenatide		18,990.33 (2749.03)						
			Liraglutide		10,167.55 (1471.85)						
Insulin cost per kg weight per day	0.137 (0.020)		–		–						
**Costs and utility changes associated with diabetes-related events**	**Fatal costs, ¥ ($)**^**g**^		**Non-fatal costs, ¥ ($)**^**g**^		**Maintenance costs, ¥ ($)**^**g**^		**Source**	**Utility change**			**Source**
	**Mean**	**SE**	**Mean**	**SE**	**Mean**	**SE**		**Mean**	**SE**		
Ischemic heart disease	21,574.18 (3123.07)	6359.88 (920.65)	23,860.32 (3454.01)	1576.61 (228.23)	3293.02 (476.70)	435.07 (62.98)	Claims data	− 0.028	0.005		[24]
Myocardial infarction	39,463.58 (5712.74)	8360.32 (1210.24)	53,131.50 (7691.30)	3774.74 (546.43)	6543.01 (947.16)	–		− 0.028	0.005		
Congestive heart failure	35,521.34 (5142.06)	13,196.13 (1910.27)	32,469.77 (4700.31)	6315.33 (914.21)	3113.24 (450.67)	1797.6 (260.22)		− 0.028	0.005		
Stroke	69,427.06 (10,050.24)	18,792.10 (2720.34)	25,465.96 (3686.44)	2418.01 (350.03)	4274.23 (618.74)	735.06 (106.41)		− 0.101	0.006		
Amputation	–	–	22,281.86 (3225.52)	–	3542.36 (512.79)	–		− 0.118	0.009		
Blindness	–	–	15,846.21 (2293.89)	617.89 (89.45)	5227.01 (756.66)	484.87 (70.19)		− 0.022	0.005		
End-stage renal disease	15,531.40 (2248.32)	3715.52 (537.86)	16,002.73 (2316.55)	554.07 (80.21)	5595.21 (809.96)	543.32 (78.65)		− 0.058	0.006		
Symptomatic hypoglycemia	–		0		–		Assumed	− 0.007	0.002		
Severe hypoglycemia	–		4116.10 (595.85)		–		[25]	− 0.008	0.004		
Gastrointestinal reactions	–		0		–		Assumed	− 0.034	0		[26]
BMI per unit increase	–		–		–			− 0.0061	0.001		[27]
BMI per unit decrease	–		–		–			+ 0.0061	0.001		
**BMI-related costs**	**Costs, ¥ ($)**^**g**^		**BMI**	**Costs, ¥ ($)**^**g**^		**BMI**	**Costs, ¥ ($)**^**g**^		**Source**		
≤ 23	0		29	14,230.2 (2059.96)		35	29,252.9 (4234.64)		[28]		
24	1711.3 (247.73)		30	16,734.0 (2422.41)		36	31,756.7 (4597.09)				
25	4215.1 (610.18)		31	19,237.8 (2784.86)		37	34,260.5 (4959.54)				
26	6718.9 (972.63)		32	21,741.6 (3147.31)		38	36,764.3 (5321.99)				
27	9222.7 (1335.08)		33	24,245.4 (3509.76)		39	39,268.0 (5684.42)				
28	11,726.4 (1697.51)		34	26,749.1 (3872.19)		≥ 40	41,771.8 (6046.87)				

Sulfonylurea includes glyburide, glimepiride, gliclazide, and glipizide. Thiazolidinedione includes rosiglitazone and pioglitazone. α-Glucosidase inhibitor includes acarbose, voglibose, and miglitol. Glinide includes repaglinide and nateglinide. DPP-4 inhibitor includes sitagliptin, saxagliptin, vildagliptin, linagliptin, and alogliptin. Insulin includes various kinds of insulin and insulin analogs. GLP-1 receptor agonist includes exenatide and liraglutide

*BMI* body mass index, *DPP-4* dipeptidyl peptidase 4, *GLP-1* glucagon-like peptide 1, *HbA1c* glycosylated hemoglobin Alc, *HDL-C* high-density lipoprotein-cholesterol, *LDL-C* low-density lipoprotein-cholesterol, *RCT* randomized controlled trial, *SBP* systolic blood pressure, *SE* standard error, *TC* total cholesterol

^a^As only BMI and weight of the patients were reported, height was calculated by: sqrt (weight/BMI)

^b^The unit of cholesterol in the included 324 RCTs was mmol/l, while the Cardiff model requires mg/dl, which was converted by: 1 mg/dl = 0.0259 mmol/l [29]

^c^The “criteria of weight for adults” published by the National Health Commission of China defines weight categories as underweight (BMI < 18.5 kg/m^2^), normal weight (18.5 ≤ BMI < 24 kg/m^2^), overweight (24 ≤ BMI < 28 kg/m^2^), and obese (BMI ≥ 28 kg/m^2^) [30]. As the BMI of patients in our initial cohort was 25 kg/m^2^, they were overweight

^d^The treatment effect of each glucose-lowering treatment was with or without a background of lifestyle interventions

^e^Only BMI change was reported in the 324 RCTs, while the Cardiff model requires weight change; thus, it was calculated by: weight = BMI × height^2^

^f^Hypoglycemia is differentiated as symptomatic and severe ones in the Cardiff model, but most of the 324 RCTs did not clearly differentiate between symptomatic and severe episodes; thus, we estimated that a rate of 2% represented the proportion of severe cases out of all hypoglycemia events [25]

^g^For the costs, data are 2019 Chinese yuan, ¥ (2019 US dollar, $). One US dollar was equal to ¥6.908 in 2019 [31]

^h^Annual pharmacy cost of a drug was calculated as its retail price times its annual dose. The retail price was sourced from government medicine purchase platform, and the drug dose was obtained from the 324 RCTs. One year was counted as 365 days. For individual drugs, only the drugs reported in the 324 RCTs were included

^i^Annual pharmacy costs of each drug class were calculated based on a simple arithmetic average of the costs of individual drugs

### Treatment strategy

**Fig. 2:**
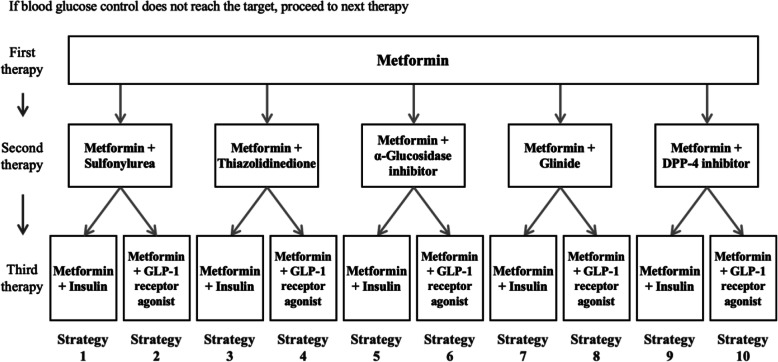
Treatment algorithm for blood glucose control in type 2 diabetes and treatment paths of the 10 pharmacologic combination strategies. Sulfonylurea includes glyburide, glimepiride, gliclazide, glipizide, and gliquidone. Thiazolidinedione includes rosiglitazone and pioglitazone. α-Glucosidase inhibitor includes acarbose, voglibose, and miglitol. Glinide includes repaglinide, nateglinide, and mitiglinide. DPP-4 inhibitor includes sitagliptin, saxagliptin, vildagliptin, linagliptin, and alogliptin. Insulin includes various kinds of insulin and insulin analogs. GLP-1 receptor agonist includes exenatide and liraglutide

A treatment strategy was defined to consist of a three-stepwise escalation treatment path in this study based on the Chinese clinical guideline [2] and the setting of Cardiff diabetes model. Ten pharmacologic combination strategies were identified based on the Chinese clinical guideline and clinical practice [2]. Patients started the simulation by receiving metformin monotherapy (first step). When patients’ glucose control did not reach the target of HbA1c level < 8%, the first therapy escalation occurred: they switched to an oral dual therapy of one of the five classes of oral drugs (i.e., sulfonylurea, thiazolidinedione, α-glucosidase inhibitor, glinide, or DPP-4 inhibitor) added onto metformin (second step). When the HbA1c level continues to exceed 9%, the second therapy escalation commenced: patients switched to another dual therapy of one injection (i.e., insulin or GLP-1 receptor agonist) added onto metformin, and they stayed on this treatment for the remaining time-horizon or until death (third step). The strategies were labeled as strategy 1 to strategy 10 (Fig. 2). This study assumed that all patients persisted in the allocated three-stepwise strategy for the whole time-horizon, and the criterion for therapy switch was therapy escalation when patient’s HbA1c crosses the specified thresholds. The HbA1c thresholds for therapy escalation were set based on consultation with the doctors. The analyses were conducted based on the whole class of drugs (e.g., sulfonylurea) rather than individual drugs (e.g., glyburide). Sulfonylurea includes glyburide, glimepiride, gliclazide, glipizide, and gliquidone. Thiazolidinedione includes rosiglitazone and pioglitazone. α-Glucosidase inhibitor includes acarbose, voglibose, and miglitol. Glinide includes repaglinide, nateglinide, and mitiglinide. DPP-4 inhibitor includes sitagliptin, saxagliptin, vildagliptin, linagliptin, and alogliptin. Insulin includes various kinds of insulin and insulin analogs. GLP-1 receptor agonist includes exenatide and liraglutide [2].

### Treatment effects

There were no head-to-head studies that directly compared all the glucose-lowering treatments against each other. Thus, treatment effects of the glucose-lowering treatments were sourced from a systematic review and estimated by meta-analysis and indirect treatment comparison (Table 1). Systematic review was conducted by searching Chinese databases (China National Knowledge Infrastructure, WanFang Data, Chongqing VIP) and English databases (PubMed, Embase, Web of Science, ScienceDirect, Cochrane Library). Randomized controlled trials (RCTs) (≥ 12 weeks) published during 1990 to 2016, which estimated the treatment effect of “a glucose-lowering drug added onto metformin vs. metformin” or “metformin vs. placebo/lifestyle intervention” in Chinese T2DM patients (≥ 18 years) with and without a background of lifestyle interventions, were identified. Detailed eligibility criteria were shown in Additional file 1: Table S1. Search terms were type 2 diabetes and the targeted glucose-lowering drugs (Additional file 1: Table S2). The flow diagram of study selection was shown in Additional file 1: Fig. S1. Finally, 324 RCTs were identified [32–355]. Baseline characteristics and risk of bias of these RCTs were shown in Additional file 1: Table S3 and Fig. S2. A series of a priori random-effects meta-analyses based on the assumption of substantial variability in treatment effect size across studies [356–358] and then adjusted indirect treatment comparisons based on the Bucher method [359–361] were conducted to synthesize the absolute treatment effects of glucose-lowering treatments with and without a background of lifestyle interventions from the 324 RCTs. The framework of meta-analysis and indirect treatment comparison was shown in Additional file 1: Table S4. Meta-analysis was done in Stata/SE 15.1, and indirect treatment comparison was done in the indirect treatment comparison calculator developed by the Canadian Agency for Drugs and Technologies in Health [362, 363].

### Costs

Direct medical costs for treating T2DM and its related events were estimated. All costs were expressed in both 2019 Chinese yuan (¥) and US dollar ($). One US dollar was equal to ¥6.908 in 2019 [31].

Annual pharmacy costs of each drug class were calculated based on a simple arithmetic average of the costs of individual drugs. The cost of a drug was calculated as its retail price times its annual dose. The retail price was sourced from government medicine purchase platform [23], and the drug dose was obtained from the 324 RCTs (Table 1). A total of 19 drugs were searched in the platform, and 168 products were retrieved as a drug would have multiple products in different specifications, dose forms, or manufacturers (Additional file 1: Table S5). Insulin cost per kilogram weight per day was assumed to be ¥0.137 ($0.020) based on the inherent profile of Cardiff model.

Treatment costs of diabetes-related complications were collected from Jinan municipal claims database. The database was linked with the hospital information system of Shandong Provincial Hospital, and the claims data of adult T2DM patients who were hospitalized and had regular follow-ups for their complications at the hospital between 2013 and 2016 were extracted. The complications include ischemic heart disease, myocardial infarction, congestive heart failure, stroke, blindness, end-stage renal disease, and amputation. Two medical doctors confirmed the patients’ diagnoses of complications by checking their medical records. A total of 1164 T2DM patients with complications were retrieved. The costs associated with complications were split into fatal, non-fatal, and maintenance costs. Cost for severe hypoglycemia was sourced from an observational study in China [25]. Because symptomatic hypoglycemia and gastrointestinal reactions are usually not treated with medication and relevant published evidence was not available, their costs were assumed to be 0. BMI-related costs indicating increased prescribing costs per unit increase in BMI values were estimated from an observational study in China [28] (Table 1).

### Utilities

Utility changes associated with diabetes-related events were estimated mainly based on an EQ-5D study, which included 7081 T2DM patients who were enrolled from 75 hospitals in nine cities in China and investigated the utility values of T2DM with and without complications or comorbidities [24]. Utility changes associated with gastrointestinal reactions and BMI-related changes were not reported in this study and thus were retrieved from other sources [26, 27] (Table 1).

### Sensitivity analysis

The impact of uncertainty around model inputs was assessed by a series of univariate and probabilistic sensitivity analyses. Univariate sensitivity analyses were carried out by changing discount rate, simulation time, HbA1c thresholds for therapy escalations, risk equations, and BMI-related utilities. Besides, we also conducted a series of scenario analyses to estimate the cost-effectiveness of the 10 pharmacologic combination strategies after removing the effect of nonpharmacologic treatments. Nonpharmacologic treatments were defined as receiving lifestyle interventions and/or placebo, rather than glucose-lowering drugs. Treatment effects of the glucose-lowering treatments and nonpharmacologic treatments were also abstracted from the 324 RCTs by using the method of meta-analysis and indirect treatment comparison (Additional file 1: Table S6). The costs of the glucose-lowering treatments were the same as in the base-case analysis, and those of nonpharmacologic treatments were assumed to be 0.

## Results

### Base-case results

**Fig. 3:**
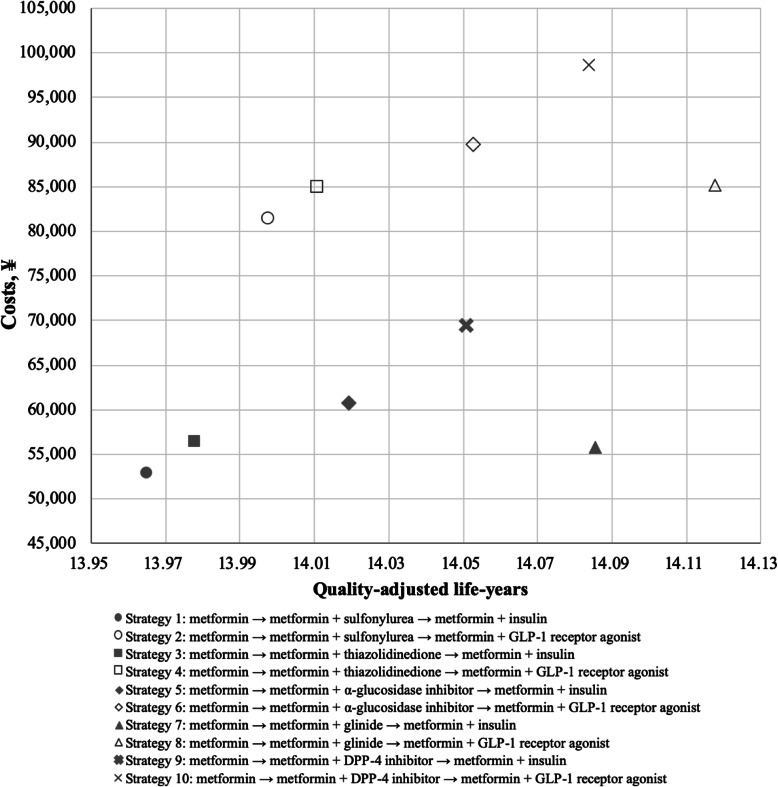
Scatter plot of incremental cost-effectiveness ratios for 10 pharmacologic combination strategies. DPP-4, dipeptidyl peptidase 4; GLP-1, glucagon-like peptide 1

All the 10 pharmacologic combination strategies showed positive effects in controlling HbA1c, cholesterol, weight, and blood pressure levels for Chinese T2DM patients (Additional file 1: Fig. S3-S6). Total costs accumulated over the lifetime ranged from ¥52,923 ($7661) to ¥98,597 ($14,273) for an individual patient under different strategies, the minimum being with strategy 1, resulting in a cost of ¥52,923 ($7661), followed by strategy 7 (¥55,729/$8067) and strategy 3 (¥56,374/ $8161), while strategy 10 resulted in the maximum costs. In terms of health benefits, overall discounted QALYs with different strategies ranged from 13.965 to 14.117 for a patient across his lifetime. Strategy 8 gained the highest QALYs of 14.117, followed by strategy 7 (14.085) and strategy 10 (14.084), while strategy 1 gained the lowest QALYs. In general, strategy 1 was associated with the lowest costs and lowest QALY gains when compared with other nine strategies, whereas strategy 10 resulted in the highest costs but incrementally less QALY gains when compared with strategy 7 and strategy 8 (Table 2, Fig. 3).

**Table 2:**
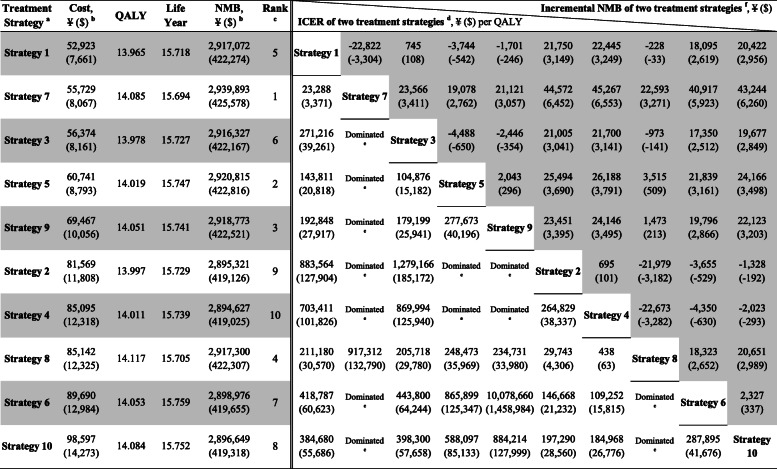
Base-case results: cost-effectiveness of 10 pharmacologic combination strategies and strategy ranking (per patient)

Strategy 1: metformin → metformin + sulfonylurea → metformin + insulin. Strategy 2: metformin → metformin + sulfonylurea → metformin + GLP-1 receptor agonist. Strategy 3: metformin → metformin + thiazolidinedione → metformin + insulin. Strategy 4: metformin → metformin + thiazolidinedione → metformin + GLP-1 receptor agonist. Strategy 5: metformin → metformin + α-glucosidase inhibitor → metformin + insulin. Strategy 6: metformin → metformin + α-glucosidase inhibitor → metformin + GLP-1 receptor agonist. Strategy 7: metformin → metformin + glinide → metformin + insulin. Strategy 8: metformin → metformin + glinide → metformin + GLP-1 receptor agonist. Strategy 9: metformin → metformin + DPP-4 inhibitor → metformin + insulin. Strategy 10: metformin → metformin + DPP-4 inhibitor → metformin + GLP-1 receptor agonist

*DPP-4* dipeptidyl peptidase 4, *GLP-1* glucagon-like peptide 1, *ICER* incremental cost-effectiveness ratio, *NMB* net monetary benefit, *QALY* quality-adjusted life-year

^a^The treatment strategies were presented in the order of ascending costs in this table. Detailed results of the 10 pharmacologic combination strategies were shown in Additional file 1: Table S8

^b^For the costs, data are 2019 Chinese yuan, ¥ (2019 US dollar, $). One US dollar was equal to ¥6.908 in 2019 [31]

^c^The strategies were ranked based on both the ICERs and the NMBs. The strategy ranking process based on the ICERs was presented in Additional file 1: Table S7. The larger the NMB value, the more cost-effective the strategy is. The ranking results based on the ICERs were equal to that based on the NMBs

^d^Results of the ICER of two treatment strategies are presented in the triangle area in the lower left corner of the table. The data indicate row-to-column differences; for example, the ICER of strategy 7 vs. strategy 1 is ¥23,288 ($3371)/QALY

^e^“Dominated” indicates a strategy (row) that is more costly and less effective than its comparator (column); for example, strategy 3 is dominated by strategy 7

^f^Results of the incremental NMB of two treatment strategies are presented in the triangle area in the upper right corner of the table (gray area). The data indicate row-to-column differences; for example, the incremental NMB of strategy 1 vs. strategy 7 is − ¥22,822 (− $3304)

Overall, strategy 7 was dominant over seven strategies (strategies 2~6, 9~10) with higher QALYs but lower costs, reporting ICERs of − ¥24,215,636 (− $3,505,448)/QALY to − ¥5983 (− $866)/QALY. Because the ICERs were ¥23,288 ($3371)/QALY when moving from strategy 1 to strategy 7 and ¥917,312 ($132,790)/QALY when moving from strategy 7 to strategy 8, strategy 7 was cost-effective compared with strategy 1 and strategy 8 at a willingness-to-pay threshold of ¥212,676 ($30,787)/QALY (Table 2). Ranking the strategies based on the ICERs, strategy 7 provided the best value for money when compared to all other strategies evaluated, followed by strategy 5, strategy 9, strategy 8, strategy 1, strategy 3, strategy 6, strategy 10, strategy 2, and strategy 4 (Additional file 1: Table S7). The results based on the NMBs confirmed the above rankings. Strategy 7 would generate additional ¥19,078 ($2762) in NMB compared to the next best alternative (strategy 5), and ¥45,267 ($6553) in NMB compared to the least cost-effective scenario (strategy 4) (Table 2).

### Sensitivity analysis results

Across all the sensitivity analyses, strategy 7 remained the most cost-effective strategy when compared to other strategies. HbA1c thresholds for therapy escalations and risk equations were influential variables for the results. In the univariate sensitivity analyses, either when the discount rate used was 3.5% [364] or when the simulation time used was 30 years, all the strategies were associated with reductions in the costs (the former, 6.7~8.6%; the latter, 4.8~10%) and the QALY gains (the former, 4.8~4.9%; the latter, 2.5~2.6%) compared with that in base case. All the strategies remained base-case ranks in the former scenario, and only the ranks of strategy 8 and 9 swapped in the latter scenario. Besides, either when applying HbA1c thresholds of 7% and 8% for second- and third-line therapy escalation or when using UKPDS 82 risk equations, all the strategies increased the QALYs (the former, 1~2%; the latter, 2~4.1%), yet also increased the costs (the former, 14.6~87.1%; the latter, 0.6~11.9%) compared with that in base case. Overall, three strategies (strategies 2, 4, 7) in the former scenario and two strategies (strategies 7, 9) in the latter scenario remained base-case ranks. When alternative BMI-related utilities were used (i.e., utility impact was 0.017 for per unit decrease in BMI and − 0.047 for per unit increase in BMI) [365], the strategies had an increase of 3.4 to 5.5% in the QALYs compared to that in base case, and four strategies (strategies 3, 6, 7, 9) kept base-case ranks. In the probabilistic sensitivity analyses, compared with that in base case, all strategies had a slight decrease of 0.4 to 0.5% in the QALYs, among which four strategies had an increase of 0.6 to 1.3% and six strategies had a decrease of 0.1 to 1% in the costs. All the strategies remained base-case ranks, which verified the base-case findings (Table 3).

**Table 3:** Sensitivity analysis results: cost-effectiveness of 10 pharmacologic combination strategies and strategy ranking (per patient)

Scenario	Item	Strategy 1	Strategy 2	Strategy 3	Strategy 4	Strategy 5	Strategy 6	Strategy 7	Strategy 8	Strategy 9	Strategy 10
Discount rate (costs and benefits) 3.5%	Cost, ¥ ($)^**a**^	49,164 (7117)	74,539 (10,790)	52,418 (7588)	77,860 (11,271)	56,539 (8185)	82,183 (11,897)	51,865 (7508)	77,920 (11,280)	64,782 (9378)	90,586 (13,113)
	QALY	13.289	13.317	13.300	13.330	13.339	13.368	13.401	13.429	13.368	13.397
	Rank^**b**^	5	9	6	10	2	7	1	4	3	8
Simulation time 30 years	Cost, ¥ ($)^**a**^	49,520 (7169)	73,411 (10,627)	52,981 (7669)	76,934 (11,137)	57,369 (8305)	81,512 (11,800)	52,475 (7596)	77,009 (11,148)	66,122 (9572)	90,417 (13,089)
	QALY	13.615	13.639	13.627	13.652	13.666	13.692	13.730	13.755	13.696	13.721
	Rank^**b**^	5	9	6	10	2	7	1	3	4	8
HbA1c thresholds of 7% and 8% for two therapy escalations	Cost, ¥ ($)^**a**^	65,349 (9460)	152,597 (22,090)	68,402 (9902)	155,817 (22,556)	72,036 (10,428)	160,022 (23,165)	66,064 (9563)	155,819 (22,556)	79,592 (11,522)	168,147 (24,341)
	QALY	14.110	14.202	14.120	14.214	14.168	14.263	14.307	14.399	14.219	14.313
	Rank^**b**^	4	9	5	10	3	8	1	6	2	7
Use UKPDS 82 risk equations to run model	Cost, ¥ ($)^**a**^	57,572 (8334)	91,305 (13,217)	61,250 (8867)	95,044 (13,759)	65,866 (9535)	99,854 (14,455)	58,536 (8474)	85,614 (12,393)	74,606 (10,800)	108,863 (15,759)
	QALY	14.529	14.567	14.532	14.570	14.557	14.595	14.587	14.397	14.606	14.643
	Rank^**b**^	2	6	5	9	4	8	1	10	3	7
Utility impact is + 0.017 and − 0.047 for per unit decrease and increase in BMI [365]	Cost, ¥ ($)^**a**^	52,923 (7661)	81,569 (11,808)	56,374 (8161)	85,095 (12,318)	60,741 (8793)	89,690 (12,984)	55,729 (8067)	85,142 (12,325)	69,467 (10,056)	98,597 (14,273)
	QALY	14.446	14.510	14.476	14.541	14.566	14.632	14.830	14.894	14.651	14.717
	Rank^**b**^	8	10	6	9	4	7	1	2	3	5
Probabilistic sensitivity analysis	Cost, ¥ ($)^**a**^	52,563 (7609)	82,077 (11,881)	56,194 (8135)	86,234 (12,483)	60,440 (8749)	90,647 (13,122)	55,150 (7983)	85,090 (12,318)	69,411 (10,048)	99,640 (14,424)
	QALY	13.897	13.930	13.911	13.944	13.953	13.988	14.022	14.056	13.986	14.019
	Rank^**b**^	5	9	6	10	2	7	1	4	3	8
Scenario analysis^**c**^	Δ Cost, ¥ ($)^**a**^	− 14,581 (− 2111)	6277 (909)	9495 (1375)	31,801 (4604)	6607 (956)	26,841 (3885)	− 14,805 (− 2143)	6542 (947)	25,963 (3758)	49,142 (7114)
	Δ QALY	0.469	0.515	0.456	0.509	0.480	0.532	0.608	0.653	0.525	0.576
	ICER, ¥ ($)/QALY	Dominant^**d**^	12,189 (1765)	20,836 (3016)	62,473 (9044)	13,754 (1991)	50,417 (7298)	Dominant^**d**^	10,018 (1450)	49,490 (7164)	85,380 (12,360)

Strategy 1: metformin → metformin + sulfonylurea → metformin + insulin. Strategy 2: metformin → metformin + sulfonylurea → metformin + GLP-1 receptor agonist. Strategy 3: metformin → metformin + thiazolidinedione → metformin + insulin. Strategy 4: metformin → metformin + thiazolidinedione → metformin + GLP-1 receptor agonist. Strategy 5: metformin → metformin + α-glucosidase inhibitor → metformin + insulin. Strategy 6: metformin → metformin + α-glucosidase inhibitor → metformin + GLP-1 receptor agonist. Strategy 7: metformin → metformin + glinide → metformin + insulin. Strategy 8: metformin → metformin + glinide → metformin + GLP-1 receptor agonist. Strategy 9: metformin → metformin + DPP-4 inhibitor → metformin + insulin. Strategy 10: metformin → metformin + DPP-4 inhibitor → metformin + GLP-1 receptor agonist

*BMI* body mass index, *DPP-4* dipeptidyl peptidase 4, *GLP-1* glucagon-like peptide 1, *HbA1c* glycosylated hemoglobin Alc, *ICER* incremental cost-effectiveness ratio, *NMB* net monetary benefit, *QALY* quality-adjusted life-year

^a^For the costs, data are 2019 Chinese yuan, ¥ (2019 US dollar, $). One US dollar was equal to ¥6.908 in 2019 [31]

^b^The strategies were ranked based on both the ICERs and the NMBs, as in the base-case analysis. The ICER is calculated as incremental costs divided by incremental QALYs of two strategies. The NMB is calculated as QALYs times willingness-to-pay/QALY, minus costs of each strategy. The larger the NMB value, the more cost-effective the strategy is. The ranking results based on the ICERs were equal to that based on the NMBs

^c^In the scenario analysis, the strategies were compared with nonpharmacologic treatment (only receiving lifestyle interventions and/or placebo, rather than glucose-lowering drugs)

^d^“Dominant” indicates a strategy that is less costly and more effective than nonpharmacologic treatment; for example, strategy 7 is dominant over nonpharmacologic treatment

In the scenario analyses, compared with nonpharmacologic treatments, all the strategies predicted fewer incidences of diabetes-related complications and mortality, thus resulting in a mean incremental QALY of 0.532 for a patient over 40 years, by a range of 0.456 QALYs with strategy 3 to 0.653 QALYs with strategy 8. Two strategies reduced costs by ¥14,581 ($2111) with strategy 1 and ¥14,805 ($2143) with strategy 7, and eight strategies increased costs by ¥6277 ($909) with strategy 2 to ¥49,142 ($7114) with strategy 10. Consequently, two strategies (strategies 1, 7) were cost-saving with higher QALY gains but lower costs, and eight strategies were cost-effective with an ICER of ¥10,018 ($1450)/QALY for strategy 8 to ¥85,380 ($12,360)/QALY for strategy 10, when compared to nonpharmacologic treatments (Table 3).

## Discussion

There are multiple glucose-lowering treatments in China; however, their effectiveness and especially the costs vary from each other. Choosing high efficacious, safe, and affordable treatments is the key for most T2DM patients to insist on long-term medication. This was the first study to estimate and compare the lifetime cost-effectiveness of 10 commonly used pharmacologic combination strategies for T2DM patients in China.

Results showed that based on current clinical practice, strategy 7—involving the sequential addition of glinide and insulin to first-line metformin therapy—was the most cost-effective strategy, while strategy 4—involving the sequential addition of thiazolidinedione and GLP-1 receptor agonist to first-line metformin therapy—was the least cost-effective strategy when compared with all other strategies evaluated. All five strategies that use metformin plus GLP-1 receptor agonist as third line were associated with higher costs compared to those that use metformin plus insulin as third line. When keeping the second-line therapy the same, a strategy using metformin plus GLP-1 receptor agonist as third line resulted in both higher QALYs and higher costs compared with a strategy using metformin plus insulin. When keeping the third-line therapy the same, addition of glinide to metformin as second line was associated with the highest QALY gains, followed by addition of DPP-4 inhibitor, α-glucosidase inhibitor, thiazolidinedione, and sulfonylurea; conversely, addition of sulfonylurea to metformin resulted in the lowest costs, followed by addition of glinide or thiazolidinedione, and DPP-4 inhibitor was related to the highest costs. Previous study reported that the most commonly used dual therapy was metformin plus sulfonylureas, followed by metformin plus glinides in a real-world setting in China [3]. Our findings that metformin plus sulfonylureas costs less than other dual therapies may somewhat account for this phenomenon. However, if patients want more health benefits, it is of value to try metformin plus glinide.

Sensitivity analyses somewhat confirmed the base-case results. All the strategies remained base-case ranks in the probabilistic sensitivity analyses and in the univariate sensitivity analyses where the discount rate used was 3.5%. Although the ranks of some strategies changed in other sensitivity analyses, strategy 7 remained the most cost-effective strategy when compared to other strategies. This implied that the ranking results should be used cautiously to avoid misleading conclusions instead of denying their actual value for decision-makers. Besides, prescribers should additionally take into consideration patients’ individual needs, preferences, and values, because a patient-centered approach is recommended in choosing pharmacologic treatments for T2DM [366, 367]. Whatever, our results may have a practical importance, as healthcare services are mainly provided by public medical institutions, and value-based price negotiations are increasingly used for reimbursement approvals in China. Because there are many patients not aware of the importance of persistent pharmacologic treatments for T2DM in China [368], we additionally quantified the long-term impact of pharmacologic treatments on disease burden of T2DM after removing the effect of nonpharmacologic treatments. A patient persisted in pharmacologic combination treatments over 40 years was projected to obtain an incremental benefit of 0.532 QALYs at an acceptable range of cost increase or even at cost saving, compared with those who did not receive pharmacologic treatments.

In the past decade, several studies in China have introduced economic simulation models to predict and compare the long-term costs and benefits of several glucose-lowering therapies for T2DM. Palmer et al., Wu et al., Li et al., and Chen et al. evaluated the cost-effectiveness of different types of insulins using CORE diabetes model [369–373]. Gu et al. and Shao et al. estimated the cost-effectiveness of saxagliptin (or dapagliflozin) vs. acarbose (or glimepiride), and exenatide vs. insulin glargine using the Cardiff diabetes model [374–378]. Zhu and Chen assessed the cost-effectiveness of vildagliptin vs. pioglitazone vs. glimepiride, and sitagliptin vs. glimepiride vs. acarbose as add-on therapy to metformin using UKPDS model [379, 380]. However, all previous studies focused on comparing several individual drugs as monotherapy or add-on therapy at a specific disease stage and keeping therapies at other disease stages the same. Besides, they all used utility data from foreign populations to calculate QALYs. Unlike them, our study first included almost all individual drugs available in China to achieve comparisons between different drug classes; second, we targeted at holistic treatment strategies over lifelong disease stages; third, we used utilities of Chinese patients for the first time. Therefore, our study was poorly comparable with previous studies. Only one study was found to compare the short-term cost-effectiveness of six classes of oral glucose-lowering drugs in China, based on daily pharmacy costs and blood glucose reductions. Result showed that thiazolidinedione was most cost-effective in reducing fasting and 2 h postprandial blood glucose, followed by biguanide, glinide, sulfonylurea, α-glucosidase inhibitor, and Chinese traditional medicine [7]. However, this study was also not comparable with our study.

There were several limitations in this study. First, systematic review and meta-analysis have some inherent methodological challenges, such as low study quality, potential publication bias, and unexplainable heterogeneity, that might affect the quality of clinical evidence. Besides, because there was no direct clinical evidence for comparing different dual therapies against each other, indirect treatment comparison was used, which might introduce more uncertainties around synthesized estimates compared with a direct head-to-head estimate. Second, since there are no published risk equations based on the Chinese population, the UKPDS risk equations were used in our study. However, our study population and the UKPDS population somewhat differed. On the one hand, the UKPDS population consists of 82% white, 10% Asian Indian, and 8% Afro-Caribbean patients with newly diagnosed T2DM [13, 381], while our population were Chinese patients with newly diagnosed T2DM. On the other hand, the baseline risk factors, such as HbA1c level (8.4% in our population vs. 7.1% in UKPDS population), between the two populations were not fully identical. Thus, it is uncertain whether the UKPDS risk equations may fit the Chinese patients very well. Future studies should pay attention to this issue. Third, our analyses were conducted based on the whole class of drugs. However, not all drugs within a class have the same efficacy, especially individual insulins and GLP-1 receptor agonists. Thus, the efficacy used in our study might be biased, depending on which individual drugs and RCTs have been included in the meta-analysis. Besides, patients are prescribed an individual drug rather than an average drug within a class in the real world, which might limit the generalizability of our results. Fourth, because the Cardiff model is not able to model more than three escalation steps, treatment strategies in our study are only composed of a three-stepwise treatment path, so that other relevant and longer treatment paths were not evaluated which may alter the findings of the study. For the same reason, although the use of insulins is stepwise (e.g., usually basal insulin is initiated first, followed by bolus insulin), we simply included all types of insulins into one insulin class. Fifth, evidence showed that insufficient efficacy was the main reason for treatment alteration in China, followed by adverse reaction [3]. Our study assumed that therapy escalation occurs when patient’s HbA1c fails to be controlled. However, as there is a lack of China-specific evidence on treatment alteration induced by adverse reaction of every study therapy, we did not include this in our analysis. Sixth, patients starting injectable therapy in the model are at a much later stage of the disease, while the patients in the RCTs informing this treatment step might be at an earlier stage of the disease; thus, they are likely at a different risk level. Seventh, since there is a lack of China-specific disutility values for blindness and end-stage renal disease, we conservatively used that of retinopathy and nephropathy as alternatives. This may lead to an underestimation of both complications on health. Lastly, because sodium-glucose cotransporter 2 inhibitors were not included in the Chinese clinical guideline at the time of our study, we did not include this drug class.

## Conclusions

This study provided comprehensive evidence on costs and benefits of 10 commonly used pharmacologic combination strategies for patients, clinicians, and healthcare decision-makers within a unified hierarchy. This could improve their understanding of the cost-effectiveness of the existing treatment strategies in China, and help them choose high cost-effective treatments based on individual patient’s preference and need in clinical practice. In general, it is found that a strategy starting with metformin, then transitioning to metformin plus glinide as second line and finally using metformin plus insulin as third line, provided the best value for money when compared to all other strategies evaluated. Besides, this study highlighted the importance of promoting timely, rational, and sustained pharmacologic treatments in reducing patients’ disease burden. Our findings can be used to improve clinical guidelines, design the essential drug formulary, infer clinical practice, and help the decision-maker to design reimbursement policy.

## Supplementary Information

**Additional file 1:** **Table S1.** Eligibility criteria. **Table S2.** Full electronic search strategy for PubMed. **Table S3.** Baseline characteristics of studies included in the meta-analysis and indirect treatment comparison. **Table S4.** Framework for estimation of absolute treatment effects of glucose-lowering treatments based on meta-analysis and indirect treatment comparison. **Table S5.** Retail prices of the glucose-lowering drugs collected from government medicine purchase platform. **Table S6.** Treatment effects of glucose-lowering treatments and nonpharmacologic treatment in the scenario analyses. **Table S7.** Base-case results: cost-effectiveness of ten pharmacologic combination strategies and strategy ranking process based on the ICERs (per patient). **Table S8.** Detailed results for the ten pharmacologic combination strategies: base-case analysis. **Fig. S1.** PRISMA flow diagram of study selection. **Fig. S2.** Risk of bias graph. **Fig. S3.** The trajectories of HbA1c in ten pharmacologic combination strategies over time: base-case analysis. **Fig. S4.** The trajectories of cholesterol in ten pharmacologic combination strategies over time: base-case analysis. **Fig. S5.** The trajectories of weight in ten pharmacologic combination strategies over time: base-case analysis. **Fig. S6.** The trajectories of SBP in ten pharmacologic combination strategies over time: base-case analysis.

## Data Availability

The datasets used and/or analyzed during the current study are available from the corresponding author on reasonable request.

## Abbreviations

BMI: Body mass index
DPP-4: Dipeptidyl peptidase 4
GLP-1: Glucagon-like peptide 1
HbA1c: Glycosylated hemoglobin Alc
HDL-C: High-density lipoprotein-cholesterol
ICER: Incremental cost-effectiveness ratio
LDL-C: Low-density lipoprotein-cholesterol
NMB: Net monetary benefit
QALY: Quality-adjusted life-year
SBP: Systolic blood pressure
SE: Standard error
TC: Total cholesterol
T2DM: Type 2 diabetes
UKPDS: United Kingdom Prospective Diabetes Studies
