# Supplementary material for: Choice across 10 pharmacologic combination strategies for type 2 diabetes: a cost-effectiveness analysis
Source: BMC Med. 2020 Dec 3;18:378. doi: 10.1186/s12916-020-01837-x (PMC7713153; doi:10.1186/s12916-020-01837-x)
Supplement: Supplementary file 1 — Additional file 1: Table S1. Eligibility criteria. Table S2. Full electronic search strategy for PubMed. Table S3. Baseline characteristics of studies included in the meta-analysis and indirect treatment comparison. Table S4. Framework for estimation of absolute treatment effects of glucose-lowering treatments based on meta-analysis and indirect treatment comparison. Table S5. Retail prices of the glucose-lowering drugs collected from government medicine purchase platform. Table S6. Treatment effects of glucose-lowering treatments and nonpharmacologic treatment in the scenario analyses. Table S7. Base-case results: cost-effectiveness of ten pharmacologic combination strategies and strategy ranking process based on the ICERs (per patient). Table S8. Detailed results for the ten pharmacologic combination strategies: base-case analysis. Fig. S1. PRISMA flow diagram of study selection. Fig. S2. Risk of bias graph. Fig. S3. The trajectories of HbA1c in ten pharmacologic combination strategies over time: base-case analysis. Fig. S4. The trajectories of cholesterol in ten pharmacologic combination strategies over time: base-case analysis. Fig. S5. The trajectories of weight in ten pharmacologic combination strategies over time: base-case analysis. Fig. S6. The trajectories of SBP in ten pharmacologic combination strategies over time: base-case analysis. [file 12916_2020_1837_MOESM1_ESM.docx]

Content

[Table S1. Eligibility criteria 2](#_Toc50901318)

[Table S2. Full electronic search strategy for PubMed 3](#_Toc50901319)

[Table S3. Baseline characteristics of studies included in the meta-analysis and indirect treatment comparison 4](#_Toc50901320)

[Table S4. Framework for estimation of absolute treatment effects of glucose-lowering treatments based on meta-analysis and indirect treatment comparison 36](#_Toc50901322)

[Table S5. Retail prices of the glucose-lowering drugs collected from government medicine purchase platform 37](#_Toc50901323)

[Table S6. Treatment effects of glucose-lowering treatments and nonpharmacologic treatment in the scenario analyses 44](#_Toc50901324)

[Table S7. Base-case results: cost-effectiveness of ten pharmacologic combination strategies and strategy ranking process based on the ICERs (per patient) 46](#_Toc50901325)

[Table S8. Detailed results for the ten pharmacologic combination strategies: base-case analysis 49](#_Toc50901326)

[Fig. S1. PRISMA flow diagram of study selection. 54](#_Toc50901327)

[Fig. S2. Risk of bias graph 55](#_Toc50901328)

[Fig. S3. The trajectories of HbA1c in ten pharmacologic combination strategies over time: base-case analysis 56](#_Toc50901329)

[Fig. S4. The trajectories of cholesterol in ten pharmacologic combination strategies over time: base-case analysis. 57](#_Toc50901330)

[Fig. S5. The trajectories of weight in ten pharmacologic combination strategies over time: base-case analysis. 58](#_Toc50901331)

[Fig. S6. The trajectories of SBP in ten pharmacologic combination strategies over time: base-case analysis. 59](#_Toc50901332)

**Table S1. Eligibility criteria**

| (1) Participants were Chinese patients with type 2 diabetes (≥18 years).  (2) For ‘metformin vs placebo/lifestyle intervention’ studies: intervention was metformin monotherapy; comparison was placebo or lifestyle intervention like diet and/or physical exercise. Other drugs were not allowed.  (3) For ‘a glucose-lowering drug added onto metformin vs metformin’ studies: interventions were a glucose-lowering agent added to metformin, and the add-on agents include sulfonylureas (glyburide, glimepiride, gliclazide, glipizide, gliquidone), thiazolidinediones (rosiglitazone, pioglitazone), α-glucosidase inhibitors (acarbose, voglibose, miglitol), glinides (repaglinide, nateglinide, mitiglinide), DPP-4 inhibitors (sitagliptin, saxagliptin, vildagliptin, linagliptin, alogliptin), GLP-1 receptor agonists (exenatide, liraglutide), and insulins (various kinds of insulin and insulin analogs); comparison was metformin monotherapy. Other drugs were not allowed. Total sample size of the study was ≥60.  (4) Background intervention was limited to lifestyle intervention like diet and/or physical exercise.  (5) Main outcomes were the change-from-baseline in glycosylated hemoglobin Alc, body mass index, total cholesterol, high density lipoprotein-cholesterol and systolic blood pressure. Ensure outcome was the incidences of hypoglycemia.  (6) Study design was randomized controlled trial.  (7) Length of follow-up was ≥12 weeks.  (8) Study was written in Chinese or English. |
| --- |

**Table S2. Full electronic search strategy for PubMed**

| (((((("Diabetes Mellitus, Type 2"[Mesh]) OR type 2 diabetes)) AND ((((((((((((((((((((((((((((((((((((((((((((("Metformin"[Mesh]) OR metformin) OR "Glyburide"[Mesh]) OR glyburide) OR glibenclamide) OR "glimepiride" [Supplementary Concept]) OR glimepiride) OR "Gliclazide"[Mesh]) OR gliclazide) OR "Glipizide"[Mesh]) OR glipizide) OR "gliquidone" [Supplementary Concept]) OR gliquidone) OR "repaglinide" [Supplementary Concept]) OR repaglinide) OR "nateglinide" [Supplementary Concept]) OR nateglinide) OR "mitiglinide" [Supplementary Concept]) OR mitiglinide) OR "Acarbose"[Mesh]) OR acarbose) OR "voglibose" [Supplementary Concept]) OR voglibose) OR "miglitol" [Supplementary Concept]) OR miglitol) OR "Sitagliptin Phosphate"[Mesh]) OR sitagliptin) OR "saxagliptin" [Supplementary Concept]) OR saxagliptin) OR "vildagliptin" [Supplementary Concept]) OR vildagliptin) OR "Linagliptin"[Mesh]) OR linagliptin) OR "alogliptin" [Supplementary Concept]) OR alogliptin) OR "rosiglitazone" [Supplementary Concept]) OR rosiglitazone) OR "pioglitazone" [Supplementary Concept]) OR pioglitazone) OR "Insulin"[Mesh]) OR insulin) OR "exenatide" [Supplementary Concept]) OR exenatide) OR "Liraglutide"[Mesh]) OR liraglutide)) AND ((((("China"[Mesh] OR "Taiwan"[Mesh])) OR China) OR "Asian Continental Ancestry Group"[Mesh]) OR Chinese))) AND ("1990/01/01"[Date - Publication] : "2016/12/31"[Date - Publication]) Filters: Humans; Chinese; English; Adolescent: 13-18 years; Adult: 19+ years |
| --- |

**Table S3. Baseline characteristics of studies included in the meta-analysis and indirect treatment comparison**

| **Study** |  | **Background intervention** | **Study duration, w** | **Intervention** | | | | | | | **Comparison** | | | | | |
| --- | --- | --- | --- | --- | --- | --- | --- | --- | --- | --- | --- | --- | --- | --- | --- | --- |
|  |  |  |  | **Treatment** | **Sample size, n** | **Female, n** | **Age, y** | **Diabetes duration, y** | **Metformin dose, mg/d** | **Add-on drug dose, mg/d** | **Treatment** | **Sample size, n** | **Female, n** | **Age, y** | **Diabetes duration, y** | **Drug dose, mg/d** |
| Bai N 2016 | [23] | Diet+exercise | 12 | Metformin | 45 | 17 | 54 (8.28) | NR | 1500 | —— | Diet+exercise | 45 | 19 | 54 (8.12) | NR | None |
| Ruan Y 2015 | [24] | Diet+exercise | 12 | Metformin | 53 | 22 | 55.1 (8) | NR | 1500 | —— | Diet+exercise | 53 | 24 | 54.3 (7.9) | NR | None |
| Guo W 2014 | [25] | Diet+exercise | 12 | Metformin | 28 | 13 | 49.7 (6.8) | Newly diagnosed | 1500 | —— | Diet+exercise | 28 | 12 | 50.4 (6.4) | Newly diagnosed | None |
| Li L 2014 | [26] | Diet+exercise | 12 | Metformin | 16 | 6 | 49.5 (11.7) | 1.2 (0.9) | 1500 | —— | Diet+exercise | 12 | 4 | 49.5 (11.7) | 1.2 (0.9) | None |
| Guo DL 2014 | [27] | Diet+exercise | 26 | Metformin | 60 | 20 | 44.9 | NR | Initial:500, Max:2000 | —— | Diet+exercise | 60 | 28 | 44.9 | NR | None |
| Yang WC 2013a | [28] | Diet | 12 | Metformin | 22 | 11 | NR | 2 (1.2) | 1000 | —— | Diet+exercise | 19 | 11 | NR | 2 (1.2) | None |
| Yang WC 2013b | [28] | Diet+exercise | 12 | Metformin | 19 | 9 | NR | 2 (1.2) | 1000 | —— | Diet+exercise | 19 | 11 | NR | 2 (1.2) | None |
| Liu YT 2013 | [29] | Diet | 14 | Metformin | 38 | 21 | 53.12 (8.29) | NR | Initial:500, Max:1500 | —— | Diet+exercise | 35 | 13 | 58.64 (7.71) | NR | None |
| Xi Y 2013 | [30] | Diet+exercise | 16 | Metformin | 20 | 10 | 51 (4) | Newly diagnosed | 2000 | —— | Diet+exercise | 20 | 9 | 51 (7) | Newly diagnosed | None |
| Chen Y 2008 | [31] | Diet+exercise | 16 | Metformin | 17 | 8 | 42 (4.7) | Newly diagnosed | 1000 | —— | Diet+exercise | 17 | 7 | 44 (5.2) | Newly diagnosed | None |
| Deng HO 2007a | [32] | None | 12 | Metformin | 20 | 0 | 67 (2.3) | Newly diagnosed | 1000 | —— | Diet | 20 | 0 | 68 (2.1) | Newly diagnosed | None |
| Deng HO 2007b | [32] | None | 12 | Metformin | 20 | 0 | 66 (2.2) | Newly diagnosed | 1500 | —— | Diet | 20 | 0 | 68 (2.1) | Newly diagnosed | None |
| Li HZ 2007 | [33] | Diet+exercise | 12 | Metformin | 41 | 17 | 47.5 (12.6) | 0.3 (0.1) | 1500 | —— | Diet+exercise | 38 | 18 | 46.2 (10.4) | 0.32 (0.17) | None |
| Mei Q 2006 | [34] | Diet+exercise | 16 | Metformin | 46 | 21 | 46.3 | Newly diagnosed | 1500 | —— | Diet+exercise | 40 | 18 | 46.3 | Newly diagnosed | None |
| Wang L 2006 | [35] | Diet+exercise | 24 | Metformin | 26 | 10 | 47.8 (1.8) | NR | 1500 | —— | Placebo | 16 | 8 | 46.6 (2.4) | NR | 6pills/d |
| Wu GT 2001 | [36] | Diet+exercise | 24 | Metformin | 60 | 25 | 49.8 (6.7) | 4.2 (2.1) | 1000 | —— | Placebo | 60 | 23 | 50.4 (6.4) | 4.3 (2.4) | 1000 |
| Qu JC 2011 | [37] | Diet+exercise | 12 | Metformin | 28 | NR | 45 (9) | Newly diagnosed | 1000 | —— | Diet+exercise | 29 | NR | 45 (9) | Newly diagnosed | None |
| Guo M 2014 | [38] | None | 24 | Metformin | 29 | 12 | 54.7 (7.3) | NR | Initial:1000  1w later:1500  3w later:2000 | —— | Placebo | 29 | 10 | 53.3 (7.3) | NR | Initial:1000  1w later:1500  3w later:2000 |
| **Study** |  | **Background intervention** | **Study duration, w** | **Intervention** | | | | | | | **Comparison** | | | | | |
|  |  |  |  | **Treatment** | **Sample size, n** | **Female, n** | **Age, y** | **Diabetes duration, y** | **Metformin dose, mg/d** | **Add-on drug dose, mg/d** | **Treatment** | **Sample size, n** | **Female, n** | **Age, y** | **Diabetes duration, y** | **Drug dose, mg/d** |
| Wang JP 2016 | [39] | Diet+exercise | 12 | Metformin+ Acarbose | 34 | 20 | 49.5 (1.5) | 6.9 (1.7) | Initial:1500, max:2000 | 150 | Metformin | 34 | 20 | 50.2 (1.7) | 5.3 (2.5) | 1500, max:2000 |
| Han JK 2016 | [40] | Diet+exercise | 12 | Metformin+ Acarbose | 59 | 26 | 51.03 (9.48) | 5.89 (1.26) | 1500 | 150 | Metformin | 59 | 26 | 51.03 (9.48) | 5.89 (1.26) | 1500 |
| Wang HY 2016 | [41] | Diet+exercise | 12 | Metformin+ Acarbose | 36 | 15 | 51.5 (2.6) | NR | 1500 | 150~600 | Metformin | 34 | 15 | 50.7 (3.7) | NR | 1500 |
| Kong LL 2016 | [42] | Diet+exercise | 12 | Metformin+ Acarbose | 43 | 21 | 59.5 (9.2) | NR | 1500 | 150 | Metformin | 43 | 19 | 62 (9.5) | NR | 1500 |
| Zeng BF 2016 | [43] | Diet+exercise | 12 | Metformin+ Acarbose | 66 | 23 | 53.2 (1.9) | 5.7 (1.3) | 1500 | 150 | Metformin | 66 | 25 | 52.5 (2.6) | 5.3 (1.9) | 1500 |
| Liu J 2015 | [44] | Diet+exercise | 12 | Metformin+ Acarbose | 33 | 11 | 50.2 (10.5) | 5.7 (1.5) | 1000 | 150 | Metformin | 33 | 14 | 49.3 (11.8) | 5.2 (2.3) | 1000 |
| Wang XQ 2015 | [45] | Diet+exercise | 12 | Metformin+ Acarbose | 40 | 40 | 52.8 (4.9) | 2.3 (1.4) | 1500 | 150 | Metformin | 40 | 40 | 52.8 (4.9) | 2.3 (1.4) | 1500 |
| Li QD 2015 | [46] | Diet+exercise | 12 | Metformin+ Acarbose | 78 | 31 | 52.2 (2.6) | 14.4 (6.3) | 1500 | 150 | Metformin | 78 | 33 | 52.7 (2.5) | 14.1 (7.1) | 1500 |
| Liu P 2015 | [47] | Diet+exercise | 12 | Metformin+ Acarbose | 60 | 27 | 38.7 (3.2) | 6.4 (2.3) | Initial:750, max:2000 | 150 | Metformin | 60 | 28 | 37.7 (4.3) | 6.2 (2.7) | Initial:750, max:2000 |
| Huang LY 2015 | [48] | Diet+exercise | 12 | Metformin+ Acarbose | 57 | 24 | 52.15 (1.25) | 5.74 (1.36) | 1500 | 150 | Metformin | 57 | 25 | 56.36 (2.36) | 5.6 (1.32) | 1500 |
| Su ZL 2015 | [49] | Diet+exercise | 12 | Metformin+ Acarbose | 36 | 15 | 54.9 (1.5) | 7.6 (1.6) | 1000~2000 | 150 | Metformin | 36 | 14 | 53.9 (1.9) | 5.6 (2.1) | 1000~2000 |
| A YC 2015 | [50] | Diet+exercise | 26 | Metformin+ Acarbose | 41 | 18 | 57.26 (10.37) | 10.08 (1.87) | 500~1000 | 150 | Metformin | 41 | 17 | 57.95 (11.16) | 10.03 (1.65) | 500~1000 |
| Tan YL 2014 | [51] | NR | 12 | Metformin+ Acarbose | 262 | 101 | 68.1 (10.1) | 5.8 (2.3) | 1500 | 150 | Metformin | 262 | 130 | 69.2 (9.2) | 6.4 (1.9) | 1500 |
| Zheng Q 2014 | [52] | NR | 24 | Metformin+ Acarbose | 92 | 40 | 60.79 (7.02) | 8.39 (4.02) | Initial:1500, max:2000 | 150 | Metformin | 92 | 36 | 61.85 (7.23) | 8.55 (4.23) | Initial:1500, max:2000 |
| Zeng XY 2014 | [53] | Diet+exercise | 12 | Metformin+ Acarbose | 44 | 13 | 69.21 (3.16) | 4.12 (1.35) | 1500 | 150 | Metformin | 44 | 14 | 69.93 (3.21) | 4.32 (1.41) | 1500 |
| Zhou XH 2013 | [54] | Diet+exercise | 12 | Metformin+ Acarbose | 40 | 15 | 50.8 (10.2) | 6.3 (1.8) | 1500 | 150 | Metformin | 40 | 14 | 48.1 (11.3) | 5.1 (2.9) | 1500 |
| Liang JH 2012 | [55] | Diet+exercise | 12 | Metformin+ Acarbose | 36 | 14 | 55.4 (1.2) | 3 (1.3) | 1000~2000 | 150 | Metformin | 36 | 16 | 54.8 (1.8) | 3 (1.2) | 1000~2000 |
| Zhang MH 2011 | [56] | Diet+exercise | 12 | Metformin+ Acarbose | 30 | NR | 35~60 | Newly diagnosed | 1500 | 150 | Metformin | 30 | NR | 35~60 | Newly diagnosed | 1500 |
| Zhu ZL 2011 | [57] | Diet+exercise | 26 | Metformin+ Acarbose | 31 | 13 | 52 (10) | Newly diagnosed | 1500 | 150 | Metformin | 33 | 14 | 52 (10) | Newly diagnosed | 1500 |
| Zheng YB 2010 | [58] | Diet+exercise | 16 | Metformin+ Voglibose | 30 | 14 | 43.3 (11.7) | Newly diagnosed | 750~1500 | 0.6 | Metformin | 30 | 16 | 44.8 (10.5) | Newly diagnosed | 750~1500 |
| He K 2016 | [59] | NR | 12 | Metformin+ Miglitol | 62 | 29 | 65.15 (6.36) | 5.28 (2.15) | 500~4500 | Initial:75, later:150 | Metformin | 62 | 30 | 65.87 (6.14) | 5.94 (2.84) | 500~4500 |
| Huang Y 2015 | [60] | Diet+exercise | 12 | Metformin+ Miglitol | 36 | 14 | 60.31 (11.92) | NR | 1000 | 150 | Metformin | 36 | 15 | 59.24 (10.75) | NR | 1000 |
| He K 2014 | [61] | Diet+exercise | 12 | Metformin+ Miglitol | 50 | 21 | 53.1 (9.3) | NR | 1500 | 150 | Metformin | 50 | 23 | 52.4 (9.6) | NR | 1500 |
| Shao HM 2015 | [62] | NR | 12 | Metformin+ Glyburide | 41 | 15 | 44.1 (7) | 5.8 (2.3) | 1500~2000 | Initial:1 | Metformin | 42 | 15 | 45.1 (7.5) | 5.8 (2.7) | 1500~2000 |
| Mao ZH 2016 | [63] | NR | 12 | Metformin+ Glimepiride | 50 | 18 | 46.2 (10.5) | 6.3 (1.2) | 1500 | Initial:1, max:6 | Metformin | 50 | 22 | 48.8 (8.3) | 6.2 (1.3) | 1500 |
| Xu YX 2016 | [64] | Diet+exercise | 12 | Metformin+ Glimepiride | 34 | 9 | 54.36 (2.51) | 3.51 (0.51) | Initial:500, max:2000 | Initial:1~2, max:4 | Metformin | 34 | 10 | 54.26 (2.45) | 3.64 (0.42) | Initial:500, max:2000 |
| Ma CH 2016a | [65] | Diet+exercise | 12 | Metformin+ Glimepiride | 30 | 12 | 45 (9) | 6 (3) | 1500 | 2 | Metformin | 30 | 14 | 44 (10) | 5 (3) | 1500 |
| Yang GM 2016 | [66] | Diet+exercise | 14 | Metformin+ Glimepiride | 50 | 28 | 49.4 | 2.5 | 750 | Initial:1, max:3 | Metformin | 50 | 27 | 48.8 | 2.6 | 750 |
| Yang QF 2016a | [67] | NR | 15 | Metformin+ Glimepiride | 140 | 68 | 58.05 (10.93) | NR | 2000 | 2~4 | Metformin | 70 | 34 | 57.39 (12.24) | NR | 2000 |
| Xu YJ 2015 | [68] | NR | 12 | Metformin+ Glimepiride | 39 | 20 | 50.3 (8.4) | NR | 1500 | Initial:1, max:6 | Metformin | 39 | 21 | 51.2 (8.2) | NR | 1500 |
| Sun SJ 2014 | [69] | NR | 12 | Metformin+ Glimepiride | 36 | ? | 47.5 (5.5) | Newly diagnosed | 1500 | Initial:1 | Metformin | 32 | ? | 47.5 (5.5) | Newly diagnosed | 1500 |
| Li X 2012 | [70] | Diet+exercise | 12 | Metformin+ Glimepiride | 42 | 18 | 57.6 (7.3) | 4.9 (1.2) | 1500 | Initial:1, max:6 | Metformin | 42 | 18 | 57.6 (7.3) | 4.9 (1.2) | 1500 |
| Wang Y 2012 | [71] | Diet+exercise | 12 | Metformin+ Glimepiride | 40 | 10 | 49.3 (4.3) | Newly diagnosed | 750 | Initial:3, max:6 | Metformin | 40 | 5 | 50.2 (4.3) | Newly diagnosed | 750 |
| Zhao HY 2012a | [72] | Diet+exercise | 12 | Metformin+ Glimepiride | 45 | 20 | 40.7 (18.3) | 5.4 (4.7) | 750 | 2 | Metformin | 45 | 20 | 40.7 (18.3) | 5.4 (4.7) | 750 |
| Ning J 2006 | [73] | NR | 52 | Metformin+ Glimepiride | 51 | 26 | 52.66 (9.62) | NR | Initial:500, max:2250 | Initial:1, max:6 | Metformin | 50 | 19 | 52.66 (9.62) | NR | Initial:500, max:2250 |
| Wang J 2015 | [74] | Diet+exercise | 12 | Metformin+ Glimepiride | 74 | 30 | 53.5 (2.4) | 10.5 (1.4) | 1500 | Initial:1, max:8 | Metformin | 74 | 30 | 53.5 (2.4) | 10.5 (1.4) | 1500 |
| Su YS 2016 | [75] | Diet+exercise | 26 | Metformin+ Gliclazide | 50 | 24 | 58.54 (9.74) | 1~21 | Initial:250, 4w later:1500 | Initial:30, 4w later:60 | Metformin | 50 | 24 | 58.54 (9.74) | 1~21 | Initial:250, 4w later:1500 |
| Wei J 2015 | [76] | NR | 12 | Metformin+ Gliclazide | 36 | 16 | 42 (2.41) | NR | 1000 | NR | Metformin | 36 | 17 | 45 (2.36) | NR | 1000 |
| Guo YP 2015 | [77] | Diet | 26 | Metformin+ Gliclazide | 41 | 18 | 57.4 (4.3) | 5.4 (0.8) | Initial:250, later:1500 | Initial:30, later:60 | Metformin | 41 | 18 | 57.4 (4.3) | 5.4 (0.8) | Initial:250, later:1500 |
| Qin SL 2014 | [78] | Diet+exercise | 12 | Metformin+ Gliclazide | 45 | 21 | 40.2 (3) | Newly diagnosed | Initial:1000, 1w later:1500 | 30 | Metformin | 45 | 23 | 39.2 (3.3) | Newly diagnosed | Initial:1000, 1w later:1500 |
| Luo JY 2014 | [79] | NR | 12 | Metformin+ Gliclazide | 50 | 30 | 43.3 (5.81) | 3~11 | 750 | Initial:30~60, max:120 | Metformin | 50 | 29 | 44.2 (6.72) | 3~11 | 750 |
| Wei J 2013 | [80] | Diet+exercise | 12 | Metformin+ Gliclazide | 36 | 17 | 54.9 (2.4) | 9.5 (2.9) | 750 | 60 | Metformin | 24 | 11 | 55.4 (2.8) | 10.2 (2.4) | 1500 |
| Zhang JF 2013 | [81] | NR | 26 | Metformin+ Gliclazide | 48 | 23 | 54.3 (4.8) | 1~6 | Initial:250, 4w later:1500 | Initial:30, 4w later:60 | Metformin | 48 | 23 | 54.3 (4.8) | 1~6 | Initial:250, 4w later:1500 |
| He F 2013 | [82] | NR | 26 | Metformin+ Gliclazide | 43 | 16 | 50.2 (7.5) | 4.6 (1.5) | 1500 | Initial:30, max:120 | Metformin | 43 | 16 | 50.2 (7.5) | 4.6 (1.5) | 1500 |
| Teng YP 2012A | [83] | Diet+exercise | 12 | Metformin+ Gliclazide | 43 | 21 | 38.2 (3.2) | Newly diagnosed | 1000~1500 | 30 | Metformin | 43 | 21 | 38.2 (3.2) | Newly diagnosed | 1000~1500 |
| Luo ZZ 2012 | [84] | Diet+exercise | 16 | Metformin+ Gliclazide | 50 | 27 | 45.1 (7.8) | 0.17~10 | 1500 | 30 | Metformin | 50 | 25 | 43.2 (8.1) | 0.17~10 | 1500 |
| Zhong XX 2012 | [85] | Diet+exercise | 12 | Metformin+ Gliclazide | 63 | 27 | 46.8 (14.7) | 4.6 (1.2) | 2250 | Initial:30~60, max:120 | Metformin | 63 | 27 | 46.8 (14.7) | 4.6 (1.2) | 2250 |
| Xu QL 2007 | [86] | Diet+exercise | 12 | Metformin+ Gliclazide | 42 | 22 | 57.4 (4.1) | 7.57 (0.58) | Initial:500 | Initial:80 | Metformin | 40 | 21 | 57.9 (4.2) | 7.52 (0.59) | Initial:500 |
| Xu H 2015 | [87] | NR | 12 | Metformin+ Gliclazide | 51 | 25 | 56.57 (13.48) | NR | 750 | 30~120 | Metformin | 51 | 25 | 56.57 (13.48) | NR | 750 |
| Mei Z 2014 | [88] | Diet+exercise | 12 | Metformin+ Glipizide | 40 | 20 | 44.3 (5.6) | 0.56 (0.2) | 1500 | 15 | Metformin | 40 | 17 | 44.3 (5.6) | 0.56 (0.2) | 1500 |
| Zhao Y 2014 | [89] | NR | 12 | Metformin+ Glipizide | 80 | 33 | 52.4 (9.7) | Newly diagnosed | Initial:750, 4^th^ w:1500 | Initial:15, 4^th^ w:30 | Metformin | 80 | 28 | 56.7 (9.5) | Newly diagnosed | Initial:750, 4^th^ w:1500 |
| Sun R 2013 | [90] | NR | 12 | Metformin+ Glipizide | 35 | 17 | 55.57 (8.62) | 5.15 (4.34) | 3pills/d | 3pills/d | Metformin | 30 | 16 | 54.82 (6.04) | 4.03 (3.68) | 3pills/d |
| Guo SB 2012 | [91] | NR | 12 | Metformin+ Glipizide | 61 | 26 | 33~62 | NR | 1500 | 15 | Metformin | 61 | 26 | 33~62 | NR | 1500 |
| Li P 2011 | [92] | NR | 12 | Metformin+ Glipizide | 100 | 52 | 55.79 (7.22) | 4.55 (4.98) | Initial:750, 4^th^ w:1500 | Initial:7.5, 4^th^ w:15 | Metformin | 50 | 32 | 56.16 (8.71) | 4.81 (4.25) | Initial:750, 4^th^ w:1500 |
| Ji Z 2011 | [93] | NR | 12 | Metformin+ Glipizide | 113 | NR | 18~70 | >1 | NR | NR | Metformin | 115 | NR | 18~70 | >1 | NR |
| Yao L 2010 | [94] | NR | 12 | Metformin+ Glipizide | 116 | NR | 30~75 | NR | Initial:750, 4^th^ w:1500 | Initial:7.5, 4^th^ w:15 | Metformin | 118 | NR | 30~75 | NR | Initial:750, 4^th^ w:1500 |
| Yao J 2008 | [95] | NR | 12 | Metformin+ Glipizide | 119 | 56 | 57.68 (8.93) | 5.46 (4.5) | Initial:750, 4^th^ w:1500 | Initial:7.5, 4^th^ w:15 | Metformin | 58 | 27 | 56.83 (10.02) | 5.21 (4.3) | Initial:750, 4^th^ w:1500 |
| Cui HM 2007a | [96] | Diet+exercise | 12 | Metformin+ Sulfonylurea | 34 | 15 | 51 (4) | NR | 750 | NR | Metformin | 31 | 15 | 52 (3) | NR | 750~1500 |
| Zhao HY 2012b | [72] | Diet+exercise | 12 | Metformin+ Repaglinide | 45 | 20 | 40.7 (18.3) | 5.4 (4.7) | Initial:750, adjust dose after 4w | Initial:1.5, adjust dose after 4w | Metformin | 45 | 20 | 40.7 (18.3) | 5.4 (4.7) | Initial:750, adjust dose after 4w |
| Kan LBNE 2016 | [97] | Diet+exercise | 12 | Metformin+ Repaglinide | 90 | 37 | 63.2 (9.2) | Newly diagnosed | Initial:750, later:1500 | Initial:1.5, later:3 | Metformin | 90 | 40 | 62.8 (8.7) | Newly diagnosed | Initial:750, later:1500 |
| Yang QY 2016 | [98] | Diet | 12 | Metformin+ Repaglinide | 35 | 16 | 64.8 (2.1) | 7.1 (2.3) | 1500 | 1.5 | Metformin | 35 | 15 | 65.1 (2.2) | 7.2 (2) | 1500 |
| Lei JJ 2016 | [99] | Diet+exercise | 12 | Metformin+ Repaglinide | 32 | 15 | 68.31 (3.67) | NR | 1000~2000 | 1.5~8 | Metformin | 32 | 14 | 68.74 (3.19) | NR | 1000~2000 |
| Yang J 2016 | [100] | Diet+exercise | 12 | Metformin+ Repaglinide | 35 | NR | 23.2 (2.5) | NR | 1500 | 1.5 | Metformin | 35 | NR | 24.3 (2.7) | NR | 1500 |
| Wang K 2016 | [101] | Diet+exercise | 12 | Metformin+ Repaglinide | 60 | 23 | 63.2 (3.9) | 6.9 (1.5) | 1500 | 1.5 | Metformin | 60 | 25 | 63.5 (3.6) | 6.4 (1.8) | 1500 |
| Li J 2016 | [102] | Diet+exercise | 12 | Metformin+ Repaglinide | 50 | 22 | 57.5 (6.8) | NR | 1000 | 3 | Metformin | 50 | 21 | 58.1 (5.7) | NR | 1000 |
| Hu ZY 2016 | [103] | Diet+exercise | 16 | Metformin+ Repaglinide | 72 | 28 | 53.12 (2.04) | Newly diagnosed | 1500 | 1.5 | Metformin | 72 | 32 | 52.29 (2.11) | Newly diagnosed | 1500 |
| Sun ZC 2015 | [104] | Diet+exercise | 12 | Metformin+ Repaglinide | 40 | 17 | 59.1 (5.1) | NR | 1500 | 0.5~4 | Metformin | 40 | 18 | 58.3 (4.9) | NR | 1500 |
| Yu YY 2015 | [105] | Diet+exercise | 12 | Metformin+ Repaglinide | 60 | 24 | 52.5 (10.2) | NR | 1500 | 1.5 | Metformin | 60 | 26 | 53.7 (9.5) | NR | 1500 |
| Liang F 2015 | [106] | Diet+exercise | 12 | Metformin+ Repaglinide | 44 | 19 | 53.8 (8.7) | NR | 750~1500 | Initial:1.5, max:3 | Metformin | 44 | 16 | 53.6 (8.3) | NR | 750~1500 |
| Sun X 2015 | [107] | Diet+exercise | 12 | Metformin+ Repaglinide | 35 | 15 | 54.1 (3.1) | Newly diagnosed | Initial:750, adjust dose after 2w | Initial:3, adjust dose after 2w | Metformin | 35 | 17 | 53.9 (3.2) | Newly diagnosed | Initial:750, adjust dose after 2w |
| Li B 2015 | [108] | Diet+exercise | 12 | Metformin+ Repaglinide | 30 | 10 | 49.36 (15.28) | NR | 3000 | 12 | Metformin | 30 | 18 | 51.32 (18.39) | NR | 3000 |
| Tan LL 2014 | [109] | Diet+exercise | 12 | Metformin+ Repaglinide | 33 | 14 | 46.5 (5.3) | NR | 750 | 3 | Metformin | 33 | 15 | 45.9 (5.1) | NR | 750 |
| Jiang GJ 2014 | [110] | Diet | 12 | Metformin+ Repaglinide | 40 | 15 | 62.5 (1) | 6 (1) | 750~1500 | 1.5~3 | Metformin | 40 | 14 | 62 (1) | 6.2 (0.8) | 750~1500 |
| Han XF 2014 | [111] | Diet+exercise | 16 | Metformin+ Repaglinide | 30 | 16 | 45.2 | 3~10 | 1500 | 3 | Metformin | 30 | 16 | 45.2 | 3~10 | 1500 |
| Huang JQ 2014 | [112] | Diet+exercise | 26 | Metformin+ Repaglinide | 30 | 14 | 35~68 | 0.5~8 | 1500 | 1.5~3 | Metformin | 30 | 14 | 35~68 | 0.5~8 | 1500 |
| He M 2013A | [113] | Diet+exercise | 12 | Metformin+ Repaglinide | 61 | 25 | 46.05 (10.26) | Newly diagnosed | 1500 | Initial:1.5, max:16 | Metformin | 61 | 24 | 45.68 (9.18) | Newly diagnosed | 1500 |
| He M 2013B | [114] | Diet+exercise | 12 | Metformin+ Repaglinide | 74 | 36 | 47.27 (8.72) | NR | 1500 | Initial:1.5, max:16 | Metformin | 74 | 36 | 47.27 (8.72) | NR | 1500 |
| Li L 2013 | [115] | NR | 16 | Metformin+ Repaglinide | 60 | 15 | 53.3 (10.2) | Newly diagnosed | 1500 | 1.5 | Metformin | 60 | 22 | 50.3 (8.3) | Newly diagnosed | 1500 |
| Xu SL 2013 | [116] | NR | 12 | Metformin+ Repaglinide | 280 | 93 | 38.7 | Newly diagnosed | 1500 | 1.5 | Metformin | 280 | 93 | 38.7 | Newly diagnosed | 1500 |
| Jin Y 2012 | [117] | NR | 12 | Metformin+ Repaglinide | 32 | 14 | 53.2 | 0.5~1.5 | 1500 | 3 | Metformin | 33 | 13 | 53.2 | 0.5~1.5 | 1500 |
| Xue F 2012 | [118] | Diet+exercise | 12 | Metformin+ Repaglinide | 34 | 18 | 40~72 | Newly diagnosed | 750~1500 | 1.5~3 | Metformin | 36 | 21 | 35~70 | Newly diagnosed | 750~1500 |
| Zhou YX 2012 | [119] | Diet+exercise | 12 | Metformin+ Repaglinide | 80 | 36 | 50.8 | <3 | 750~1500 | 1.5~3 | Metformin | 80 | 36 | 50.8 | <3 | 750~1500 |
| Chen EF 2012 | [120] | Diet+exercise | 12 | Metformin+ Repaglinide | 64 | 27 | 55.24 (9.68) | 1.45 (0.6) | 1500 | 3 | Metformin | 64 | 27 | 55.24 (9.68) | 1.45 (0.6) | 1500 |
| Zhu AQ 2012 | [121] | Diet+exercise | 12 | Metformin+ Repaglinide | 30 | 14 | 40~70 | 1~8 | 750 | 6 | Metformin | 30 | 14 | 40~70 | 1~8 | 750 |
| Jin SL 2011 | [122] | Diet+exercise | 12 | Metformin+ Repaglinide | 52 | 28 | 42.67 (8.52) | 0.81 (0.19) | 1500 | 1.5 | Metformin | 52 | 28 | 42.67 (8.52) | 0.81 (0.19) | 1500 |
| Chen YP 2010 | [123] | Diet+exercise | 12 | Metformin+ Repaglinide | 32 | 15 | 47.9 (13.5) | Newly diagnosed | 750~1500 | 1.5~3 | Metformin | 30 | 14 | 47.1 (11.9) | Newly diagnosed | 750~1500 |
| Yan GQ 2015 | [124] | NR | 12 | Metformin+ Repaglinide | 75 | 38 | 52.6 (7.2) | Newly diagnosed | Initial:500, max:2000 | Initial:0.5 | Metformin | 75 | 37 | 52.3 (6.8) | Newly diagnosed | Initial:500, max:2000 |
| Yan XM 2015 | [125] | NR | 12 | Metformin+ Repaglinide | 47 | 18 | 47.7 (5) | NR | 750~1500 | 1.5~3 | Metformin | 40 | 15 | 48.2 (5.3) | NR | 750~1500 |
| Shao JZ 2013 | [126] | NR | 12 | Metformin+ Nateglinide | 128 | 59 | 55.71 (2.35) | 3.3 (2.5) | 1500 | 180 | Metformin | 128 | 73 | 56.23 (3.02) | 4.1 (2.8) | 1500 |
| Yao L 2016 | [127] | Diet+exercise | 16 | Metformin+ Sitagliptin | 30 | 11 | 49.5 (9.2) | 1~12 | 1000 | 100 | Metformin | 30 | 11 | 49.5 (9.2) | 1~12 | 1000 |
| Pang XX 2016 | [128] | NR | 12 | Metformin+ Sitagliptin | 33 | 12 | 52.81 (9.54) | <0.75 | 1500 | 100 | Metformin | 32 | 10 | 53.51 (5.87) | <0.75 | 1500 |
| Han M 2016 | [129] | Diet+exercise | 12 | Metformin+ Sitagliptin | 34 | 14 | 50.62 (4.14) | 6.15 (1.23) | 1500 | 100 | Metformin | 34 | 13 | 50.43 (4.21) | 6.21 (1.29) | 1500 |
| Yu DY 2016 | [130] | Diet+exercise | 12 | Metformin+ Sitagliptin | 41 | 21 | 45.9 (2.5) | Newly diagnosed | 1500 | NR | Metformin | 41 | 20 | 46.2 (2.8) | Newly diagnosed | 1500 |
| Wang XL 2016 | [131] | NR | 12 | Metformin+ Sitagliptin | 32 | 14 | 44.5 (5.3) | NR | 1500 | 100 | Metformin | 32 | 15 | 44.8 (5.7) | NR | 1500 |
| Qiao YC 2016 | [132] | Diet+exercise | 12 | Metformin+ Sitagliptin | 32 | 15 | 47.6 (2.4) | 4.3 (1.2) | 1500 | 100 | Metformin | 32 | 15 | 47.6 (2.4) | 4.3 (1.2) | 1500 |
| Yang QM 2016 | [133] | Diet+exercise | 12 | Metformin+ Sitagliptin | 49 | 23 | 20~65 | Newly diagnosed | 1500 | 100 | Metformin | 49 | 23 | 20~65 | Newly diagnosed | 1500 |
| Wu XH 2016 | [134] | Diet+exercise | 12 | Metformin+ Sitagliptin | 45 | 19 | 45.5 | 3.5 | 1500 | 100 | Metformin | 45 | 19 | 45.5 | 3.5 | 1500 |
| Xu YL 2016 | [135] | Diet+exercise | 12 | Metformin+ Sitagliptin | 110 | NR | 45.68 (10.97) | NR | NR | NR | Metformin | 110 | NR | 48.37 (11.02) | NR | 1000 |
| Xia CW 2016 | [136] | NR | 12 | Metformin+ Sitagliptin | 45 | 18 | 58.7 (12.6) | 6.7 (2.3) | 1500 | 100 | Metformin | 45 | 16 | 59.3 (12.8) | 6.2 (2.4) | 1500 |
| Li LQ 2016 | [137] | Diet+exercise | 12 | Metformin+ Sitagliptin | 42 | 15 | 45.34 (3.13) | Newly diagnosed | 1500 | 100 | Metformin | 41 | 14 | 45.12 (3.43) | Newly diagnosed | 1500 |
| Hu GH 2016 | [138] | Diet+exercise | 12 | Metformin+ Sitagliptin | 48 | 22 | 53.2 (5.1) | Newly diagnosed | 500 | 100 | Metformin | 48 | 22 | 53.2 (5.1) | Newly diagnosed | 500 |
| Yan YH 2016 | [139] | Diet+exercise | 12 | Metformin+ Sitagliptin | 40 | 19 | 52.04 (2.16) | 4.95 (1.06) | 500~2000 | 100 | Metformin | 39 | 20 | 52.06 (2.14) | 4.97 (1.02) | 500~2000 |
| Li G 2016 | [140] | Diet+exercise | 12 | Metformin+ Sitagliptin | 44 | 20 | 56.21 (8.26) | NR | 500~2000 | 100 | Metformin | 44 | 19 | 57.05 (8.12) | NR | 500~2000 |
| Xiong M 2016 | [141] | Diet+exercise | 12 | Metformin+ Sitagliptin | 50 | 22 | 45.8 (3.2) | 0.48 (0.1) | Initial:1000, later:2250 | 100 | Metformin | 50 | 23 | 47.5 (3.4) | 0.49 (0.11) | Initial:1000, later:2250 |
| Zhang TT 2016 | [142] | Diet+exercise | 12 | Metformin+ Sitagliptin | 41 | 20 | 60.98 (3.14) | 6.14 (0.52) | 500 | 100 | Metformin | 41 | 21 | 61.13 (2.92) | 5.85 (0.12) | 500 |
| Qi BB 2016 | [143] | Diet+exercise | 24 | Metformin+ Sitagliptin | 30 | 12 | 40 (12) | Newly diagnosed | 1500 | 100 | Metformin | 30 | 16 | 42 (10) | Newly diagnosed | 1500 |
| Yang F 2016 | [144] | Diet+exercise | 26 | Metformin+ Sitagliptin | 45 | 20 | 56.58 (8.31) | 5.68 (1.35) | 1500 | 100 | Metformin | 45 | 19 | 56.62 (8.28) | 5.71 (1.37) | 1500 |
| Liu XL 2016 | [145] | Diet+exercise | 26 | Metformin+ Sitagliptin | 50 | 23 | 52.4 (6.2) | Newly diagnosed | 1500 | 100 | Metformin | 50 | 25 | 53.2 (6.5) | Newly diagnosed | 1500 |
| Jiang X 2016 | [146] | Diet+exercise | 52 | Metformin+ Sitagliptin | 30 | 14 | 43.7 (6.5) | 6.5 (0.8) | 1500 | 100 | Metformin | 30 | 11 | 44.6 (8.7) | 6.2 (0.7) | 1500 |
| Fu HY 2015 | [147] | Diet+exercise | 12 | Metformin+ Sitagliptin | 34 | 14 | 32~58 | 5.2 (4.3) | 1500 | 100 | Metformin | 34 | 14 | 32~58 | 5.2 (4.3) | 1500 |
| Huang YL 2015 | [148] | NR | 12 | Metformin+ Sitagliptin | 32 | 16 | 43.6 (6.4) | NR | 1500 | 100 | Metformin | 32 | 15 | 43.2 (6.7) | NR | 1500 |
| Yuan XJ 2015 | [149] | Diet+exercise | 12 | Metformin+ Sitagliptin | 50 | 22 | 56.5 | Newly diagnosed | 1500 | 100 | Metformin | 50 | 24 | 54.5 | Newly diagnosed | 1500 |
| Li Z 2015 | [150] | Diet+exercise | 12 | Metformin+ Sitagliptin | 31 | 13 | 47.9 (4.2) | NR | 1500 | 100 | Metformin | 31 | 13 | 47.9 (4.2) | NR | 1500 |
| Wu QP 2015 | [151] | NR | 12 | Metformin+ Sitagliptin | 35 | 14 | 51.6 (6.4) | 4.8 (1.1) | Initial:500, max:2000 | 100 | Metformin | 35 | 15 | 51.2 (6.3) | 4.3 (1) | Initial:500, max:2000 |
| Zhuo FT 2015 | [152] | Diet+exercise | 12 | Metformin+ Sitagliptin | 34 | 17 | 62.71 (12.93) | Newly diagnosed | 1500 | 100 | Metformin | 35 | 17 | 61.65 (12.55) | Newly diagnosed | 1500 |
| Zhang J 2015 | [153] | Diet+exercise | 12 | Metformin+ Sitagliptin | 50 | 22 | 49.23 (11.47) | 5.34 (1.42) | 1500 | 100 | Metformin | 48 | 22 | 49.23 (11.47) | 5.34 (1.42) | 1500 |
| Chen P 2015a | [154] | Diet+exercise | 12 | Metformin+ Sitagliptin | 30 | NR | NR | NR | 1500 | 100 | Metformin | 30 | NR | NR | NR | 1500 |
| Fan SH 2015 | [155] | Diet+exercise | 12 | Metformin+ Sitagliptin | 32 | 14 | 55.3 (10.8) | Newly diagnosed | 1500 | 100 | Metformin | 32 | 13 | 52.5 (9.6) | Newly diagnosed | 1500 |
| Zhang N 2015 | [156] | Diet+exercise | 12 | Metformin+ Sitagliptin | 62 | 21 | 54.2 (5.2) | 5.5 (0.9) | 1500 | 100 | Metformin | 62 | 22 | 54.6 (5.6) | 5.3 (0.7) | 1500 |
| Zhang HT 2015 | [157] | NR | 24 | Metformin+ Sitagliptin | 35 | 16 | 48.5 (10.8) | 0.6 (0.13) | 1500 | 100 | Metformin | 35 | 16 | 49.2 (11.2) | 0.59 (0.11) | 1500 |
| Wang KL 2014 | [158] | NR | 12 | Metformin+ Sitagliptin | 60 | 27 | 52 (11) | Newly diagnosed | 1500 | 100 | Metformin | 60 | 27 | 58 (8) | Newly diagnosed | 1500 |
| Yang H 2014 | [159] | Diet+exercise | 12 | Metformin+ Sitagliptin | 30 | 10 | 51.4 (5.15) | Newly diagnosed | 1500 | 100 | Metformin | 30 | 11 | 52.4 (6.33) | Newly diagnosed | 1500 |
| Liu SY 2014 | [160] | Diet+exercise | 12 | Metformin+ Sitagliptin | 48 | 23 | 42.1 | NR | 1000~1500 | 100 | Metformin | 48 | 23 | 42.1 | NR | 1000~1500 |
| Guo YF 2014 | [161] | Diet+exercise | 12 | Metformin+ Sitagliptin | 45 | 0 | 78 (5.5) | 18.5 (2.8) | 1500 | 100 | Metformin | 45 | 0 | 78 (5.5) | 18.5 (2.8) | 1500 |
| Huang BX 2014 | [162] | NR | 12 | Metformin+ Sitagliptin | 40 | 18 | 44.2 (5.1) | 0.38 (0.18) | 750 | 100 | Metformin | 40 | 18 | 44.2 (5.1) | 0.38 (0.18) | 750 |
| Qiu W 2014 | [163] | Diet+exercise | 12 | Metformin+ Sitagliptin | 40 | 17 | 52.34 (3.47) | 6.23 (1.69) | 1500 | 100 | Metformin | 40 | 15 | 52.34 (3.47) | 6.23 (1.69) | 1500 |
| Shi AJ 2014 | [164] | Diet+exercise | 12 | Metformin+ Sitagliptin | 56 | 26 | 55.8 (11.5) | 6.29 (1.26) | 500~2000 | 100 | Metformin | 55 | 27 | 56.1 (12) | 6.32 (1.31) | 500~2000 |
| Ye X 2014 | [165] | NR | 12 | Metformin+ Sitagliptin | 42 | 17 | 45.3 (5.7) | NR | 1000~2000 | 100~200 | Metformin | 42 | 18 | 45.5 (5.8) | NR | 1000~2000 |
| Wei ZX 2014 | [166] | NR | 12 | Metformin+ Sitagliptin | 30 | 15 | 54.6 (1.8) | 0.36 (0.08) | 1500 | 300 | Metformin | 30 | 18 | 55.3 (2.6) | 0.32 (0.13) | 1500 |
| Zhang LL 2013 | [167] | Diet+exercise | 12 | Metformin+ Sitagliptin | 57 | 20 | 54.9 (10.8) | 6.17 (1.34) | 1500 | 100 | Metformin | 56 | 23 | 55.8 (10.7) | 6.19 (1.35) | 1500 |
| Li WM 2013 | [168] | NR | 12 | Metformin+ Sitagliptin | 70 | 33 | 51.3 (8.38) | 6.9 | 1500 | 100 | Metformin | 70 | 35 | 52.1 (7.72) | 7 | 1500 |
| Pan YP 2013 | [169] | Diet+exercise | 12 | Metformin+ Sitagliptin | 40 | 19 | 62.2 (6.3) | 5.4 (2.1) | 500~2000 | 100 | Metformin | 39 | 19 | 62.2 (6.3) | 5.4 (2.1) | 500~2000 |
| Jiao XM 2013 | [170] | Diet+exercise | 12 | Metformin+ Sitagliptin | 30 | 13 | 46.4 (8.2) | Newly diagnosed | 1500 | 100 | Metformin | 30 | 15 | 43.3 (7.1) | Newly diagnosed | 1500 |
| Zhang HY 2013 | [171] | Diet+exercise | 12 | Metformin+ Sitagliptin | 48 | 23 | 52 (4.9) | Newly diagnosed | 750 | 100 | Metformin | 48 | 21 | 51.8 (5.2) | Newly diagnosed | 750 |
| Nie FR 2013 | [172] | NR | 16 | Metformin+ Sitagliptin | 80 | 37 | 58.7 (4.6) | 0.44 (0.13) | 1500 | 100 | Metformin | 80 | 36 | 58.3 (4.2) | 0.43 (0.12) | 1500 |
| Zhao XH 2012 | [173] | Diet+exercise | 12 | Metformin+ Sitagliptin | 30 | NR | 35~70 | NR | 1500 | 100 | Metformin | 30 | NR | 35~70 | NR | 1500 |
| Huang BX 2011 | [174] | Diet+exercise | 12 | Metformin+ Sitagliptin | 40 | 19 | 43 (5) | 0.25~0.5 | 750 | 100 | Metformin | 40 | 19 | 43 (5) | 0.25~0.5 | 750 |
| Yang WY 2012 | [175] | Diet+exercise | 24 | Metformin+ Sitagliptin | 191 | 105 | 54.1 (9) | 6.4 (4.4) | 1000~1700 | 100 | Metformin | 194 | 90 | 55.1 (9.8) | 7.3 (4.6) | 1000~1700 |
| Su DY 2016 | [176] | Diet+exercise | 12 | Metformin+ Sitagliptin | 52 | 23 | 49.19 (10.67) | 4.78 (0.71) | 1500 | 100 | Metformin | 52 | 23 | 49.19 (10.67) | 4.78 (0.71) | 1500 |
| Yang YP 2016 | [177] | NR | 12 | Metformin+ Saxagliptin | 71 | 36 | 59.6 (3.3) | 5.6 (1.9) | 500 | 5 | Metformin | 71 | 37 | 60.2 (3.7) | 5.5 (2.1) | 500 |
| Zhao JY 2016 | [178] | Diet+exercise | 12 | Metformin+ Saxagliptin | 50 | 22 | 64.72 (3.58) | NR | Initial:500, later:1500 | 5 | Metformin | 50 | 19 | 64.19 (3.33) | NR | Initial:500, later:1500 |
| Ding R 2016 | [179] | Diet+exercise | 12 | Metformin+ Saxagliptin | 30 | 10 | 48.73 (8.7) | 5.11 (1.29) | 1500 | 5 | Metformin | 30 | 12 | 50.2 (7.74) | 4.58 (1.3) | 1500 |
| Li M 2015A | [180] | Diet+exercise | 12 | Metformin+ Saxagliptin | 35 | 12 | 47 (11.4) | 0~10 | 1500 | 5 | Metformin | 35 | 12 | 47 (11.4) | 0~10 | 1500 |
| Wang SY 2015 | [181] | Diet+exercise | 12 | Metformin+ Saxagliptin | 53 | 30 | 66.5 (3.5) | Newly diagnosed | 1500 | 5 | Metformin | 52 | 32 | 68.7 (4.3) | Newly diagnosed | 500~2000 |
| Gao F 2015 | [182] | Diet+exercise | 12 | Metformin+ Saxagliptin | 64 | 36 | <75 | Newly diagnosed | Initial:500, max:2000 | 5 | Metformin | 58 | 33 | <75 | Newly diagnosed | Initial:500, max:2000 |
| Li J 2015 | [183] | Diet+exercise | 12 | Metformin+ Saxagliptin | 59 | 31 | 51.2 (6.6) | Newly diagnosed | 1500 | 5 | Metformin | 59 | 29 | 51.3 (6.5) | Newly diagnosed | 1500 |
| Liang M 2015 | [184] | Diet+exercise | 12 | Metformin+ Saxagliptin | 48 | 22 | 54.2 (1.7) | Newly diagnosed | 1500 | 5 | Metformin | 48 | 23 | 53.2 (1.5) | Newly diagnosed | 1500 |
| Feng J 2015 | [185] | NR | 12 | Metformin+ Saxagliptin | 48 | 10 | 79.02 (3.26) | 17 (4.23) | 1500 | 5 | Metformin | 48 | 12 | 78.02 (4.56) | 18 (4.06) | 1500 |
| Sun L 2015 | [186] | NR | 12 | Metformin+ Saxagliptin | 52 | 32 | 51.7 (3.9) | 4.7 (1.6) | 1500 | 5 | Metformin | 51 | 30 | 52.1 (4.1) | 5.2 (1.3) | 1500 |
| Geng XH 2015 | [187] | Diet+exercise | 12 | Metformin+ Saxagliptin | 42 | 13 | 54.6 (13.2) | 0.79 | 1500 | 5 | Metformin | 42 | 13 | 54.6 (13.2) | 0.79 | 1500 |
| Zhao SY 2015 | [188] | Diet+exercise | 12 | Metformin+ Saxagliptin | 46 | 18 | 68.1 (4.9) | NR | 1500 | 5 | Metformin | 46 | 20 | 66.7 (5.2) | NR | 1500 |
| Li XY 2015 | [189] | NR | 12 | Metformin+ Saxagliptin | 30 | 12 | 56.4 (7.5) | 2~11 | Initial:1000, max:2000 | 5 | Metformin | 30 | 13 | 55.6 (7.2) | 2~10 | Initial:1000, max:2000 |
| Xie WS 2015 | [190] | Diet | 24 | Metformin+ Saxagliptin | 40 | 18 | 46.7 (11.8) | Newly diagnosed | 1500 | 5 | Metformin | 40 | 19 | 48.5 (12.3) | Newly diagnosed | 1500 |
| Diao YL 2015 | [191] | NR | 24 | Metformin+ Saxagliptin | 47 | 19 | 55.9 (10.8) | 6.2 (2.1) | 1500 | 5 | Metformin | 48 | 20 | 52.3 (10.1) | 5.6 (2.4) | 1500 |
| Wan J 2015 | [192] | NR | 24 | Metformin+ Saxagliptin | 35 |  | 55.5 (16.38) | Newly diagnosed | 1000 | 5 | Metformin | 33 |  | 55.5 (16.38) | Newly diagnosed | 1000 |
| Sun Y 2014A | [193] | Diet+exercise | 12 | Metformin+ Saxagliptin | 44 | 20 | 56 (8) | Newly diagnosed | 1500 | 5 | Metformin | 40 | 19 | 52 (7) | Newly diagnosed | 1500 |
| Lu JE 2014 | [194] | Diet | 16 | Metformin+ Saxagliptin | 30 | 16 | 46 (12) | Newly diagnosed | 1500 | 5 | Metformin | 30 | 12 | 45 (13) | Newly diagnosed | 1500 |
| Dong L 2014 | [195] | Diet+exercise | 24 | Metformin+ Saxagliptin | 40 | 20 | 44 (12) | NR | 1500 | 5 | Metformin | 40 | 18 | 45 (10) | NR | 1500 |
| Sun Y 2014B | [196] | Diet+exercise | 12 | Metformin+ Saxagliptin | 30 | 13 | 56.1 (8.4) | Newly diagnosed | 1500 | 5 | Metformin | 30 | 12 | 52.4 (7.2) | Newly diagnosed | 1500 |
| Fang Y 2013 | [197] | Diet+exercise | 12 | Metformin+ Saxagliptin | 60 | NR | 46.5 | NR | Initial:1500, max:2000 | 5 | Metformin | 60 | NR | 46.5 | NR | Initial:1500, max:2000 |
| Li JJ 2013 | [198] | NR | 24 | Metformin+ Saxagliptin | 33 | 17 | 56.48 (9.13) | Newly diagnosed | 1000 | 5 | Metformin | 33 | 14 | 52.79 (11.72) | Newly diagnosed | 1000 |
| Wen Q 2013 | [199] | NR | 24 | Metformin+ Saxagliptin | 200 | NR | 47.2 (13.5) | 0.61 (0.21) | 1500 | 10 | Metformin | 200 | NR | 46.9 (12.4) | 0.59 (0.23) | 1500 |
| Li R 2016 | [200] | Diet+exercise | 12 | Metformin+ Vildagliptin | 50 | 21 | 46.03 (8.87) | Newly diagnosed | 1500 | 100 | Metformin | 50 | 22 | 45.73 (9.23) | Newly diagnosed | 1500 |
| Zhao XW 2016A | [201] | Diet+exercise | 24 | Metformin+ Vildagliptin | 50 | 23 | 48.8 (8.6) | NR | 2000 | 100 | Metformin | 50 | 24 | 49.1 (10.2) | NR | 2000 |
| Xin CH 2016 | [202] | NR | 24 | Metformin+ Vildagliptin | 50 | 24 | 46.2 (6.14) | 6.2 (2.14) | 1000 | 100 | Metformin | 50 | 24 | 46.2 (6.14) | 6.2 (2.14) | 2000 |
| Meng ZL 2016 | [203] | Diet+exercise | 12 | Metformin+ Vildagliptin | 40 | 37 | 43.7 (6.7) | NR | 1500 | 100 | Metformin | 40 | 36 | 43.8 (6.9) | NR | 1500 |
| Zheng ZP 2015 | [204] | Diet+exercise | 12 | Metformin+ Vildagliptin | 50 | 20 | 59.27 (10.75) | Newly diagnosed | 1500 | 100 | Metformin | 50 | 23 | 56.55 (10.04) | Newly diagnosed | 1500 |
| Shangguan HY 2015 | [205] | Diet+exercise | 24 | Metformin+ Vildagliptin | 50 | 23 | 40~75 | NR | 1000 | 100 | Metformin | 50 | 24 | 40~75 | NR | 2000 |
| Xin CH 2015 | [206] | Diet+exercise | 24 | Metformin+ Vildagliptin | 50 | NR | 40~70 | NR | 1500 | 150 | Metformin | 50 | NR | 40~70 | NR | 3000 |
| Xie LK 2015 | [207] | NR | 26 | Metformin+ Vildagliptin | 80 | 35 | 51.7 (7.6) | NR | Initial:1000, 2w later:2500 | 100 | Metformin | 80 | 34 | 52.1 (9.4) | NR | Initial:1000, 2w later:2500 |
| Liang CY 2015 | [208] | NR | 36 | Metformin+ Vildagliptin | 42 | 12 | 52.2 (6.4) | NR | 1000 | 100 | Metformin | 42 | 22 | 53.7 (7.5) | NR | 1000 |
| Gao XH 2015 | [209] | NR | 26 | Metformin+ Vildagliptin | 39 | 19 | 53.91 (13.77) | 6.49 (5.76) | 800~1000 | 80~100 | Metformin | 34 | 16 | 55.84 (13.72) | 6.27 (5.82) | 800~2000 |
| Yan GJ 2015 | [210] | Diet+exercise | 16 | Metformin+ Vildagliptin | 70 | 39 | 55.9 (6.29) | 6.58 (1.28) | 750 | 150 | Metformin | 70 | 41 | 55.84 (6.27) | 6.54 (1.26) | 750 |
| Jia XW 2015 | [211] | NR | 12 | Metformin+ Vildagliptin | 87 | 44 | 48.8 (8.3) | 0.57 (0.39) | 1000 | 100 | Metformin | 85 | 44 | 46.2 (8) | 0.56 (0.29) | 1000 |
| Li Y 2014 | [212] | Diet+exercise | 12 | Metformin+ Vildagliptin | 40 | NR | 55.5 (5.2) | Newly diagnosed | 1500 | 100 | Metformin | 40 | NR | 54.5 (4.8) | Newly diagnosed | 1500 |
| Hao YH 2014 | [213] | NR | 12 | Metformin+ Vildagliptin | 30 | 11 | 20~58 | Newly diagnosed | 1500 | 5 | Metformin | 30 | 11 | 20~58 | Newly diagnosed | 1500 |
| Peng L 2014 | [214] | NR | 24 | Metformin+ Vildagliptin | 52 | 22 | 22 (58.7) | 8.65 (2.32) | 1000 | 100 | Metformin | 50 | 10 | 20 (60.3) | 8.78 (3.45) | 1500 |
| Lu SM 2014 | [215] | Diet | 54 | Metformin+ Vildagliptin | 60 | 23 | 65.51 (8.73) | 0.71 (0.24) | 1500 | 100 | Metformin | 60 | 25 | 66.1 (9.31) | 0.69 (0.25) | 1500 |
| Zou Y 2014 | [216] | Diet+exercise | 24 | Metformin+ Vildagliptin | 45 | 20 | 41 (7.42) | NR | 1500 | 100 | Metformin | 45 | 17 | 42 (9.58) | NR | 1500~2000 |
| Yan ZD 2014 | [217] | NR | 24 | Metformin+ Vildagliptin | 60 | 21 | 53.8 (7.1) | NR | 1500 | 100 | Metformin | 60 | 23 | 52.4 (6.7) | NR | 1500 |
| Lai KB 2014 | [218] | Diet+exercise | 12 | Metformin+ Vildagliptin | 52 | 22 | 45.3 (3.6) | 5.3 (1.6) | 1500 | 100 | Metformin | 52 | 21 | 45.5 (3.2) | 5.4 (1.3) | 1500 |
| Cheng LY 2014 | [219] | Diet+exercise | 12 | Metformin+ Vildagliptin | 75 | 45 | 55.82 (6.21) | 7.33 (1.69) | 1000 | 100 | Metformin | 75 | 48 | 55.76 (6.2) | 7.24 (1.66) | 1000 |
| Chen XS 2014 | [220] | NR | 26 | Metformin+ Vildagliptin | 60 | 37 | 64.38 (2.09) | 7.29 (1.59) | 1500 | 100 | Metformin | 60 | 37 | 64.38 (2.09) | 7.29 (1.59) | 1500 |
| Liu XH 2013 | [221] | Diet+exercise | 24 | Metformin+ Vildagliptin | 75 | 34 | 51.5 (8.7) | NR | 1500 | 50 | Metformin | 75 | 31 | 51.1 (8.9) | NR | 1500 |
| Yan J 2013 | [222] | Diet+exercise | 24 | Metformin+ Vildagliptin | 39 | 20 | 54.1 (8.1) | NR | 1000 | 100 | Metformin | 34 | 18 | 57.8 (8.7) | NR | 2000 |
| Zhao TQ 2012 | [223] | Diet | 24 | Metformin+ Vildagliptin | 100 | NR | 45.8 (12.3) | 0.61 (0.11) | 1500 | 50 | Metformin | 100 | NR | 46.1 (11.31) | 0.56 (0.12) | 1500 |
| Ji LN 2016 | [224] | NR | 24 | Metformin+ Vildagliptin | 2501 | 1140 | 56.5 (10.6) | 4.3 (4.2) | 1000 | 100 | Metformin | 484 | 244 | 56.2 (10.8) | 4.1 (4.3) | 2000 |
| Su Y 2014 | [225] | NR | 24 | Metformin+ Vildagliptin | 294 | 135 | 47.61 (14.409) | NR | NR | 100 | Metformin | 292 | 134 | 48.67 (11.564) | NR | NR |
| Pan C 2012a | [226] | NR | 24 | Metformin+ Vildagliptin | 146 | 73 | 54.2 (9.62) | 4.92 (4.8) | ≥1500 | 100 | Metformin | 144 | 78 | 54.5 (9.68) | 5.15 (4.58) | ≥1500 |
| Pan C 2012b | [226] | NR | 24 | Metformin+ Vildagliptin | 148 | 82 | 53.7 (10) | 5.02 (4.42) | ≥1500 | 50 | Metformin | 144 | 78 | 54.5 (9.68) | 5.15 (4.58) | ≥1500 |
| Chen P 2015b | [154] | Diet+exercise | 12 | Metformin+ Linagliptin | 30 | NR | NR | NR | 1500 | 5 | Metformin | 30 | NR | NR | NR | 1500 |
| Zhao XW 2016B | [227] | Diet+exercise | 24 | Metformin+ Linagliptin | 50 | 24 | 49.1 (10.2) | NR | 2000 | 5 | Metformin | 50 | 23 | 48.8 (8.6) | NR | 2000 |
| Zheng FP 2016a | [228] | Diet+exercise | 12 | Metformin+ Linagliptin | 33 | 17 | 47 (3.1) | Newly diagnosed | 1000 | 5 | Metformin | 33 | 15 | 48.5 (2.9) | Newly diagnosed | 1000 |
| Zheng FP 2016b | [228] | Diet+exercise | 12 | Metformin+ Alogliptin | 33 | 14 | 49.3 (2.6) | Newly diagnosed | 1000 | 25 | Metformin | 33 | 15 | 48.5 (2.9) | Newly diagnosed | 1000 |
| Weng Y 2016 | [229] | Diet+exercise | 20 | Metformin+ Alogliptin | 50 | NR | NR | NR | 1500 | 25 | Metformin | 50 | NR | NR | NR | 1500 |
| Zhao YP 2016 | [230] | Diet+exercise | 24 | Metformin+ Alogliptin | 50 | 25 | 48.4 (9.2) | NR | 1000 | 50 | Metformin | 50 | 26 | 48.2 (11.3) | NR | 2000 |
| Dong QY 2016A | [231] | Diet+exercise | 24 | Metformin+ Alogliptin | 50 | NR | 46.8 (9.2) | NR | 1000 | 25 | Metformin | 50 | NR | 45.5 (9.6) | NR | 2000 |
| Dong QY 2016B | [232] | Diet+exercise | 24 | Metformin+ Alogliptin | 100 | NR | 48.8 (8.6) | NR | 1000 | 25 | Metformin | 100 | NR | 49.1 (10.2) | NR | 2000 |
| Wang L 2016 | [233] | NR | 26 | Metformin+ Alogliptin | 37 | 14 | 47.12 (11.56) | Newly diagnosed | 1500 | 25 | Metformin | 37 | 15 | 47.13 (11.21) | Newly diagnosed | 1500 |
| Tai H 2016 | [234] | Diet+exercise | 26 | Metformin+ Alogliptin | 44 | 17 | 53.84 (9.53) | 6.6 (6.11) | 1000 | 25 | Metformin | 37 | 15 | 53.41 (10.46) | 6.57 (6.19) | 2000 |
| Su Y 2014 | [235] | Diet | 12 | Metformin+ DPP4 | 147 | 68 | 47.46 (13.4) | 27.59 (16.75) | NR | 100 | Metformin | 138 | 63 | 48.52 (10.55) | 27.59 (16.75) | NR |
| Li CY 2016 | [236] | NR | 12 | Metformin+ Rosiglitazone | 34 | 12 | 57 (9) | NR | 1000 | 4 | Metformin | 34 | 14 | 57 (9) | NR | 1000 |
| Xie LH 2016 | [237] | Diet+exercise | 24 | Metformin+ Rosiglitazone | 80 | 27 | 56.8 (7.2) | Newly diagnosed | 1500 | 4 | Metformin | 80 | 30 | 55.3 (6.8) | Newly diagnosed | 1500 |
| Li JY 2014 | [238] | Diet+exercise | 16 | Metformin+ Rosiglitazone | 140 | 48 | 39~72 | NR | 750 | 8 | Metformin | 140 | 52 | 37~71 | NR | 750 |
| Lei CY 2014 | [239] | Diet+exercise | 12 | Metformin+ Rosiglitazone | 110 | 50 | 52.23 (5.34) | 5.05 (0.41) | 1500 | 8 | Metformin | 110 | 51 | 52.18 (5.33) | 5.02 (0.36) | 1500 |
| Yang XD 2013 | [240] | NR | 16 | Metformin+ Rosiglitazone | 75 | 34 | 53.46 (1.78) | <2 | 1500 | 4 | Metformin | 75 | 35 | 53.12 (1.75) | <2 | 1500 |
| Wang SQ 2013 | [241] | Diet+exercise | 16 | Metformin+ Rosiglitazone | 55 | 25 | 50.1 (7.2) | 1~10 | 1000 | 4 | Metformin | 40 | 18 | 50.1 (7.2) | 1~10 | 1000 |
| Yu CY 2013 | [242] | Diet+exercise | 16 | Metformin+ Rosiglitazone | 60 | 31 | 53.46 (1.58) | <2 | 1500 | 4 | Metformin | 60 | 30 | 54.41 (1.67) | <2 | 1500 |
| Gao Y 2013 | [243] | NR | 16 | Metformin+ Rosiglitazone | 45 | 24 | 53.4 (1.8) | <2 | 1500 | 4 | Metformin | 45 | 25 | 54.1 (1.7) | <2 | 1500 |
| Liu M 2012 | [244] | NR | 12 | Metformin+ Rosiglitazone | 40 | 9 | 63.99 (2.78) | NR | 1500 | 4 | Metformin | 40 | 6 | 63.77 (3.23) | NR | 1500 |
| Luo YJ 2012 | [245] | Diet+exercise | 12 | Metformin+ Rosiglitazone | 40 | NR | 48.6 (3.2) | 5.1 (2) | 750 | 8 | Metformin | 40 | NR | 48.6 (3.2) | 5.1 (2) | 750 |
| Wang SY 2012 | [246] | NR | 12 | Metformin+ Rosiglitazone | 75 | 39 | 63.76 (6.43) | 0.5~5 | Initial:750, later:2000 | Initial:4, 2w later:8 | Metformin | 75 | 39 | 63.76 (6.43) | 0.5~5 | Initial:750, later:2000 |
| Si JL 2012 | [247] | Diet+exercise | 12 | Metformin+ Rosiglitazone | 40 | 12 | 45 (3.3) | NR | 1500 | 4 | Metformin | 40 | 8 | 45 (3.1) | NR | 1500 |
| Liu WJ 2011 | [248] | Diet+exercise | 12 | Metformin+ Rosiglitazone | 49 | 26 | 45 (3.2) | 8.3 (4.4) | 1500 | 4 | Metformin | 49 | 27 | 45 (3.4) | 8.2 (4.3) | 1500 |
| Li YG 2011 | [249] | NR | 12 | Metformin+ Rosiglitazone | 30 | 19 | 32~65 | 0.5~1 | 500 | 4 | Metformin | 30 | 19 | 32~65 | 0.5~1 | 500 |
| Xie YQ 2010 | [250] | Diet+exercise | 26 | Metformin+ Rosiglitazone | 30 | 12 | 30~65 | Newly diagnosed | 1000 | 4 | Metformin | 30 | 8 | 30~65 | Newly diagnosed | 1500 |
| Qi MP 2010 | [251] | Diet+exercise | 12 | Metformin+ Rosiglitazone | 42 | 13 | 45 | Newly diagnosed | 2000 | 8 | Metformin | 39 | 13 | 45 | Newly diagnosed | 2000 |
| Li GH 2010 | [252] | Diet+exercise | 24 | Metformin+ Rosiglitazone | 60 | NR | 43 (6) | 7 (4) | 750 | 4 | Metformin | 60 | NR | 43 (6) | 7 (4) | 750 |
| Zhong FD 2009 | [253] | Diet+exercise | 12 | Metformin+ Rosiglitazone | 36 | 18 | 44.67 (9.73) | Newly diagnosed | 500 | 4 | Metformin | 36 | 19 | 46.39 (10.13) | Newly diagnosed | 500 |
| Xia ZX 2009 | [254] | Diet+exercise | 24 | Metformin+ Rosiglitazone | 60 | 29 | 58.5 | 9.4 | 1000 | 4 | Metformin | 60 | 33 | 59.6 | 10.2 | 1000 |
| Li Y 2009 | [255] | NR | 26 | Metformin+ Rosiglitazone | 50 | 22 | 33.9 (12.6) | Newly diagnosed | NR | NR | Metformin | 48 | 20 | 34.1 (13) | Newly diagnosed | NR |
| Han SH 2007 | [256] | Diet+exercise | 12 | Metformin+ Rosiglitazone | 43 | NR | 44 (5.5) | NR | 750 | 4 | Metformin | 43 | NR | 44 (5.5) | NR | 750~1500 |
| Wang SF 2006 | [257] | Diet+exercise | 52 | Metformin+ Rosiglitazone | 40 | 15 | 46 (9) | Newly diagnosed | 1000 | 4 | Metformin | 40 | 20 | 46 (9) | Newly diagnosed | 1500 |
| Zhang GQ 2004 | [258] | NR | 26 | Metformin+ Rosiglitazone | 40 | NR | 29~78 | 5~19 | NR | 4 | Metformin | 36 | NR | 29~78 | 5~19 | NR |
| Chen HM 2016 | [259] | NR | 12 | Metformin+ Pioglitazone | 30 | 13 | 53.8 (6.4) | Newly diagnosed | 1500 | 15 | Metformin | 30 | 13 | 54.1 (7.5) | Newly diagnosed | 1500 |
| Zhang CR 2016 | [260] | Diet+exercise | 12 | Metformin+ Pioglitazone | 55 | 24 | 63.6 (5.9) | 8.3 (5.7) | 1500 | 30 | Metformin | 55 | 23 | 63.7 (5.8) | 8.1 (5.6) | 1500 |
| Li SQ 2016 | [261] | NR | 12 | Metformin+ Pioglitazone | 56 | 28 | 54.3 (13.8) | 7.7 (3.5) | 1000 | 15 | Metformin | 56 | 29 | 53.7 (13.1) | 8.1 (3) | 1000 |
| Jin HM 2016 | [262] | Diet+exercise | 12 | Metformin+ Pioglitazone | 40 | 15 | 57.22 (5.56) | NR | 750 | 15 | Metformin | 40 | 13 | 57.47 (5.89) | NR | 750 |
| Zhu WH 2015 | [263] | NR | 12 | Metformin+ Pioglitazone | 32 | NR | 53.4 (1.6) | 6.2 (1.8) | 1500 | 15 | Metformin | 32 | NR | 53.4 (1.6) | 6.2 (1.8) | 1500 |
| Song RS 2015 | [264] | NR | 12 | Metformin+ Pioglitazone | 65 | 34 | 60.4 (5.8) | NR | 500 | 15 | Metformin | 65 | 32 | 59.7 (5.1) | NR | 500 |
| Wen L 2015 | [265] | Diet+exercise | 12 | Metformin+ Pioglitazone | 31 | 17 | 62.1 (3.8) | NR | 750 | 15 | Metformin | 29 | 15 | 62.5 (4.1) | NR | 1500 |
| Liu SH 2015 | [266] | Diet+exercise | 12 | Metformin+ Pioglitazone | 40 | 14 | 54.28 | <0.25 | 1500 | 15 | Metformin | 40 | 14 | 54.28 | <0.25 | 1500 |
| Chang C 2015 | [267] | Diet+exercise | 24 | Metformin+ Pioglitazone | 110 | 58 | 45~75 | Newly diagnosed | 500 | 15 | Metformin | 110 | 59 | 45~75 | Newly diagnosed | 500 |
| Zhang CH 2015 | [268] | NR | 12 | Metformin+ Pioglitazone | 40 | 16 | 46.4 (1.6) | 7.4 (1.5) | 1500 | 20 | Metformin | 40 | 17 | 46.3 (1.7) | 7.2 (1.3) | 1500 |
| Lu S 2015 | [269] | NR | 12 | Metformin+ Pioglitazone | 36 | 14 | 43~82 | 10 (1.28) | Initial:1000, later:2550 | 15 | Metformin | 36 | 16 | 42~84 | 10 (3.7) | Initial:1000, later:2550 |
| Zhou YQ 2015 | [270] | Diet+exercise | 12 | Metformin+ Pioglitazone | 65 | 31 | 56.4 (5.2) | 8.9 (3.1) | 1500 | 15 | Metformin | 65 | 30 | 56.4 (5.2) | 8.9 (3.1) | 1500 |
| Ren YL 2015 | [271] | NR | 16 | Metformin+ Pioglitazone | 35 | 13 | 54.7 (5.5) | 4.6 (1.4) | 1000 | 30 | Metformin | 35 | 14 | 54.3 (5.8) | 4.7 (1.2) | 1000 |
| Wang T 2015 | [272] | Diet+exercise | 26 | Metformin+ Pioglitazone | 77 | 37 | 55.3 (6.5) | NR | 750 | 30 | Metformin | 75 | 36 | 55.8 (7.1) | NR | 750 |
| Zhu XX 2015 | [273] | NR | 26 | Metformin+ Pioglitazone | 80 | 37 | 57.9 (3.5) | NR | 1500 | 20 | Metformin | 80 | 35 | 58.3 (3.3) | NR | 1500 |
| Yang HM 2015 | [274] | Diet+exercise | 26 | Metformin+ Pioglitazone | 30 | NR | 35~65 | Newly diagnosed | 750 | 30 | Metformin | 30 | NR | 35~65 | Newly diagnosed | 750 |
| Yang XL 2015 | [275] | Diet+exercise | 26 | Metformin+ Pioglitazone | 90 | 44 | 48 (5.2) | 6.2 (3.8) | 1000 | 30 | Metformin | 90 | 41 | 47.5 (5) | 6 (3.5) | 1500 |
| Yang QM 2015 | [276] | Diet+exercise | 26 | Metformin+ Pioglitazone | 50 | NR | NR | NR | 1000 | 30 | Metformin | 50 | NR | NR | NR | 2000 |
| Ma ZX 2015 | [277] | Diet+exercise | 26 | Metformin+ Pioglitazone | 70 | 23 | 55.6 (7.2) | 9.04 (1.52) | 750 | 30 | Metformin | 70 | 20 | 54.9 (6.6) | 8.96 (1.47) | 750 |
| Liu HY 2014 | [278] | NR | 12 | Metformin+ Pioglitazone | 30 | 14 | 56.4 (7.2) | 2~21 | 1500 | 15 | Metformin | 30 | 12 | 54.2 (6.9) | 2~21 | 1500 |
| Deng MX 2014 | [279] | Diet+exercise | 12 | Metformin+ Pioglitazone | 80 | 35 | 46 (3.6) | Newly diagnosed | Initial:750, max:2000 | 15 | Metformin | 80 | 38 | 47 (3.5) | Newly diagnosed | Initial:750, max:2000 |
| Shen CM 2014 | [280] | NR | 26 | Metformin+ Pioglitazone | 102 | 39 | 56.3 (9.9) | 5.5 (1.9) | 1500 | 20 | Metformin | 102 | 47 | 58.2 (10.4) | 5.3 (1.9) | 1500 |
| Li HJ 2014 | [281] | Diet+exercise | 26 | Metformin+ Pioglitazone | 36 | 15 | 72.7 (6.8) | NR | 750 | 30 | Metformin | 36 | 14 | 72.2 (6.5) | NR | 1000 |
| Du YJ 2014 | [282] | Diet+exercise | 26 | Metformin+ Pioglitazone | 72 | 29 | 54.32 (4.71) | NR | 750 | 30 | Metformin | 72 | 31 | 54.92 (4.81) | NR | 750 |
| Zhou JJ 2014 | [283] | Diet+exercise | 52 | Metformin+ Pioglitazone | 34 | NR | NR | NR | 1000 | 30 | Metformin | 34 | NR | NR | NR | 1000 |
| Wang H 2013 | [284] | Diet+exercise | 12 | Metformin+ Pioglitazone | 32 | 15 | 61.8 (4.9) | 5.3 (1.8) | 1500 | 30 | Metformin | 32 | 14 | 60.1 (5.3) | 6.1 (1.7) | 1500 |
| Zhong J 2013 | [285] | NR | 12 | Metformin+ Pioglitazone | 30 | 16 | 61.2 (7.1) | 12.1 (3.2) | 750 | 30 | Metformin | 30 | 13 | 61.2 (7.1) | 12.1 (3.2) | 750 |
| Li X 2013 | [286] | Diet+exercise | 12 | Metformin+ Pioglitazone | 59 | NR | 37~62 | 2.8 | 750 | 4 | Metformin | 58 | NR | 37~62 | 2.8 | 750 |
| Xiao XY 2013 | [287] | Diet+exercise | 12 | Metformin+ Pioglitazone | 48 | 22 | 46.7 (10.7) | 8.8 (3.6) | 750 | 15 | Metformin | 47 | 21 | 46.5 (10.7) | 8.7 (3.7) | 1500 |
| Yan T 2013 | [288] | NR | 12 | Metformin+ Pioglitazone | 39 | 24 | 49.9 (9.5) | Newly diagnosed | 1000 | 30 | Metformin | 29 | 13 | 53.5 (13.6) | Newly diagnosed | 1000 |
| Bi XS 2013 | [289] | NR | 16 | Metformin+ Pioglitazone | 60 | NR | NR | NR | 1500 | 25 | Metformin | 60 | NR | NR | NR | 1500 |
| Zhu YF 2012 | [290] | NR | 12 | Metformin+ Pioglitazone | 67 | 31 | 55.7 (5.5) | NR | 1500 | 30 | Metformin | 67 | 30 | 56.5 (4.8) | NR | 1500 |
| Yang YB 2012 | [291] | Diet+exercise | 12 | Metformin+ Pioglitazone | 42 | NR | 50.2 (3.4) | 0.5~11 | 1500 | 30 | Metformin | 42 | NR | 50.2 (3.4) | 0.5~11 | 1500 |
| Yang F 2012 | [292] | NR | 26 | Metformin+ Pioglitazone | 40 | 19 | 53.1 (12.4) | NR | 1500 | 30 | Metformin | 40 | 20 | 52.7 (13.2) | NR | 1500 |
| Jiang H 2012 | [293] | NR | 26 | Metformin+ Pioglitazone | 113 | NR | NR | NR | 1500 | 20 | Metformin | 114 | NR | NR | NR | 1500 |
| Shao P 2012 | [294] | NR | 12 | Metformin+ Pioglitazone | 34 | 19 | 39 (5.8) | NR | 750 | 15 | Metformin | 34 | 18 | 39 (5.8) | NR | 1500 |
| Yuan ML 2012 | [295] | Diet+exercise | 12 | Metformin+ Pioglitazone | 64 | 28 | 48.52 (8.45) | Newly diagnosed | 1000 | 30 | Metformin | 64 | 30 | 47.3 (8.92) | Newly diagnosed | 1000~1500 |
| Teng YP 2012B | [296] | Diet+exercise | 26 | Metformin+ Pioglitazone | 50 | 20 | 44 (6.4) | NR | 1000 | 15 | Metformin | 50 | 24 | 45 (7.8) | NR | 1000 |
| Du SZ 2011 | [297] | NR | 12 | Metformin+ Pioglitazone | 86 | NR | 56.7 (9.6) | NR | 750 | 30 | Metformin | 86 | NR | 56.7 (9.6) | NR | 1000 |
| Yang Y 2011 | [298] | Diet+exercise | 12 | Metformin+ Pioglitazone | 49 | 21 | 56.1 (8.9) | Newly diagnosed | 750~1500 | 30 | Metformin | 49 | 23 | 55.8 (9.3) | Newly diagnosed | 750~1500 |
| Guo HF 2011 | [299] | Diet+exercise | 26 | Metformin+ Pioglitazone | 120 | 58 | 54.2 | NR | 1500 | 30 | Metformin | 120 | 59 | 55.3 | NR | 1500 |
| Wang GH 2010 | [300] | Diet+exercise | 52 | Metformin+ Pioglitazone | 36 | 21 | 50 (12) | Newly diagnosed | 1000 | 15 | Metformin | 36 | 21 | 50 (12) | Newly diagnosed | 1500 |
| Hong YJ 2010 | [301] | NR | 26 | Metformin+ Pioglitazone | 51 | 25 | 52.1 (13.6) | NR | 1500 | 30 | Metformin | 51 | 22 | 53.7 (14.3) | NR | 1500 |
| Wang XP 2009 | [302] | Diet+exercise | 52 | Metformin+ Pioglitazone | 30 | 15 | 46 (9) | Newly diagnosed | 1500 | 4 | Metformin | 30 | 10 | 46 (9) | Newly diagnosed | 1500 |
| Deng JH 2009 | [303] | Diet+exercise | 12 | Metformin+ Pioglitazone | 40 | 23 | NR | 0.5~1 | 1500 | 30 | Metformin | 40 | 23 | NR | 0.5~1 | 1500 |
| Cao XJ 2009 | [304] | Diet+exercise | 12 | Metformin+ Pioglitazone | 39 | 16 | 20~70 | Newly diagnosed | 750 | 15 | Metformin | 37 | 16 | 20~70 | Newly diagnosed | 1500 |
| Wang QK 2007 | [305] | Diet+exercise | 26 | Metformin+ Pioglitazone | 39 | 16 | 35~56 | 0.5~2.5 | 750 | 30 | Metformin | 37 | 16 | 34~59 | 0.42~2.67 | 1500 |
| Wang LH 2010 | [306] | NR | 52 | Metformin+ Pioglitazone | 30 | 15 | 46 (9) | Newly diagnosed | 1500 | 4 | Metformin | 30 | 10 | 46 (9) | Newly diagnosed | 1500 |
| Zhang YM 2013 | [307] | NR | 12 | Metformin+ Pioglitazone | 50 | 23 | 49.6 (16.3) | Newly diagnosed | 750 | 15 | Metformin | 46 | 23 | 49.6 (16.3) | Newly diagnosed | 750 |
| Liu SJ 2015 | [308] | NR | 12 | Metformin+ Glargine Insulin | 50 | 28 | 51.01 (8.12) | 5.6 (1.89) | 1000 | Initial:0.2U/kg·d, adjust dose later | Metformin | 50 | 26 | 53.12 (8.11) | 5.68 (2.01) | 1000 |
| Zhang WH 2015 | [309] | Diet+exercise | 12 | Metformin+ Glargine Insulin | 54 | 23 | 51.92 (3.44) | Newly diagnosed | 1500 | Initial:0.2U/kg·d, adjust dose later | Metformin | 54 | 21 | 51.54 (3.51) | Newly diagnosed | 1500 |
| Ma L 2015 | [310] | NR | 12 | Metformin+ Glargine Insulin | 48 | 25 | 53.7 (3.8) | 6.6 (1.9) | 1000 | Initial:0.2U/kg·d, adjust dose later | Metformin | 48 | 26 | 54.3 (3.4) | 6.8 (2.1) | 1000 |
| He RY 2015 | [311] | Diet | 12 | Metformin+ Glargine Insulin | 39 | 19 | 39 (4.5) | NR | 1500 | 0.15U/kg·d | Metformin | 39 | 20 | 38 (4.7) | NR | 1500 |
| Li M 2015B | [312] | NR | 12 | Metformin+ Glargine Insulin | 47 | 20 | 48.2 (8.5) | 5.4 (1.2) | 1500 | Initial:10U/d, adjust dose later | Metformin | 47 | 18 | 47.6 (7.3) | 5.6 (1.5) | 1500 |
| He YF 2014 | [313] | Diet+exercise | 12 | Metformin+ Glargine Insulin | 40 | 13 | 67.5 (5.2) | 6.8 (1.8) | 1500 | Initial:10U/d, adjust dose later | Metformin | 40 | 15 | 68.4 (5.5) | 7.2 (2.2) | 1500 |
| Lv CY 2008 | [314] | NR | 12 | Metformin+ Glargine Insulin | 28 | 13 | 52.4 (6.88) | 10.4 (3.8) | 750 | 6~18u | Metformin | 32 | 15 | 53.6 (7.81) | 9.8 (5.9) | 750 |
| Yao JY 2013 | [315] | NR | 12 | Metformin+ Glargine Insulin | 58 | 25 | 39.4 (3) | 17.1 (5.3) | 500~1000 | Initial:0.2U/kg·d | Metformin | 55 | 27 | 40.7 (3.2) | 17.7 (5.6) | 500~1000 |
| Ma YM 2015 | [316] | Diet+exercise | 16 | Metformin+ Glargine Insulin | 33 | 16 | 59 (11) | NR | 1500 | Initial:0.2U/kg·d, adjust dose later | Metformin | 33 | 13 | 63 (10) | NR | 1500 |
| Li RL 2014 | [317] | Diet+exercise | 12 | Metformin+ Isophane Protamine Biosynthetic Human Insulin | 50 | 21 | 59.53 (10.23) | 8.52 (1.6) | 1500 | Initial:8IU, 2~3d, adjust dose later | Metformin | 50 | 20 | 59.53 (10.23) | 8.52 (1.6) | 1500 |
| Wang JQ 2014 | [318] | Diet+exercise | 12 | Metformin+ Biphasic Aspartate Insulin | 60 | 33 | 56.72 | NR | 750~1500 | 20u/d | Metformin | 60 | 31 | 56.78 | NR | 750~1500 |
| Wang DY 2011 | [319] | NR | 12 | Metformin+ Insulin Detemir | 60 | 27 | 58.3 | NR | Initial:500, max:2000 | Initial:0.15U/kg·d, adjust dose later | Metformin | 60 | 25 | 59.2 | NR | Initial:500, max:2000 |
| Sun B 2009 | [320] | Diet+exercise | 12 | Metformin+ Protamine Zinc Recombinant Human Insulin | 46 | 18 | 63.4 | Newly diagnosed | Initial:750, max:1500 | Adjust dose based on glucose | Metformin | 46 | 18 | 63.4 | Newly diagnosed | Initial:750, max:1500 |
| Cao YQ 2014 | [321] | Diet+exercise | 12 | Metformin+ Protamine Zinc Recombinant Human Insulin | 40 | 16 | 54.4 (9.8) | 8~17 | 1500 | Initial: morning-15U/d, night-10U/d, later: morning-40U/d, night-30U/d | Metformin | 40 | 18 | 56.6 (10.2) | 7~19 | 1500 |
| Wang GF 2013 | [322] | Diet+exercise | 12 | Metformin+ Insulin | 41 | 18 | 58.63 (10.8) | Newly diagnosed | NR | NR | Metformin | 41 | 19 | 57.65 (10.9) | Newly diagnosed | NR |
| Cui HM 2007b | [96] | Diet+exercise | 12 | Metformin+ Protamine Zinc Recombinant Human Insulin | 44 | 23 | 53 (3) | NR | 750 | 8~24U/d | Metformin | 31 | 15 | 52 (3) | NR | 750~1500 |
| Wu ZY 2016 | [323] | Diet+exercise | 12 | Metformin+ Insulin Aspart 30 | 60 | 25 | 67.3 (3.1) | Newly diagnosed | 1500 | 10~12IU/d, adjust dose later | Metformin | 60 | 34 | 69.1 (5.1) | Newly diagnosed | 1500 |
| Jiang LY 2015 | [324] | NR | 12 | Metformin+ Exenatide | 55 | 25 | 43 (8.6) | NR | 1500 | Initial:10μg/d, 2^nd^ m:20μg/d | Metformin | 55 | 25 | 43 (8.6) | NR | 1500 |
| Hu H 2015 | [325] | NR | 12 | Metformin+ Exenatide | 50 | 22 | 25~62 | 1~7 | 3pills/d | Initial:10μg/d, 2^nd^ m:20μg/d | Metformin | 50 | 20 | 31~68 | 2~7 | 3pills/d |
| Li BW 2015 | [326] | NR | 16 | Metformin+ Exenatide | 128 | 60 | 52.14 (5.23) | NR | 1000 | Initial:10μg/d, 2m later:20μg/d | Metformin | 128 | 57 | 53.07 (5.46) | NR | 1000 |
| Pan NN 2014 | [327] | Diet+exercise | 12 | Metformin+ Exenatide | 30 | 17 | 60~85 | 0.25 | 1500 | Initial:10μg/d, 2^nd^ m:20μg/d | Metformin | 30 | 17 | 60~85 | 0.25 | 1500 |
| Niu L 2013 | [328] | NR | 12 | Metformin+ Exenatide | 40 | 18 | 41.2 (7.3) | >1 | 1500 | Initial:10μg/d, 2^nd^ m:20μg/d | Metformin | 40 | 19 | 40.5 (7.5) | >1 | 1500 |
| Ma CH 2016b | [65] | Diet+exercise | 12 | Metformin+ Liraglutide | 30 | 13 | 45 (10) | 5 (3) | 1500 | Initial:0.6~1.2, max:1.8 | Metformin | 30 | 14 | 44 (10) | 5 (3) | 1500 |
| Yang QF 2016b | [67] | NR | 15 | Metformin+ Liraglutide | 140 | 65 | 58.12 (11.82) | NR | 2000 | 1.8 | Metformin | 70 | 34 | 57.39 (12.24) | NR | 2000 |
| Wang F 2016 | [329] | NR | 12 | Metformin+ Liraglutide | 40 | 18 | 47.85 (6.42) | NR | 2000 | 0.6 | Metformin | 40 | 19 | 48.06 (5.47) | NR | 2000 |
| Wang XC 2016 | [330] | NR | 12 | Metformin+ Liraglutide | 30 | 13 | 45 (10) | 5 (3) | 1500 | Initial:0.6, 1w later:1.2, maintain:1.8 | Metformin | 30 | 14 | 44 (10) | 5 (3) | 1500 |
| Song WR 2016 | [331] | NR | 12 | Metformin+ Liraglutide | 53 | 19 | 38.7 (4.9) | NR | 2000 | 0.6 | Metformin | 53 | 20 | 38.2 (5.1) | NR | 2000 |
| Wang CX 2016 | [332] | Diet+exercise | 24 | Metformin+ Liraglutide | 81 | 30 | 44.52 (9.37) | NR | 1500 | Initial:0.6, 1w later:1.2 | Metformin | 81 | 30 | 45.45 (7.62) | NR | 1500 |
| Fang ZD 2016 | [333] | Diet+exercise | 12 | Metformin+ Liraglutide | 30 | 15 | 43.6 (8.7) | 2.6 (1) | Initial:750, max:2000 | Initial:0.6, max:1.8 | Metformin | 30 | 15 | 44 (9.1) | 2.7 (1.2) | Initial:750, max:2000 |
| Zhao YL 2016 | [334] | Diet+exercise | 12 | Metformin+ Liraglutide | 80 | 36 | 50.1 (13.2) | 0.29 (0.13) | 1000 | Initial:0.6, adjust dose based on glucose | Metformin | 80 | 32 | 47.1 (8.9) | 0.32 (0.1) | 1000 |
| Wei DH 2016 | [335] | Diet | 16 | Metformin+ Liraglutide | 68 | 31 | 51.4 (6.3) | 6.2 (2.1) | 2000 | 0.6 | Metformin | 68 | 30 | 52.6 (5.8) | 5.8 (1.3) | 2000 |
| Gao HM 2016 | [336] | Education | 12 | Metformin+ Liraglutide | 43 | 18 | 55.4 (2.3) | 3.5 (0.8) | Initial:1000, 2^nd^ w:2000, adjust dose later | Initial:0.6, 2^nd^ w:1.2, max:1.8 | Metformin | 43 | 19 | 54.9 (3.5) | 3.9 (0.5) | Initial:1000, 2^nd^ w:2000, adjust dose later |
| Sun F 2016 | [337] | NR | 16 | Metformin+ Liraglutide | 49 | 23 | 64.87 (4.28) | 5.23 (1.01) | Initial:750, later:1500 | 0.6 | Metformin | 49 | 24 | 64.21 (4.42) | 5.34 (1.29) | Initial:750, later:1500 |
| Xue GF 2016 | [338] | NR | 16 | Metformin+ Liraglutide | 49 | 20 | 52.4 (5.2) | 5.7 (1.3) | 2000 | 0.6 | Metformin | 49 | 22 | 53.3 (5.8) | 6.1 (1.8) | 2000 |
| Yao YN 2015 | [339] | Diet+exercise | 24 | Metformin+ Liraglutide | 66 | 11 | 45.31 (10.35) | NR | 1500 | Initial:0.6, 1w later:1.2 | Metformin | 66 | 13 | 43.88 (12.66) | NR | 1500 |
| Liu CL 2015 | [340] | Diet+exercise | 12 | Metformin+ Liraglutide | 60 | 31 | 55.34 (9.58) | NR | 1500 | 0.6~1.2 | Metformin | 60 | 35 | 53.33 (10.31) | NR | 1500 |
| Zhao FL 2015 | [341] | NR | 16 | Metformin+ Liraglutide | 44 | 20 | 55.08 (8.21) | NR | 2000 | 0.6 | Metformin | 44 | 21 | 53.76 (6.76) | NR | 2000 |
| Wang HQ 2015 | [342] | Diet+exercise | 12 | Metformin+ Liraglutide | 30 | 14 | 64.78 (2.25) | 3.77 (1.43) | 1500 | 0.6~1.8 | Metformin | 30 | 13 | 64.85 (2.26) | 3.63 (1.53) | 1000 |
| Yu YZ 2015 | [343] | Education | 12 | Metformin+ Liraglutide | 50 | 22 | 45.8 (3.2) | 0.48 (0.1) | Initial:1000, later:2250 | 0.6~1.8 | Metformin | 50 | 23 | 47.5 (3.4) | 0.49 (0.11) | Initial:1000, later:2250 |
| He J 2014 | [344] | Diet+exercise | 12 | Metformin+ Liraglutide | 50 | 22 | 50.12 (3.56) | 5.01 (2.11) | Initial:1000, 1w later:2000 | Initial:0.6, 1w later:1.2, max:1.8 | Metformin | 50 | 22 | 50.12 (3.56) | 5.01 (2.11) | Initial:1000, 1w later:2000 |
| Tian SJ 2013 | [345] | Diet+exercise | 24 | Metformin+ Liraglutide | 60 | 32 | 41.4 (5.3) | 0.85 (0.425) | 3000 | Initial:0.6, 2w later:1.2 | Metformin | 60 | 24 | 42.6 (3.8) | 0.82 (0.11) | 3000 |
| Liang C 2012 | [346] | Education | 16 | Metformin+ Liraglutide | 68 | 31 | 51.4 (6.3) | 6.2 (2.1) | 2000 | 0.6 | Metformin | 68 | 30 | 52.6 (5.8) | 5.8 (1.3) | 2000 |

Data are Number or Mean (SD). d=day. w=week. m=month. y=year. NR=not reported. Diet+exercise=dietary intervention and/or exercise management.

**Table S4. Framework for estimation of absolute treatment effects of glucose-lowering treatments based on meta-analysis and indirect treatment comparison**

| **Data source** | **Relative treatment effect (Δ)** | | **Absolute treatment effect** | |
| --- | --- | --- | --- | --- |
|  | **Meta-analysis (Δ)** | **Indirect treatment comparison (Δ)** | **Meta-analysis** | **Indirect treatment comparison** |
| “Metformin vs placebo/lifestyle intervention” studies | **Treatment effects of first therapy** |  |  |  |
|  | — | — | Metformin | — |
| “A glucose-lowering drug added onto metformin vs metformin” studies | **Treatment effects of second therapy** |  |  |  |
|  | Δ (1 vs 0)  Metformin + Sulfonylurea vs Metformin | — | 1  Metformin + Sulfonylurea | — |
|  | Δ (2 vs 0)  Metformin + Thiazolidinedione vs Metformin | Δ (2 vs 1)  Metformin + Thiazolidinedione vs Metformin + Sulfonylurea | — | 2 = 1 + Δ (2 vs 1)  Metformin + Thiazolidinedione |
|  | Δ (3 vs 0)  Metformin + α-Glucosidase inhibitor vs Metformin | Δ (3 vs 1)  Metformin + α-Glucosidase inhibitor vs Metformin + Sulfonylurea | — | 3 = 1 + Δ (3 vs 1)  Metformin + α-Glucosidase inhibitor |
|  | Δ (4 vs 0)  Metformin + Glinide vs Metformin | Δ (4 vs 1)  Metformin + Glinide vs Metformin + Sulfonylurea | — | 4 = 1 + Δ (4 vs 1)  Metformin + Glinide |
|  | Δ (5 vs 0)  Metformin + DPP-4 inhibitor vs Metformin | Δ (5 vs 1)  Metformin + DPP-4 inhibitor vs Metformin + Sulfonylurea | — | 5 = 1 + Δ (5 vs 1)  Metformin + DPP-4 inhibitor |
|  | **Treatment effects of third therapy** |  |  |  |
|  | Δ (6 vs 0)  Metformin + Insulin vs Metformin | Δ (6 vs 7)  Metformin + Insulin vs Metformin + GLP-1 receptor agonist | — | 6 = 7 + Δ (6 vs 7)  Metformin + Insulin |
|  | Δ (7 vs 0)  Metformin + GLP-1 receptor agonist vs Metformin | — | 7  Metformin + GLP-1 receptor agonist | — |

Treatment effects included changes in HbA1c, TC, HDL-C, SBP, and BMI levels. BMI, body mass index. DPP-4, dipeptidyl peptidase 4. GLP-1, glucagon-like peptide 1. HbA1c, glycosylated hemoglobin Alc. HDL-C, high density lipoprotein-cholesterol. SBP, systolic blood pressure. TC, total cholesterol.

**Table S5. Retail prices of the glucose-lowering drugs collected from government medicine purchase platform**

| **Product** | **Dosage Form** | **Specification** | **Manufacturer** | **Approval Number** | **Retail price (¥)** |
| --- | --- | --- | --- | --- | --- |
| **Metformin** |  |  |  |  |  |
| Metformin hydrochloride tablets | Film-coated tablets | 0.25g*100 tablets/bottle | Tianjin Pacific Pharmaceutical Co., Ltd. | H12020797 | 4.8 |
| Metformin hydrochloride tablets | Film-coated tablets | 0.5g*20 tablets/box | Bristol-Myers Squibb | H20023370 | 23.13 |
| Metformin hydrochloride tablets | Film-coated tablets | 0.25g*48 tablets/bottle | SZYY Group Pharmaceutical Limited | H32021625 | 2.8 |
| Metformin hydrochloride tablets | Film-coated tablets | 0.25g*24 tablets/box | China Associate Pharmaceutical Co., Ltd. | H44024853 | 3.42 |
| Metformin hydrochloride tablets | Film-coated tablets | 0.25g*96 tablets/box | Disha Pharmaceutical Group | H20103615 | 16 |
| Metformin hydrochloride tablets | Film-coated tablets | 0.25g*48 tablets/bottle | Guangzhou Baiyushan Tianxin Pharmaceutical Co., Ltd. | H44023514 | 28.8 |
| Metformin hydrochloride tablets | Film-coated tablets | 0.25g*100 tablets/bottle | CR Double-Crane Pharmaceutical Co., Ltd. | H11020541 | 9.2 |
| Metformin hydrochloride tablets | Film-coated tablets | 0.25g*100 tablets/bottle | Tianjin Zhongxin Pharma-Tianjin Pharmaceutical Manuf Actowy | H12020587 | 5.5 |
| Metformin hydrochloride tablets | Film-coated tablets | 0.25g*48 tablets/box | JingFeng ZhiYao Group Co., Ltd. | H11021518 | 6.6 |
| Metformin hydrochloride tablets | Film-coated tablets | 0.85g*20 tablets/box | Bristol-Myers Squibb | H20023371 | 30.58 |
| Metformin hydrochloride tablets | Film-coated tablets | 0.25g*48 tablets/bottle | Qilu Pharmaceutical Co., Ltd. | H37020561 | 4.5 |
| Metformin hydrochloride sustained-release tablets | Sustained-release tablets | 0.5g*20 tablets/box | Chongqing Conquer Pharmaceutical Co., Ltd. | H20052243 | 43.34 |
| Metformin hydrochloride sustained-release tablets | Sustained-release tablets | 0.5g*28 tablets/box | Shanxi Huayuan Medicine Biotechnology Co., Ltd. | H20060815 | 33.6 |
| Metformin hydrochloride sustained-release tablets | Sustained-release tablets | 0.5g*30 tablets/box | Shandong Mingren Freda Pharmaceutical Co., Ltd. | H20052118 | 12.1 |
| Metformin hydrochloride sustained-release tablets | Sustained-release tablets | 0.25g*60 tablets/bottle | Fuzhou Chenxing Pharmaceutical Co., Ltd. | H20080432 | 16.01 |
| Metformin hydrochloride sustained-release tablets | Sustained-release tablets | 0.5g*30 tablets/box | Chongqing Kerui Nanhai Pharmaceutical Co., Ltd. | H20050349 | 15 |
| Metformin hydrochloride sustained-release tablets | Sustained-release tablets | 0.5g*30 tablets/box | Beijing Taiyang Pharmaceutical Co., Ltd. | H20090050 | 8.98 |
| Metformin hydrochloride sustained-release tablets | Sustained-release tablets | 0.5g*30 tablets/box | Shandong Qidu Pharmaceutical Co., Ltd. | H20050374 | 14.5 |
| Metformin hydrochloride sustained-release tablets | Sustained-release tablets | 0.5g*30 tablets/box | Beijing Wanhui Shuanghe Pharmaceutical Co., Ltd. | H20041986 | 16 |
| Metformin hydrochloride sustained-release tablets | Sustained-release tablets | 0.5g*28 tablets/box | Shandong Health Pharmaceutical Co., Ltd. | H20051187 | 17.85 |
| Metformin hydrochloride sustained-release tablets | Sustained-release tablets | 0.5g*10 tablets/box | Beijing Wanhui Shuanghe Pharmaceutical Co., Ltd. | H20041986 | 6 |
| Metformin hydrochloride sustained-release tablets | Sustained-release tablets | 0.5g*30 tablets/bottle | Youcare Pharmaceutical Group Co., Ltd. | H20051289 | 7.87 |
| Metformin hydrochloride sustained-release tablets | Sustained-release tablets | 0.5g*30 tablets/bottle | Chiatai Tianqing Group Co., Ltd. | H20031104 | 16.78 |
| Metformin hydrochloride sustained-release tablets | Sustained-release tablets | 0.5g*30 tablets/box | Qingdao Huanghai Pharmaceutical Co., Ltd. | H20040154 | 12.9 |
| Metformin hydrochloride sustained-release tablets | Sustained-release tablets | 0.5g*30 tablets/box | Germany Merck KGaA | J20120010 | 45.21 |
| Metformin hydrochloride sustained-release tablets | Sustained-release tablets | 0.5g*24 tablets/box | China Meheco Topfond Pharma Co., Ltd. | H20031225 | 13.5 |
| Metformin hydrochloride sustained-release capsules | Sustained-release capsules | 0.25g*48 pills/box | Hebei Ideal & Hightech Pharmaceutical Co., Ltd. | H20050373 | 18 |
| Metformin hydrochloride enteric-coated tablets | Enteric-coated tablets | 0.25g*100 tablets/bottle | Guizhou Shengjitang Pharmaceutical Co., Ltd. | H52020955 | 52.17 |
| Metformin hydrochloride enteric-coated tablets | Enteric-coated tablets | 0.25g*60 tablets/bottle | Guizhou Shengjitang Pharmaceutical Co., Ltd. | H52020955 | 31.3 |
| Metformin hydrochloride enteric-coated tablets | Enteric-coated tablets | 0.25g*24 tablets/box | Beijing Liling Hengtai Pharmaceutical Co., Ltd. | H11021560 | 9.17 |
| Metformin hydrochloride enteric-coated tablets | Enteric-coated tablets | 0.5g*30 tablets/bottle | Beijing Liling Hengtai Pharmaceutical Co., Ltd. | H20073157 | 14.7 |
| Metformin hydrochloride enteric-coated tablets | Enteric-coated tablets | 0.5g*60 tablets/bottle | Hebei Tiancheng Pharmaceutical Co., Ltd. | H20093711 | 19.2 |
| Metformin hydrochloride enteric-coated tablets | Enteric-coated tablets | 0.5g*30 tablets/bottle | Hebei Tiancheng Pharmaceutical Co., Ltd. | H20093711 | 9.6 |
| Metformin hydrochloride enteric-coated tablets | Enteric-coated tablets | 0.25g*100 tablets/bottle | Guizhou Tianan Pharmaceutical Co., Ltd. | H52020960 | 16.5 |
| Metformin hydrochloride enteric-coated tablets | Enteric-coated tablets | 0.25g*48 tablets/bottle | Beijing Liling Hengtai Pharmaceutical Co., Ltd. | H11021560 | 18.34 |
| Metformin hydrochloride enteric-coated tablets | Enteric-coated tablets | 0.5g*45 tablets/bottle | Guizhou Shengjitang Pharmaceutical Co., Ltd. | H20044070 | 39.13 |
| Metformin hydrochloride enteric-coated tablets | Enteric-coated tablets | 0.25g*48 tablets/bottle | Hebei Tiancheng Pharmaceutical Co., Ltd. | H20031134 | 7.68 |
| Metformin hydrochloride enteric-coated capsules | Enteric-coated capsules | 0.25g*48 pills/box | China Associate Pharmaceutical Co., Ltd. | H20094132 | 12.48 |
| Metformin hydrochloride enteric-coated capsules | Enteric-coated capsules | 0.5g*48 pills/box | Beijing Shengyong Pharmaceutical Co., Ltd. | H20058567 | 23.9 |
| Metformin hydrochloride enteric-coated capsules | Enteric-coated capsules | 0.25g*36 pills/box | Changchun Dazheng Pharmaceutical Technology Co., Ltd. | H20065996 | 8.7 |
| Metformin hydrochloride enteric-coated capsules | Enteric-coated capsules | 0.25g*72 pills/box | Changchun Dazheng Pharmaceutical Technology Co., Ltd. | H20065996 | 17 |
| Metformin hydrochloride enteric-coated capsules | Enteric-coated capsules | 0.25g*36 pills/box | Zhuhai Rundu Pharmaceutical Co., Ltd. | H20060728 | 14.43 |
| **Sulfonylurea** |  |  |  |  |  |
| **Glyburide** |  |  |  |  |  |
| Glyburide tablets | Plain tablets | 2.5mg*100 tablets/bottle | Tianjin Pacific Pharmaceutical Co., Ltd. | H12020790 | 1.8 |
| **Glimepiride** |  |  |  |  |  |
| Glimepiride tablets | Plain tablets | 2mg*12 tablets/box | Guizhou Shengjitang Pharmaceutical Co., Ltd. | H20010556 | 15.65 |
| Glimepiride tablets | Plain tablets | 1mg*30 tablets/box | Beijing Beilu Pharmaceutical Co., Ltd. | H20010567 | 21.29 |
| Glimepiride tablets | Plain tablets | 2mg*12 tablets/box | Jiangsu Wanbang Biochemical Pharmaceutical Group Co., Ltd. | H20010575 | 9.2 |
| Glimepiride tablets | Plain tablets | 2mg*30 tablets/bottle | Jiangsu Wanbang Biochemical Pharmaceutical Group Co., Ltd. | H20010575 | 32.53 |
| Glimepiride tablets | Plain tablets | 2mg*30 tablets/box | Chongqing Conquer Pharmaceutical Co., Ltd. | H20030800 | 41.63 |
| Glimepiride tablets | Plain tablets | 2mg*24 tablets/box | Guizhou Tianan Pharmaceutical Co., Ltd. | H20010561 | 30.24 |
| Glimepiride tablets | Plain tablets | 2mg*15 tablets/box | Sanofi (Beijing) Pharmaceutical Co., Ltd. | H20057672 | 64.31 |
| Glimepiride tablets | Plain tablets | 1mg*36 tablets/bottle | PKU Healthcare Corp., Ltd. | H20010563 | 26.22 |
| Glimepiride tablets | Plain tablets | 2mg*10 tablets/box | Yangtze River Pharmaceutical Group Guangzhou Hairui Pharmaceutical Co., Ltd. | H20073592 | 14.43 |
| Glimepiride tablets | Plain tablets | 2mg*24 tablets/box | Guizhou Shengjitang Pharmaceutical Co., Ltd. | H20010556 | 29.82 |
| Glimepiride tablets | Plain tablets | 2mg*30 tablets/box | Beijing Beilu Pharmaceutical Co., Ltd. | H20041838 | 40.2 |
| Glimepiride tablets | Plain tablets | 2mg*15 tablets/box | Beijing Beilu Pharmaceutical Co., Ltd. | H20041838 | 21 |
| Glimepiride tablets | Plain tablets | 1mg*20 tablets/box | Chongqing Conquer Pharmaceutical Co., Ltd. | H20010543 | 16.42 |
| Glimepiride tablets | Plain tablets | 1mg*24 tablets/box | Jiangsu Wanbang Biochemical Pharmaceutical Group Co., Ltd. | H20031079 | 13.85 |
| Glimepiride dispersible tablets | Dispersible tablets | 1mg*12 tablets/box | Shiyao Group Ouyi Pharmaceutical Co., Ltd. | H20100183 | 12.17 |
| Glimepiride dispersible tablets | Dispersible tablets | 2mg*12 tablets/box | Shiyao Group Ouyi Pharmaceutical Co., Ltd. | H20100182 | 19.96 |
| Glimepiride pills | Pills | 1mg*20 pills/bottle | Shandong New Times Pharmaceutical Co., Ltd. | H20041773 | 25.26 |
| **Gliclazide** |  |  |  |  |  |
| Gliclazide tablets (Ⅱ) | Plain tablets | 80mg*60 tablets/box | Guizhou Shengjitang Pharmaceutical Co., Ltd. | H20003279 | 78.26 |
| Gliclazide tablets (Ⅱ) | Plain tablets | 80mg*60 tablets/box | Tianjin Huajin Pharmaceutical Co., Ltd. | H10910053 | 64.69 |
| Gliclazide tablets (Ⅱ) | Plain tablets | 80mg*30 tablets/box | Suzhou Chung-HWA Chemical & Pharmaceutical Industrial Co., Ltd. | H20003146 | 15.9 |
| Gliclazide tablets (Ⅱ) | Plain tablets | 80mg*60 tablets/box | Shijiazhuang No. 4 Pharmaceutical Co., Ltd. | H20073781 | 21.65 |
| Gliclazide tablets | Plain tablets | 80mg*60 tablets/box | Shijiazhuang No. 4 Pharmaceutical Co., Ltd. | H13021831 | 6.68 |
| Gliclazide tablets | Plain tablets | 80mg*60 tablets/box | Shanghai Shyndec Pharmaceutical Co., Ltd. | H10910057 | 14.82 |
| Gliclazide tablets | Plain tablets | 80mg*60 tablets/box | Tianjin Zhongxin Pharma-Tianjin Pharmaceutical Manuf Actowy | H10910019 | 18.86 |
| Gliclazide tablets | Plain tablets | 80mg*60 tablets/box | Yabao Pharmaceutical Group Co., Ltd. | H20043145 | 23 |
| Gliclazide sustained-release tablets | Sustained-release tablets | 60mg*15 tablets/box | France Servier Pharmaceutical Factory | J20110047 | 38.79 |
| Gliclazide sustained-release tablets | Sustained-release tablets | 30mg*20 tablets/box | Shandong Lukang Pharmaceutical Group Saite Co., Ltd. | H20083477 | 16.6 |
| Gliclazide sustained-release tablets | Sustained-release tablets | 30mg*60 tablets/box | Tianjin Zhongxin Pharma-Tianjin Pnaemaceutical Manuf Actowy | H20065582 | 40 |
| Gliclazide sustained-release tablets | Sustained-release tablets | 30mg*30 tablets/box | Jiangsu Hansoh Pharmaceutical Group Co., Ltd. | H20059730 | 18.3 |
| Gliclazide sustained-release tablets | Sustained-release tablets | 30mg*40 tablets/box | Tianjin Huairen Pharmacy Co., Ltd. | H20063861 | 23.9 |
| Gliclazide sustained-release tablets | Sustained-release tablets | 30mg*30 tablets/box | Beijing Winsunny Harmony Co., Ltd. | H20066231 | 14.84 |
| Gliclazide sustained-release tablets | Sustained-release tablets | 30mg*30 tablets/box | Servier (Tianjin) Pharmaceutical Co., Ltd. | H20044694 | 44.19 |
| Gliclazide sustained-release capsules | Sustained-release capsules | 30mg*30 pills/box | Guilin Huaxin Pharmaceutical Co., Ltd. | H20090125 | 29.57 |
| Gliclazide sustained-release capsules | Sustained-release capsules | 30mg*30 pills/box | Hangzhou Guoguang Pharmaceutical Co., Ltd. | H20070261 | 29.57 |
| Gliclazide dispersible tablets | Dispersible tablets | 40mg*40 tablets/box | Nanchang Hongyi Pharmaceutical Co., Ltd. | H20050102 | 20 |
| **Glipizide** |  |  |  |  |  |
| Glipizide tablets | Film-coated tablets | 5mg*30 tablets/box | JingFeng ZhiYao Group Co., Ltd. | H11021516 | 26.8 |
| Glipizide tablets | Plain tablets | 5mg*36 tablets/bottle | Guizhou Shengjitang Pharmaceutical Co., Ltd. | H20003280 | 28.9 |
| Glipizide tablets | Plain tablets | 5mg*30 tablets/box | Guangdong Pi Di Pharmaceutical Co., Ltd. | H44021593 | 15 |
| Glipizide tablets | Plain tablets | 5mg*30 tablets/box | Tianjin Zhongtian Pharmaceutical Co., Ltd. | H20003148 | 6.7 |
| Glipizide tablets | Plain tablets | 5mg*30 tablets/box | Hainan Zambon Pharmaceutical Co., Ltd. | H10930076 | 13.5 |
| Glipizide tablets | Plain tablets | 5mg*48 tablets/box | Zhuhai United Laboratories International Holdings Limited Zhongshan Branch | H10983110 | 14.97 |
| Glipizide tablets | Plain tablets | 2.5mg*100 tablets/box | Disha Pharmaceutical Group | H37022995 | 21.8 |
| Glipizide tablets | Plain tablets | 5mg*30 tablets/box | Suicheng Pharmaceutical Co., Ltd. | H20065945 | 30 |
| Glipizide tablets | Plain tablets | 2.5mg*80 tablets/box | TIPR Pharmaceutical Co., Ltd. | H12020671 | 12 |
| Glipizide tablets | Plain tablets | 5mg*30 tablets/box | Shandong Lukang Pharmaceutical Group Saite Co., Ltd. | H20046471 | 5.6 |
| Glipizide tablets | Plain tablets | 5mg*40 tablets/box | Disha Pharmaceutical Group | H20013363 | 18.5 |
| Glipizide controlled release tablets | Controlled release tablets | 5mg*14 tablets/box | USA Pfizer Pharmaceuticals LLC | J20100129 | 30.35 |
| Glipizide controlled release tablets | Controlled release tablets | 5mg*48 tablets/box | Zibo Wanjie Pharmaceutical Co., Ltd. | H20046427 | 69.17 |
| Glipizide controlled release tablets | Controlled release tablets | 5mg*21 tablets/box | Beijing Honglin Pharmaceutical Co., Ltd. | H20084634 | 26.25 |
| Glipizide controlled release tablets | Controlled release tablets | 5mg*24 tablets/box | Zibo Wanjie Pharmaceutical Co., Ltd. | H20046427 | 32.31 |
| Glipizide controlled release tablets | Controlled release tablets | 5mg*14 tablets/box | Beijing Honglin Pharmaceutical Co., Ltd. | H20084634 | 19.8 |
| Glipizide controlled release tablets | Controlled release tablets | 5mg*12 tablets/box | Zibo Wanjie Pharmaceutical Co., Ltd. | H20046427 | 16.57 |
| Glipizide sustained-release tablets | Sustained-release tablets | 5mg*12 tablets/box | Yangtze River Pharmaceutical Group Co., Ltd. | H10970356 | 8.7 |
| Glipizide sustained-release tablets | Sustained-release tablets | 5mg*24 tablets/box | Yangtze River Pharmaceutical Group Co., Ltd. | H10970356 | 17.55 |
| Glipizide dispersible tablets | Dispersible tablets | 5mg*40 tablets/box | Shandong Huasu Pharmaceutical Co., Ltd. | H20031028 | 19 |
| **Thiazolidinedione** |  |  |  |  |  |
| **Rosiglitazone** |  |  |  |  |  |
| Rosiglitazone hydrochloride tablets | Film-coated tablets | 4mg*10 tablets/box | Zhejiang Hisun Pharmaceutical Co., Ltd. | H20080257 | 16.71 |
| Rosiglitazone hydrochloride tablets | Film-coated tablets | 4mg*18 tablets/box | Guizhou Shengjitang Pharmaceutical Co., Ltd. | H20052465 | 28.39 |
| Rosiglitazone hydrochloride tablets | Film-coated tablets | 4mg*6 tablets/box | Guizhou Shengjitang Pharmaceutical Co., Ltd. | H20052465 | 15.59 |
| Rosiglitazone sodium tablets | Plain tablets | 4mg*7 tablets/box | Chongqing Taiji Industry (Group) Co., Ltd. | H20041399 | 31.99 |
| Rosiglitazone Tartrate Tablets | Dispersible tablets | 4mg*12 tablets/box | Zhejiang Jingxin Pharmaceutical Co., Ltd. | H20080255 | 24.98 |
| **Pioglitazone** |  |  |  |  |  |
| Pioglitazone hydrochloride tablets | Film-coated tablets | 30mg*14 tablets/box | Jiangsu Deyuan Pharmaceutical Co., Ltd. | 20110048 | 33.5 |
| Pioglitazone hydrochloride tablets | Film-coated tablets | 15mg*21 tablets/box | Jiangsu Deyuan Pharmaceutical Co., Ltd. | H20110047 | 39.08 |
| Pioglitazone hydrochloride tablets | Film-coated tablets | 15mg*7 tablets/box | Jiangsu Deyuan Pharmaceutical Co., Ltd. | H20110047 | 13.48 |
| Pioglitazone hydrochloride tablets | Plain tablets | 30mg*7 tablets/box | Hangzhou Zhongmei Huadong Medicine Co., Ltd. | H20060664 | 21.99 |
| Pioglitazone hydrochloride tablets | Plain tablets | 15mg*7 tablets/box | Hangzhou Zhongmei Huadong Medicine Co., Ltd. | H20050500 | 13.48 |
| Pioglitazone hydrochloride tablets | Plain tablets | 15mg*7 tablets/box | Beijing Taiyang Pharmaceutical Co., Ltd. | H20040267 | 13.5 |
| Pioglitazone hydrochloride tablets | Plain tablets | 15mg*30 tablets/box | Chengdu Dikang Pharmaceutical Co., Ltd. | H20052156 | 31.47 |
| Pioglitazone hydrochloride tablets | Plain tablets | 15mg*28 tablets/box | CSPC Group Yuanda (Dalian) Pharmaceutical Co., Ltd. | H20052682 | 36.28 |
| Pioglitazone hydrochloride tablets | Plain tablets | 30mg*7 sheets/plate | Beijing Taiyang Pharmaceutical Co., Ltd. | H20063525 | 19.48 |
| Pioglitazone hydrochloride tablets | Plain tablets | 15mg*7 tablets/box | Takeda Pharmaceutical Company Limited, Osaka Plant | J20140082 | 42.5 |
| Pioglitazone hydrochloride capsules | Capsules | 30mg*10 pills/box | Sichuan Luye Pharmaceutical Co., Ltd. | H20050137 | 39.29 |
| Pioglitazone hydrochloride capsules | Capsules | 15mg*20 pills/box | Northeast Pharm Shenyang Shide Pharmaceutical Co., Ltd. | H20050838 | 25.1 |
| Pioglitazone hydrochloride capsules | Capsules | 15mg*6 pills/box | Shandong Zibo Xincat Pharmaceutical Co., Ltd. | H20060621 | 11.18 |
| Pioglitazone hydrochloride dispersible tablets | Dispersible tablets | 30mg*14 tablets/box | Guizhou Tianan Pharmaceutical Co., Ltd. | H20080282 | 32.37 |
| Pioglitazone hydrochloride dispersible tablets | Dispersible tablets | 30mg*7 tablets/box | Guizhou Tianan Pharmaceutical Co., Ltd. | H20080282 | 16.6 |
| Pioglitazone hydrochloride dispersible tablets | Dispersible tablets | 15mg*30 tablets/bottle | Jiangsu Wanbang Biochemical Pharmaceutical Group Co., Ltd. | H20060178 | 38.1 |
| **α-Glucosidase inhibitor** |  |  |  |  |  |
| **Acarbose** |  |  |  |  |  |
| Acarbose tablets | Plain tablets | 50mg*30 tablets/box | Bayer Health Care Co., Ltd. | H19990205 | 61.92 |
| Acarbose tablets | Plain tablets | 50mg*45 tablets/box | Hangzhou Zhongmei Huadong Medicine Co., Ltd. | H20020202 | 64.23 |
| Acarbose capsules | Capsules | 50mg*30 pills/box | Sichuan Luye Pharmaceutical Co., Ltd. | H20020391 | 41.51 |
| **Voglibose** |  |  |  |  |  |
| Voglibose tablets | Plain tablets | 0.2mg*30 tablets/box | Tianjin Takeda Pharmaceutical Co., Ltd. | H20010308 | 45.4 |
| Voglibose tablets | Plain tablets | 0.3mg*30 tablets/box | Suzhou Chung-HWA Chemical & Pharmaceutical Industrial Co., Ltd. | H20143287 | 54.5 |
| Voglibose tablets | Plain tablets | 0.2mg*30 tablets/box | Chiatai Qingchubao Pharmaceutical Co., Ltd. | H20123030 | 39.03 |
| Voglibose tablets | Plain tablets | 0.2mg*30 tablets/box | Nanjing Hicin Pharmaceutical Co., Ltd. | H20103291 | 33 |
| Voglibose tablets | Plain tablets | 0.2mg*30 tablets/box | Zhejiang Jingxin Pharmaceutical Co., Ltd. | H20094209 | 26.09 |
| Voglibose tablets | Plain tablets | 0.2mg*30 tablets/box | Suzhou Chung-HWA Chemical & Pharmaceutical Industrial Co., Ltd. | H20093758 | 26.6 |
| Voglibose tablets | Plain tablets | 0.2mg*20 tablets/box | Suzhou Chung-HWA Chemical & Pharmaceutical Industrial Co., Ltd. | H20093758 | 18 |
| Voglibose chewable tablets | Chewable tablets | 0.2mg*30 tablets/box | Hangzhou Zhongmei Huadong Medicine Co., Ltd. | H20090301 | 46.26 |
| Voglibose chewable tablets | Chewable tablets | 0.2mg*30 tablets/box | Beijing Taiyang Pharmaceutical Co., Ltd. | H20090045 | 41.59 |
| Voglibose capsules | Capsules | 0.2mg*50 pills/box | Yangtze River Pharmaceutical Group Co., Ltd. | H20090178 | 61.29 |
| Voglibose capsules | Capsules | 0.2mg*24 pills/box | Jiangsu Wanbang Biochemical Pharmaceutical Group Co., Ltd. | H20090115 | 20.28 |
| Voglibose capsules | Capsules | 0.2mg*10 pills/box | Yangtze River Pharmaceutical Group Co., Ltd. | H20090178 | 13 |
| Voglibose capsules | Capsules | 0.2mg*30 pills/box | Zhongfu Pharmaceutical Co., Ltd. | H20080655 | 37.5 |
| Voglibose capsules | Capsules | 0.1mg*30 pills/box | Cisen Pharmaceutical Co., Ltd. | H20070305 | 22.65 |
| Voglibose capsules | Capsules | 0.2mg*30 pills/box | Cisen Pharmaceutical Co., Ltd. | H20070306 | 34.5 |
| Voglibose dispersible tablets | Dispersible tablets | 0.2mg*30 tablets/box | Jiangsu Chenpai Pharmaceutical Group Co., Ltd. | H20061197 | 37.96 |
| **Miglitol** |  |  |  |  |  |
| Miglitol tablets | Film-coated tablets | 50mg*30 tablets/box | Zhejiang Pharmaceutical Co., Ltd. Xinchang Pharmaceutical Factory | H20074195 | 53.3 |
| Miglitol tablets | Plain tablets | 25mg*36 pills/box | Shandong New Times Pharmaceutical Co., Ltd. | H20113504 | 45.91 |
| Miglitol tablets | Plain tablets | 50mg*24 tablets/box | Shandong New Times Pharmaceutical Co., Ltd. | H20083446 | 44.52 |
| Miglitol tablets | Plain tablets | 50mg*30 tablets/box | Sichuan Weiao Pharmaceutical Co., Ltd. | H20045043 | 55.2 |
| **Glinide** |  |  |  |  |  |
| **Repaglinide** |  |  |  |  |  |
| Repaglinide tablets | Plain tablets | 0.5mg*30 tablets/box | Jiangsu Hansoh Pharmaceutical Group Co., Ltd. | H20000362 | 17.04 |
| Repaglinide tablets | Plain tablets | 0.5mg*60 tablets/box | Beijing Winsunny Harmony Co., Ltd. | H20133017 | 29.62 |
| Repaglinide tablets | Plain tablets | 2.0mg*30 tablets/box | Germany Boehringer Ingelheim Pharma GmbH & Co.KG | H20130021 | 66.57 |
| Repaglinide tablets | Plain tablets | 1.0mg*30 tablets/box | Germany Boehringer Ingelheim Pharma GmbH & Co.KG | H20130023 | 57.38 |
| Repaglinide tablets | Plain tablets | 1.0mg*45 tablets/box | Tianjin Kangrui Pharmaceutical Co., Ltd. | H20123055 | 44.1 |
| Repaglinide tablets | Plain tablets | 1.0mg*30 tablets/box | Beijing Winsunny Harmony Co., Ltd. | H20133037 | 32.71 |
| Repaglinide tablets | Plain tablets | 0.5mg*30 tablets/box | Beijing Winsunny Harmony Co., Ltd. | H20133017 | 18.3 |
| Repaglinide tablets | Plain tablets | 0.5mg*60 tablets/box | Beijing Beilu Pharmaceutical Co., Ltd. | H20113380 | 31.77 |
| Repaglinide tablets | Plain tablets | 1.0mg*60 tablets/box | Jiangsu Hansoh Pharmaceutical Group Co., Ltd. | H20103637 | 50.62 |
| Repaglinide tablets | Plain tablets | 1.0mg*30 tablets/box | Jiangsu Hansoh Pharmaceutical Group Co., Ltd. | H20103637 | 25.96 |
| Repaglinide tablets | Plain tablets | 0.5mg*60 tablets/box | Jiangsu Hansoh Pharmaceutical Group Co., Ltd. | H20000362 | 32.85 |
| **Nateglinide** |  |  |  |  |  |
| Nateglinide tablets | Film-coated tablets | 0.12g*30 tablets/box | Jiangsu Deyuan Pharmaceutical Co., Ltd. | H20123016 | 35.1 |
| Nateglinide tablets | Film-coated tablets | 0.12g*12 tablets/box | Beijing Novartis Pharmaceutical Co., Ltd. | H20030504 | 28.25 |
| Nateglinide tablets | Plain tablets | 0.12g*24 tablets/box | China Meheco Topfond Pharma Co., Ltd. | H20058909 | 36.05 |
| Nateglinide tablets | Plain tablets | 60mg*24 tablets/box | China Meheco Topfond Pharma Co., Ltd. | H20058910 | 19.22 |
| Nateglinide tablets | Plain tablets | 30mg*27 tablets/box | Yangtze River Pharmaceutical Group Nanjing Hailing Pharmaceutical Co., Ltd. | H20040955 | 12.82 |
| Nateglinide dispersible tablets | Dispersible tablets | 60mg*24 tablets/box | Shandong Luoxin Pharmaceutical Group Co., Ltd. | H20080808 | 24 |
| **DPP-4 inhibitor** |  |  |  |  |  |
| **Sitagliptin** |  |  |  |  |  |
| Sitagliptin phosphate tablets | Film-coated tablets | 0.1g*7 tablets/box | UK Merck Sharp & Dohme Limited | J20140095 | 52.96 |
| Sitagliptin phosphate tablets | Film-coated tablets | 0.1g*14 tablets/box | UK Merck Sharp & Dohme Limited | J20140095 | 103.89 |
| **Saxagliptin** |  |  |  |  |  |
| Saxagliptin tablets | Film-coated tablets | 5mg*7 tablets/box | USA AstraZeneca Pharmaceuticals | J20150066 | 56.23 |
| **Vildagliptin** |  |  |  |  |  |
| Vildagliptin tablets | Plain tablets | 50mg*14 tablets/box | Spain Novartis Farmaceutica S.A. | H20110358 | 59.5 |
| **Linagliptin** |  |  |  |  |  |
| Linagliptin tablets | Film-coated tablets | 5mg*7 tablets/box | USA West-Ward Columbus Inc. | J20130081 | 58.47 |
| **Alogliptin** |  |  |  |  |  |
| Alogliptin benzoate tablets | Film-coated tablets | 25mg*10 tablets/box | Takeda Pharmaceutical Company Limited, Osaka Plant | H20130548 | 84.9 |
| **GLP-1 receptor agonist** |  |  |  |  |  |
| **Exenatide** |  |  |  |  |  |
| Exenatide injection | Injection | 10μg (0.25mg/ml,2.4ml/injection) * 1 injection | USA Baxter Pharmaceutical Solutions LLC | H20140822 | 1542.28 |
| Exenatide injection | Injection | 5μg (0.25mg/ml,1.2ml/injection) * 1 injection | USA Baxter Pharmaceutical Solutions LLC | H20140821 | 1233 |
| **Liraglutide** |  |  |  |  |  |
| Liraglutide injection | Injection | 3ml:18mg*1 injection | Denmark Novo Nordisk A/S | J20110026 | 410 |

We accessed the platform (<http://210.73.89.76/ServiceSelect/GetServiceSelectList)> on May 5, 2018. DPP-4, dipeptidyl peptidase 4. GLP-1, glucagon-like peptide 1.

**Table S6. Treatment effects of glucose-lowering treatments and nonpharmacologic treatment in the scenario analyses**

| **Treatment effects of first therapy** | **Class 1** | | | |  |  |  |  |  |  |  |  |  |  |  |  |  |  |  |  |
| --- | --- | --- | --- | --- | --- | --- | --- | --- | --- | --- | --- | --- | --- | --- | --- | --- | --- | --- | --- | --- |
|  | **Metformin** | | **Nonpharmacologic treatment** | |  |  |  |  |  |  |  |  |  |  |  |  |  |  |  |  |
|  | **Mean** | **SE** | **Mean** | **SE** |  |  |  |  |  |  |  |  |  |  |  |  |  |  |  |  |
| HbA1c change, % | -1.57 | 0.22 | -0.55 | 0.19 |  |  |  |  |  |  |  |  |  |  |  |  |  |  |  |  |
| TC change, mg/dl | -30.5 | 6.3 | -15.44 | 2.07 |  |  |  |  |  |  |  |  |  |  |  |  |  |  |  |  |
| HDL-C change, mg/dl | 10.04 | 2.36 | 5.41 | 1.67 |  |  |  |  |  |  |  |  |  |  |  |  |  |  |  |  |
| SBP change, mmHg | -0.36 | 2.26 | -3.26 | 2.05 |  |  |  |  |  |  |  |  |  |  |  |  |  |  |  |  |
| Weight change, kg | -7.51 | 1.09 | -3.51 | 1.25 |  |  |  |  |  |  |  |  |  |  |  |  |  |  |  |  |
| Symptomatic hypoglycemia, % | 1.81 | 1.04 | 0 | 0 |  |  |  |  |  |  |  |  |  |  |  |  |  |  |  |  |
| Severe hypoglycemia, % | 0.04 | 0.02 | 0 | 0 |  |  |  |  |  |  |  |  |  |  |  |  |  |  |  |  |
| Gastrointestinal reaction, % | 2.15 | 1.06 | 0 | 0 |  |  |  |  |  |  |  |  |  |  |  |  |  |  |  |  |
| **Treatment effects of second therapy** | **Class 2** | | | | **Class 3** | | | | **Class 4** | | | | **Class 5** | | | | **Class 6** | | | |
|  | **Metformin + Sulfonylurea** | | **Nonpharmacologic treatment** | | **Metformin + Thiazolidinedione** | | **Nonpharmacologic treatment** | | **Metformin + α-Glucosidase inhibitor** | | **Nonpharmacologic treatment** | | **Metformin + Glinide** | | **Nonpharmacologic treatment** | | **Metformin + DPP-4 inhibitor** | | **Nonpharmacologic treatment** | |
|  | **Mean** | **SE** | **Mean** | **SE** | **Mean** | **SE** | **Mean** | **SE** | **Mean** | **SE** | **Mean** | **SE** | **Mean** | **SE** | **Mean** | **SE** | **Mean** | **SE** | **Mean** | **SE** |
| HbA1c change, % | -2.61 | 0.18 | -0.61 | 0.37 | -2.13 | 0.13 | -0.34 | 0.35 | -2.13 | 0.26 | -0.33 | 0.45 | -2.7 | 0.2 | -0.67 | 0.4 | -2.02 | 0.08 | -0.11 | 0.33 |
| TC change, mg/dl | -6.56 | 2.36 | 13.13 | 7.16 | -32.43 | 4.43 | -10.42 | 8.3 | -72.59 | 14.28 | -27.8 | 19.37 | -11.58 | 7.78 | 4.25 | 10.59 | -39 | 5.12 | -1.54 | 9.05 |
| HDL-C change, mg/dl | -1.16 | 3.25 | -5.79 | 4.1 | 5.02 | 5.71 | -6.18 | 7.37 | 18.53 | 6.5 | 1.16 | 7.83 | -3.86 | 3.35 | -5.02 | 6.5 | 8.88 | 2.56 | 0.39 | 3.64 |
| SBP change, mmHg | -3.8 | 1.17 | -5.58 | 3.67 | -6.18 | 1.63 | -7.12 | 4.14 | -8.9 | 1.63 | -10.8 | 4.11 | — | — | — | — | -4.12 | 1.26 | -3.48 | 3.62 |
| Weight change, kg | -0.95 | 1.21 | 2.7 | 2.37 | -3.84 | 1.53 | 0.27 | 2.74 | -3.51 | 2.15 | 1.88 | 3.08 | -7.9 | 0.92 | 2.86 | 2.28 | -6.83 | 0.74 | 0 | 2.16 |
| Symptomatic hypoglycemia, % | 4.46 | 0.57 | 0 | 2.08 | 0.74 | 0.21 | 0 | 2.01 | 2.35 | 0.52 | 2.35 | 2.07 | 2.93 | 0.56 | 2.93 | 2.08 | 1.16 | 0.13 | 0.18 | 2 |
| Severe hypoglycemia, % | 0.09 | 0.01 | 0 | 0.04 | 0.02 | 0 | 0 | 0.04 | 0.05 | 0.01 | 0.05 | 0.04 | 0.06 | 0.01 | 0.06 | 0.04 | 0.02 | 0 | 0 | 0.04 |
| Gastrointestinal reaction, % | 4.62 | 0.59 | 6.62 | 2.12 | 2.94 | 0.34 | 1.94 | 1.57 | 7.29 | 0.88 | 4.29 | 2.22 | 5.89 | 0.98 | 4.89 | 2.26 | 4.73 | 0.27 | 4.73 | 1.55 |
| **Treatment effects of third therapy** | **Class 7** | | | | **Class 8** | | | |  |  |  |  |  |  |  |  |  |  |  |  |
|  | **Metformin + Insulin** | | **Nonpharmacologic treatment** | | **Metformin + GLP-1 receptor agonist** | | **Nonpharmacologic treatment** | |  |  |  |  |  |  |  |  |  |  |  |  |
|  | **Mean** | **SE** | **Mean** | **SE** | **Mean** | **SE** | **Mean** | **SE** |  |  |  |  |  |  |  |  |  |  |  |  |
| HbA1c change, % | -2.92 | 0.27 | -0.88 | 0.44 | -2.89 | 0.2 | -0.47 | 0.4 |  |  |  |  |  |  |  |  |  |  |  |  |
| TC change, mg/dl | -87.26 | 4.33 | -27.8 | 9.48 | -55.6 | 7.88 | -10.04 | 12.4 |  |  |  |  |  |  |  |  |  |  |  |  |
| HDL-C change, mg/dl | — | — | — | — | 0 | 5.71 | -4.63 | 7.37 |  |  |  |  |  |  |  |  |  |  |  |  |
| SBP change, mmHg | — | — | — | — | -7.38 | 1.62 | -4.9 | 3.75 |  |  |  |  |  |  |  |  |  |  |  |  |
| Weight change, kg | -7.6 | 3.48 | -2.83 | 4.7 | -7.54 | 0.92 | 0.54 | 2.26 |  |  |  |  |  |  |  |  |  |  |  |  |
| Symptomatic hypoglycemia, % | 5 | 1.04 | 5.98 | 3.65 | 3.47 | 0.58 | 4.45 | 2.57 |  |  |  |  |  |  |  |  |  |  |  |  |
| Severe hypoglycemia, % | 0.1 | 0.02 | 0.12 | 0.07 | 0.07 | 0.01 | 0.09 | 0.05 |  |  |  |  |  |  |  |  |  |  |  |  |
| Gastrointestinal reaction, % | 4.18 | 0.96 | 0.18 | 2.26 | 6.78 | 0.98 | 7.78 | 2.26 |  |  |  |  |  |  |  |  |  |  |  |  |

HbA1c, glycosylated hemoglobin Alc. TC, total cholesterol. HDL-C, high density lipoprotein-cholesterol. LDL-C, Low density lipoprotein-cholesterol. SBP, systolic blood pressure. BMI, body mass index. DPP-4, dipeptidyl peptidase-4. GLP-1, glucagon-like peptide-1.

**Table S7. Base-case results: cost-effectiveness of ten pharmacologic combination strategies and strategy ranking process based on the ICERs (per patient)**

| **Rank** | **Optimal** | **Treatment Strategy** | **Cost, ¥** | **QALYs** | **ICERs compared to next lowest cost** | | | | |
| --- | --- | --- | --- | --- | --- | --- | --- | --- | --- |
|  |  |  |  |  | **step 1** | **step 2** | **step 3** | **step 4** |  |
|  |  | **Strategy 1** | 52,923 | 13.965 | --- | --- | --- | --- |  |
| 1 | ☆ | **Strategy 7** | 55,729 | 14.085 | 23,288 | 23,288 | 23,288 | **23,288** |  |
|  |  | **Strategy 3** | 56,374 | 13.978 | SD:  rule out |  |  |  |  |
|  |  | **Strategy 5** | 60,741 | 14.019 | 104,876 | SD:  rule out |  |  |  |
|  |  | **Strategy 9** | 69,467 | 14.051 | 277,673 | 277,673 | SD:  rule out |  |  |
|  |  | **Strategy 2** | 81,569 | 13.997 | SD:  rule out |  |  |  |  |
|  |  | **Strategy 4** | 85,095 | 14.011 | 264,829 | SD:  rule out |  |  |  |
|  |  | **Strategy 8** | 85,142 | 14.117 | 438 | 438 | 234,731 | 917,312 |  |
|  |  | **Strategy 6** | 89,690 | 14.053 | SD:  rule out |  |  |  |  |
|  |  | **Strategy 10** | 98,597 | 14.084 | 287,895 | SD:  rule out |  |  |  |
| **Rank** | **Optimal** | **Treatment Strategy** | **Cost, ¥** | **QALYs** | **ICERs compared to next lowest cost** | | | | |
|  |  |  |  |  | **step 1** | **step 2** | **step 3** | **step 4** |  |
|  |  | **Strategy 1** | 52,923 | 13.965 | --- | --- | --- | --- |  |
|  |  | **Strategy 3** | 56,374 | 13.978 | 271,216 | 271,216 | ED:  rule out |  |  |
| 2 | ☆ | **Strategy 5** | 60,741 | 14.019 | 104,876 | 104,876 | 104,876 | **143,811** |  |
|  |  | **Strategy 9** | 69,467 | 14.051 | 277,673 | 277,673 | ED:  rule out |  |  |
|  |  | **Strategy 2** | 81,569 | 13.997 | SD:  rule out |  |  |  |  |
|  |  | **Strategy 4** | 85,095 | 14.011 | 264,829 | SD:  rule out |  |  |  |
|  |  | **Strategy 8** | 85,142 | 14.117 | 438 | 438 | 234,731 | 248,473 |  |
|  |  | **Strategy 6** | 89,690 | 14.053 | SD:  rule out |  |  |  |  |
|  |  | **Strategy 10** | 98,597 | 14.084 | 287,895 | SD:  rule out |  |  |  |
| **Rank** | **Optimal** | **Treatment Strategy** | **Cost, ¥** | **QALYs** | **ICERs compared to next lowest cost** | | | | |
|  |  |  |  |  | **step 1** | **step 2** | **step 3** | **step 4** |  |
|  |  | **Strategy 1** | 52,923 | 13.965 | --- | --- | --- | --- |  |
|  |  | **Strategy 3** | 56,374 | 13.978 | 271,216 | 271,216 | ED:  rule out |  |  |
| 3 | ☆ | **Strategy 9** | 69,467 | 14.051 | 179,199 | 179,199 | 179,199 | **192,848** |  |
|  |  | **Strategy 2** | 81,569 | 13.997 | SD:  rule out |  |  |  |  |
|  |  | **Strategy 4** | 85,095 | 14.011 | 264,829 | SD:  rule out |  |  |  |
|  |  | **Strategy 8** | 85,142 | 14.117 | 438 | 438 | 234,731 | 234,731 |  |
|  |  | **Strategy 6** | 89,690 | 14.053 | SD:  rule out |  |  |  |  |
|  |  | **Strategy 10** | 98,597 | 14.084 | 287,895 | SD:  rule out |  |  |  |
| **Rank** | **Optimal** | **Treatment Strategy** | **Cost, ¥** | **QALYs** | **ICERs compared to next lowest cost** | | | | |
|  |  |  |  |  | **step 1** | **step 2** | **step 3** | **step 4** | **step 5** |
|  |  | **Strategy 1** | 52,923 | 13.965 | --- | --- | --- | --- |  |
|  |  | **Strategy 3** | 56,374 | 13.978 | 271,216 | 271,216 | 271,216 | ED:  rule out |  |
|  |  | **Strategy 2** | 81,569 | 13.997 | 1,279,166 | 1,279,166 | ED:  rule out |  |  |
|  |  | **Strategy 4** | 85,095 | 14.011 | 264,829 | 264,829 | ED:  rule out |  |  |
| 4 | ☆ | **Strategy 8** | 85,142 | 14.117 | 438 | 438 | 438 | 205,718 | **211,180** |
|  |  | **Strategy 6** | 89,690 | 14.053 | SD:  rule out |  |  |  |  |
|  |  | **Strategy 10** | 98,597 | 14.084 | 287,895 | SD:  rule out |  |  |  |
| **Rank** | **Optimal** | **Treatment Strategy** | **Cost, ¥** | **QALYs** | **ICERs compared to next lowest cost** | | | | |
|  |  |  |  |  | **step 1** | **step 2** | **step 3** |  |  |
| 5 | ☆ | **Strategy 1** | 52,923 | 13.965 | --- | --- | **---** |  |  |
|  |  | **Strategy 3** | 56,374 | 13.978 | 271,216 | 271,216 | 271,216 |  |  |
|  |  | **Strategy 2** | 81,569 | 13.997 | ED:  rule out |  |  |  |  |
|  |  | **Strategy 4** | 85,095 | 14.011 | ED:  rule out |  |  |  |  |
|  |  | **Strategy 6** | 89,690 | 14.053 | 109,252 | ED:  rule out |  |  |  |
|  |  | **Strategy 10** | 98,597 | 14.084 | 287,895 | 287,895 | 398,300 |  |  |
| **Rank** | **Optimal** | **Treatment Strategy** | **Cost, ¥** | **QALYs** | **ICERs compared to next lowest cost** | | | | |
|  |  |  |  |  | **step 1** | **step 2** | **step 3** |  |  |
| 6 | ☆ | **Strategy 3** | 56,374 | 13.978 | --- | --- | **---** |  |  |
|  |  | **Strategy 2** | 81,569 | 13.997 | ED:  rule out |  |  |  |  |
|  |  | **Strategy 4** | 85,095 | 14.011 | ED:  rule out |  |  |  |  |
|  |  | **Strategy 6** | 89,690 | 14.053 | 109,252 | ED:  rule out |  |  |  |
|  |  | **Strategy 10** | 98,597 | 14.084 | 287,895 | 287,895 | 398,300 |  |  |
| **Rank** | **Optimal** | **Treatment Strategy** | **Cost, ¥** | **QALYs** | **ICERs compared to next lowest cost** | | | | |
|  |  |  |  |  | **step 1** | **step 2** |  |  |  |
|  |  | **Strategy 2** | 81,569 | 13.997 | --- | --- |  |  |  |
|  |  | **Strategy 4** | 85,095 | 14.011 | ED:  rule out |  |  |  |  |
| 7 | ☆ | **Strategy 6** | 89,690 | 14.053 | 109,252 | **146,668** |  |  |  |
|  |  | **Strategy 10** | 98,597 | 14.084 | 287,895 | 287,895 |  |  |  |
| **Rank** | **Optimal** | **Treatment Strategy** | **Cost, ¥** | **QALYs** | **ICERs compared to next lowest cost** | | | | |
|  |  |  |  |  | **step 1** | **step 2** |  |  |  |
|  |  | **Strategy 2** | 81,569 | 13.997 | --- | --- |  |  |  |
|  |  | **Strategy 4** | 85,095 | 14.011 | ED:  rule out |  |  |  |  |
| 8 | ☆ | **Strategy 10** | 98,597 | 14.084 | 184,968 | **197,290** |  |  |  |
| **Rank** | **Optimal** | **Treatment Strategy** | **Cost, ¥** | **QALYs** | **ICERs compared to next lowest cost** | | | | |
|  |  |  |  |  | **step 1** |  |  |  |  |
| 9 | ☆ | **Strategy 2** | 81,569 | 13.997 | **---** |  |  |  |  |
| 10 | ☆ | **Strategy 4** | 85,095 | 14.011 | 264,829 |  |  |  |  |

**^a^** The treatment strategies were ranked as follows: (1) rank the strategies in order of costs and compare each strategy to its adjacent and lower-cost alternative; (2) rule out strongly dominated alternatives (with higher cost but lower QALY compared with its adjacent); (3) calculate ICERs based on the comparisons of moving to increasingly costly and increasingly effective alternatives; (4) if the ICER associated with moving to more costly alternative falls, then the lower-cost alternative used to calculate the ICER is extendedly dominated and is ruled out; (5) recalculate ICERs based on comparisons of moving to increasingly costly but increasingly effective alternatives that are neither strongly nor extendedly dominated; (6) compare the ICER with willingness-to-pay/QALY (¥212,676/QALY), if it is within willingness-to-pay/QALY, the higher-cost alternative used to calculate the ICER is the cost-effective strategy, otherwise, the lower-cost one is cost-effective

Strategy 1: metformin → metformin + sulfonylurea → metformin + insulin. Strategy 2: metformin → metformin + sulfonylurea → metformin + GLP-1 receptor agonist. Strategy 3: metformin → metformin + thiazolidinedione → metformin + insulin. Strategy 4: metformin → metformin + thiazolidinedione → metformin + GLP-1 receptor agonist. Strategy 5: metformin → metformin + α-glucosidase inhibitor → metformin + insulin. Strategy 6: metformin → metformin + α-glucosidase inhibitor → metformin + GLP-1 receptor agonist. Strategy 7: metformin → metformin + glinide → metformin + insulin. Strategy 8: metformin → metformin + glinide → metformin + GLP-1 receptor agonist. Strategy 9: metformin → metformin + DPP-4 inhibitor → metformin + insulin. Strategy 10: metformin → metformin + DPP-4 inhibitor → metformin + GLP-1 receptor agonist.

ICER, incremental cost-effectiveness ratio. QALY, quality-adjusted life-year. SD, strongly dominated. ED, extendedly dominated. DPP-4, dipeptidyl peptidase 4. GLP-1, glucagon-like peptide 1.

**Table S8. Detailed results for the ten pharmacologic combination strategies: base-case analysis**

| **Pharmacologic Combination Strategy** | **Strategy 1: metformin → metformin + sulfonylurea → metformin + insulin** | | **Strategy 2: metformin → metformin + sulfonylurea → metformin + GLP-1 receptor agonist** | |
| --- | --- | --- | --- | --- |
| **Total Events (per cohort)** | **Strategy 1** | | **Strategy 2** | |
|  | Non-Fatal | Fatal | Non-Fatal | Fatal |
| Ischemic Heart Disease | 1171.78 | 0.00 | 1168.86 | 0.00 |
| Myocardial Infarction | 1355.97 | 1838.13 | 1352.26 | 1824.96 |
| Congestive Heart Failure | 767.20 | 89.85 | 757.63 | 88.26 |
| Stroke | 823.74 | 252.66 | 807.20 | 246.26 |
| Blindness | 789.20 | 0.00 | 784.55 | 0.00 |
| End-stage Renal Disease | 225.25 | 27.78 | 216.86 | 26.72 |
| Amputation | 300.85 | 37.17 | 292.66 | 36.07 |
| Fatal: Macrovascular |  | 2180.64 |  | 2159.47 |
| Fatal: Microvascular |  | 64.95 |  | 62.80 |
| Fatal: Other |  | 7539.43 |  | 7559.95 |
| Symptomatic Hypoglycemia | 7548.47 |  | 7557.51 |  |
| Severe Hypoglycemia | 154.67 |  | 154.85 |  |
| **Total Costs, ¥ (per cohort)** | **Strategy 1** | | **Strategy 2** | |
| Ischemic Heart Disease | 43,117,256 | | 43,105,192 | |
| Myocardial Infarction | 144,367,105 | | 143,937,394 | |
| Congestive Heart Failure | 27,269,133 | | 27,021,880 | |
| Stroke | 42,193,430 | | 41,548,650 | |
| Blindness | 35,194,220 | | 35,149,870 | |
| End-stage Renal Disease | 5,994,755 | | 5,825,926 | |
| Amputation | 8,333,503 | | 8,165,224 | |
| Hypoglycemia | 433,962 | | 434,282 | |
| Glucose-lowering Treatments | 222,328,065 | | 510,496,851 | |
| BMI-related | 0 | | 0 | |
| Total | 529,231,429 | | 815,685,268 | |
| **Cost-Effectiveness (per patient)** | **Strategy 1** | | **Strategy 2** | |
| Discounted Cost | 52,923 | | 81,569 | |
| Discounted QALYs | 13.965 | | 13.997 | |
| Discounted Life Years | 15.718 | | 15.729 | |
| **Pharmacologic Combination Strategy** | **Strategy 3: metformin → metformin + thiazolidinedione → metformin + insulin** | | **Strategy 4: metformin → metformin + thiazolidinedione → metformin + GLP-1 receptor agonist** | |
| **Total Events (per cohort)** | **Strategy 3** | | **Strategy 4** | |
|  | **Non-Fatal** | **Fatal** | **Non-Fatal** | **Fatal** |
| Ischemic Heart Disease | 1164.15 | 0.00 | 1160.63 | 0.00 |
| Myocardial Infarction | 1345.10 | 1836.88 | 1340.93 | 1822.61 |
| Congestive Heart Failure | 770.89 | 90.44 | 761.40 | 88.86 |
| Stroke | 822.79 | 253.53 | 805.96 | 247.07 |
| Blindness | 794.67 | 0.00 | 790.09 | 0.00 |
| End-stage Renal Disease | 225.07 | 27.88 | 216.68 | 26.80 |
| Amputation | 303.58 | 37.52 | 295.37 | 36.40 |
| Fatal: Macrovascular |  | 2180.84 |  | 2158.54 |
| Fatal: Microvascular |  | 65.40 |  | 63.21 |
| Fatal: Other |  | 7538.79 |  | 7560.44 |
| Symptomatic Hypoglycemia | 4570.34 |  | 4579.87 |  |
| Severe Hypoglycemia | 93.89 |  | 94.08 |  |
| **Total Costs, ¥ (per cohort)** | **Strategy 3** | | **Strategy 4** | |
| Ischemic Heart Disease | 42,669,923 | | 42,646,296 | |
| Myocardial Infarction | 143,153,981 | | 142,679,780 | |
| Congestive Heart Failure | 27,427,367 | | 27,183,686 | |
| Stroke | 42,132,104 | | 41,481,982 | |
| Blindness | 35,471,816 | | 35,431,618 | |
| End-stage Renal Disease | 5,980,428 | | 5,812,663 | |
| Amputation | 8,428,245 | | 8,259,348 | |
| Hypoglycemia | 260,218 | | 260,557 | |
| Glucose-lowering Treatments | 258,217,906 | | 547,196,810 | |
| BMI-related | 0 | | 0 | |
| Total | 563,741,987 | | 850,952,739 | |
| **Cost-Effectiveness (per patient)** | **Strategy 3** | | **Strategy 4** | |
| Discounted Cost | 56,374 | | 85,095 | |
| Discounted QALYs | 13.978 | | 14.011 | |
| Discounted Life Years | 15.727 | | 15.739 | |

| **Pharmacologic Combination Strategy** | **Strategy 5: metformin → metformin + α-glucosidase inhibitor → metformin + insulin** | | **Strategy 6: metformin → metformin + α-glucosidase inhibitor → metformin + GLP-1 receptor agonist** | |
| --- | --- | --- | --- | --- |
| **Total Events (per cohort)** | **Strategy 5** | | **Strategy 6** | |
|  | Non-Fatal | Fatal | Non-Fatal | Fatal |
| Ischemic Heart Disease | 1150.96 | 0.00 | 1146.95 | 0.00 |
| Myocardial Infarction | 1329.56 | 1826.89 | 1324.96 | 1811.95 |
| Congestive Heart Failure | 774.15 | 90.80 | 764.73 | 89.20 |
| Stroke | 823.74 | 254.33 | 806.72 | 247.73 |
| Blindness | 796.13 | 0.00 | 791.61 | 0.00 |
| End-stage Renal Disease | 226.48 | 28.02 | 218.08 | 26.94 |
| Amputation | 304.63 | 37.62 | 296.42 | 36.51 |
| Fatal: Macrovascular |  | 2172.02 |  | 2148.88 |
| Fatal: Microvascular |  | 65.65 |  | 63.45 |
| Fatal: Other |  | 7547.00 |  | 7569.43 |
| Symptomatic Hypoglycemia | 3031.74 |  | 3041.64 |  |
| Severe Hypoglycemia | 63.52 |  | 63.72 |  |
| **Total Costs, ¥ (per cohort)** | **Strategy 5** | | **Strategy 6** | |
| Ischemic Heart Disease | 41,983,786 | | 41,949,684 | |
| Myocardial Infarction | 141,244,771 | | 140,743,950 | |
| Congestive Heart Failure | 27,556,090 | | 27,312,806 | |
| Stroke | 42,152,850 | | 41,490,956 | |
| Blindness | 35,535,818 | | 35,502,358 | |
| End-stage Renal Disease | 6,035,386 | | 5,867,772 | |
| Amputation | 8,463,950 | | 8,296,414 | |
| Hypoglycemia | 173,351 | | 173,703 | |
| Glucose-lowering Treatments | 304,263,261 | | 595,562,653 | |
| BMI-related | 0 | | 0 | |
| Total | 607,409,263 | | 896,900,296 | |
| **Cost-Effectiveness (per patient)** | **Strategy 5** | | **Strategy 6** | |
| Discounted Cost | 60,741 | | 89,690 | |
| Discounted QALYs | 14.019 | | 14.053 | |
| Discounted Life Years | 15.747 | | 15.759 | |

| **Pharmacologic Combination Strategy** | **Strategy 7: metformin → metformin + glinide → metformin + insulin** | | **Strategy 8: metformin → metformin + glinide → metformin + GLP-1 receptor agonist** | |
| --- | --- | --- | --- | --- |
| **Total Events (per cohort)** | **Strategy 7** | | **Strategy 8** | |
|  | Non-Fatal | Fatal | Non-Fatal | Fatal |
| Ischemic Heart Disease | 1182.62 | 0.00 | 1180.03 | 0.00 |
| Myocardial Infarction | 1371.32 | 1847.22 | 1367.96 | 1834.82 |
| Congestive Heart Failure | 767.34 | 89.56 | 757.78 | 87.96 |
| Stroke | 832.86 | 253.94 | 816.50 | 247.56 |
| Blindness | 786.79 | 0.00 | 782.16 | 0.00 |
| End-stage Renal Disease | 227.93 | 27.96 | 219.58 | 26.91 |
| Amputation | 301.42 | 37.12 | 293.21 | 36.02 |
| Fatal: Macrovascular |  | 2190.72 |  | 2170.34 |
| Fatal: Microvascular |  | 65.09 |  | 62.93 |
| Fatal: Other |  | 7529.95 |  | 7549.78 |
| Symptomatic Hypoglycemia | 3020.48 |  | 3029.22 |  |
| Severe Hypoglycemia | 63.30 |  | 63.47 |  |
| **Total Costs, ¥ (per cohort)** | **Strategy 7** | | **Strategy 8** | |
| Ischemic Heart Disease | 43,667,994 | | 43,665,169 | |
| Myocardial Infarction | 146,168,569 | | 145,772,522 | |
| Congestive Heart Failure | 27,296,790 | | 27,049,063 | |
| Stroke | 42,792,033 | | 42,150,560 | |
| Blindness | 35,058,929 | | 35,014,139 | |
| End-stage Renal Disease | 6,114,503 | | 5,946,223 | |
| Amputation | 8,362,242 | | 8,192,651 | |
| Hypoglycemia | 172,904 | | 173,213 | |
| Glucose-lowering Treatments | 247,659,870 | | 543,456,898 | |
| BMI-related | 0 | | 0 | |
| Total | 557,293,835 | | 851,420,437 | |
| **Cost-Effectiveness (per patient)** | **Strategy 7** | | **Strategy 8** | |
| Discounted Cost | 55,729 | | 85,142 | |
| Discounted QALYs | 14.085 | | 14.117 | |
| Discounted Life Years | 15.694 | | 15.705 | |

| **Pharmacologic Combination Strategy** | **Strategy 9: metformin → metformin + DPP-4 inhibitor → metformin + insulin** | | **Strategy 10: metformin → metformin + DPP-4 inhibitor → metformin + GLP-1 receptor agonist** | |
| --- | --- | --- | --- | --- |
| **Total Events (per cohort)** | **Strategy 9** | | **Strategy 10** | |
|  | Non-Fatal | Fatal | Non-Fatal | Fatal |
| Ischemic Heart Disease | 1158.67 | 0.00 | 1155.45 | 0.00 |
| Myocardial Infarction | 1338.26 | 1828.74 | 1334.23 | 1814.87 |
| Congestive Heart Failure | 769.51 | 90.30 | 759.92 | 88.71 |
| Stroke | 817.95 | 252.52 | 801.21 | 246.04 |
| Blindness | 793.12 | 0.00 | 788.52 | 0.00 |
| End-stage Renal Disease | 223.99 | 27.79 | 215.59 | 26.72 |
| Amputation | 301.62 | 37.36 | 293.38 | 36.26 |
| Fatal: Macrovascular |  | 2171.57 |  | 2149.62 |
| Fatal: Microvascular |  | 65.15 |  | 62.98 |
| Fatal: Other |  | 7547.70 |  | 7568.94 |
| Symptomatic Hypoglycemia | 3581.02 |  | 3590.44 |  |
| Severe Hypoglycemia | 73.70 |  | 73.89 |  |
| **Total Costs, ¥ (per cohort)** | **Strategy 9** | | **Strategy 10** | |
| Ischemic Heart Disease | 42,446,485 | | 42,429,451 | |
| Myocardial Infarction | 142,326,573 | | 141,870,084 | |
| Congestive Heart Failure | 27,344,549 | | 27,098,462 | |
| Stroke | 41,818,178 | | 41,167,445 | |
| Blindness | 35,396,563 | | 35,354,779 | |
| End-stage Renal Disease | 5,934,075 | | 5,765,686 | |
| Amputation | 8,355,829 | | 8,186,330 | |
| Hypoglycemia | 202,411 | | 202,745 | |
| Glucose-lowering Treatments | 390,845,750 | | 683,893,490 | |
| BMI-related | 0 | | 0 | |
| Total | 694,670,412 | | 985,968,473 | |
| **Cost-Effectiveness (per patient)** | **Strategy 9** | | **Strategy 10** | |
| Discounted Cost | 69,467 | | 98,597 | |
| Discounted QALYs | 14.051 | | 14.084 | |
| Discounted Life Years | 15.741 | | 15.752 | |

Per cohort: 10,000 Chinese patients with newly diagnosed type 2 diabetes.

BMI, body mass index. DPP-4, dipeptidyl peptidase 4. GLP-1, glucagon-like peptide 1. QALY, quality-adjusted life-year.


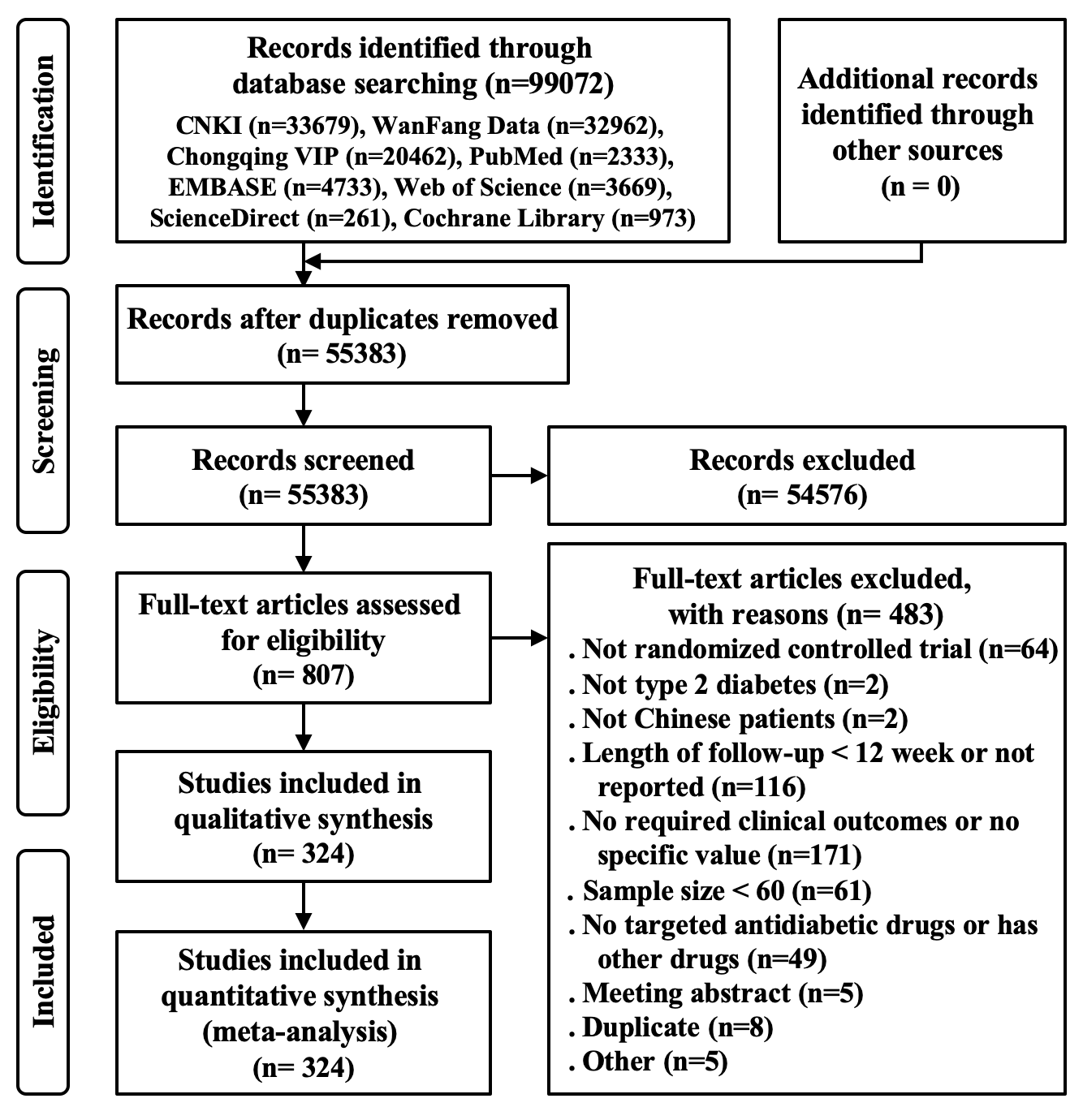


**Fig. S1. PRISMA flow diagram of study selection.** PRISMA, Preferred Reporting Items for Systematic Reviews and Meta-Analysis. CNKI, China National Knowledge Infrastructure.


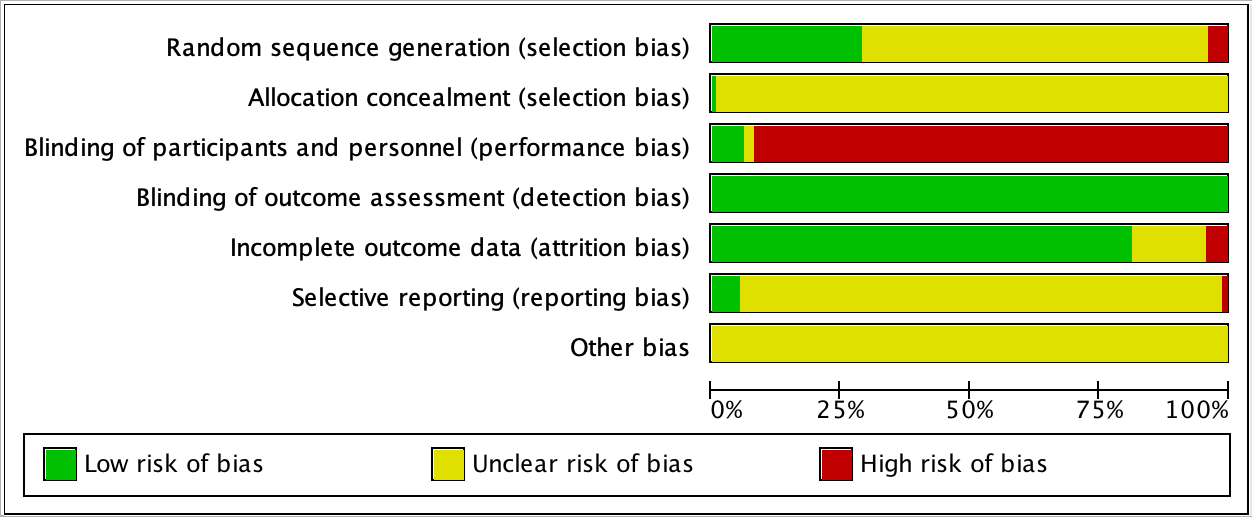


**Fig. S2. Risk of bias graph**

Note: The study quality and the risk of bias within individual studies were assessed by using the Cochrane Collaboration’s risk of bias assessment tool.

Reference: Higgins, J. P. & Green, S. Cochrane Handbook for Systematic Reviews of Interventions Version 5.1.0 [updated March 2011], http://handbook-5-1.cochrane.org/.


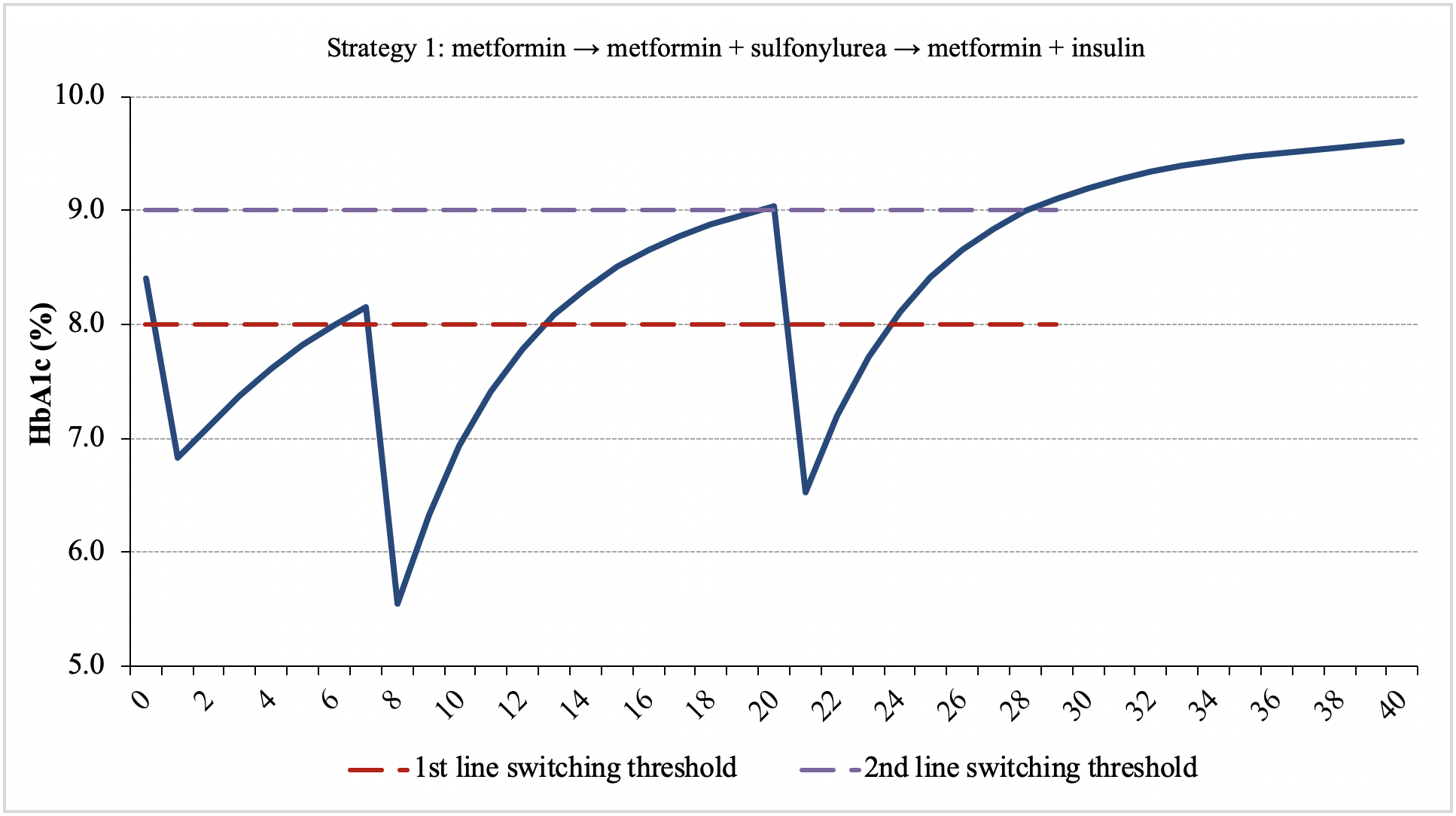

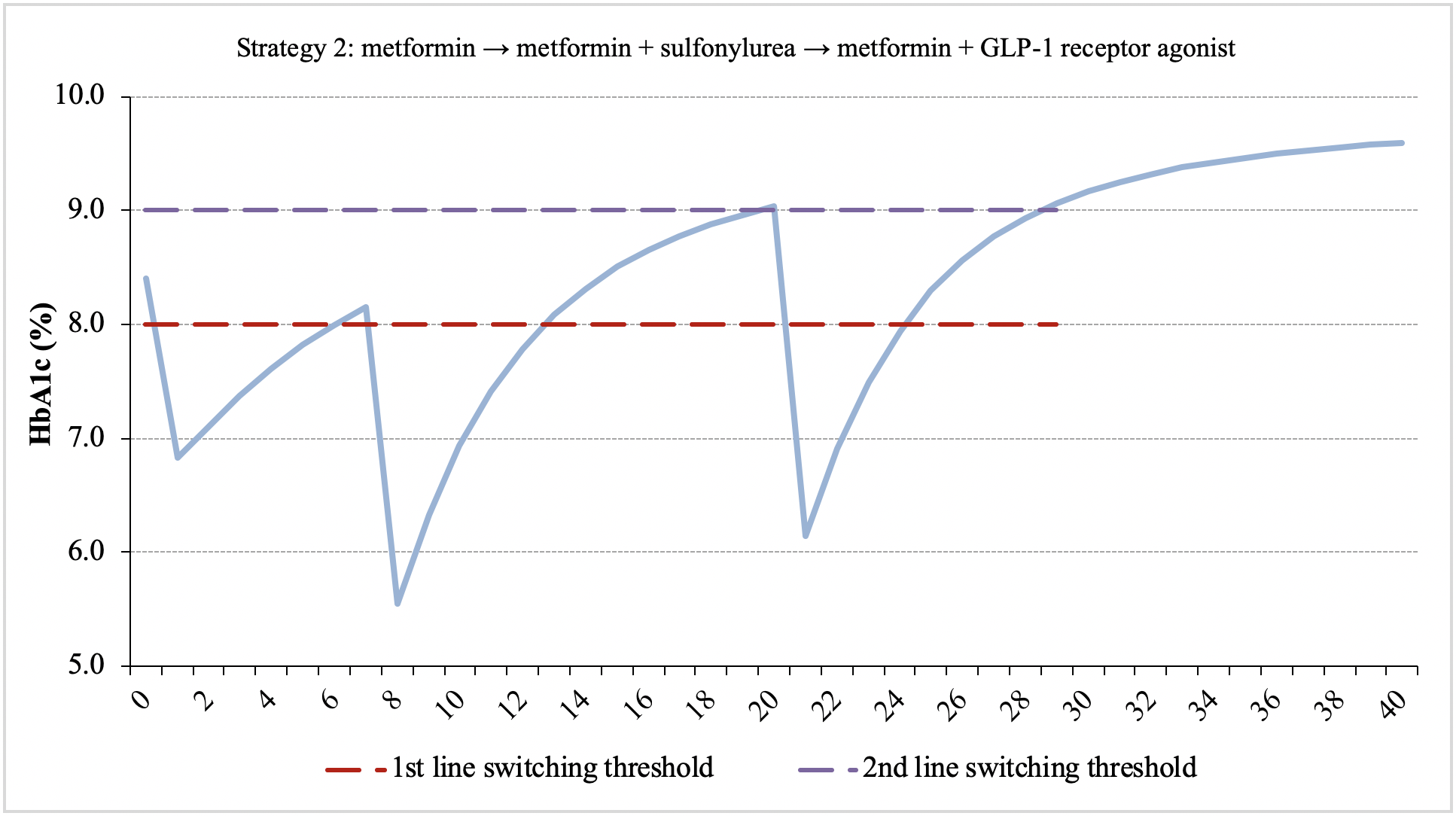


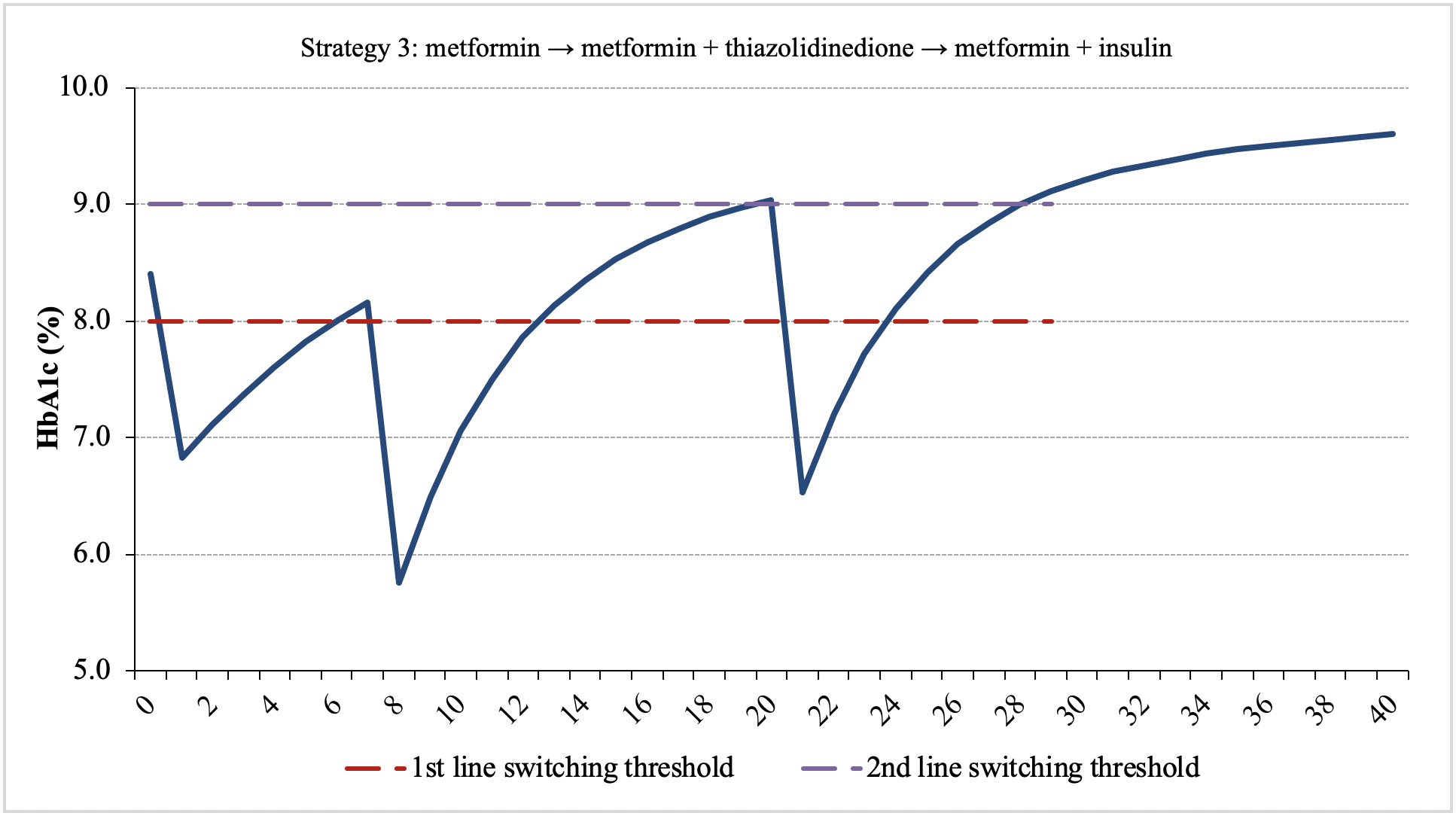

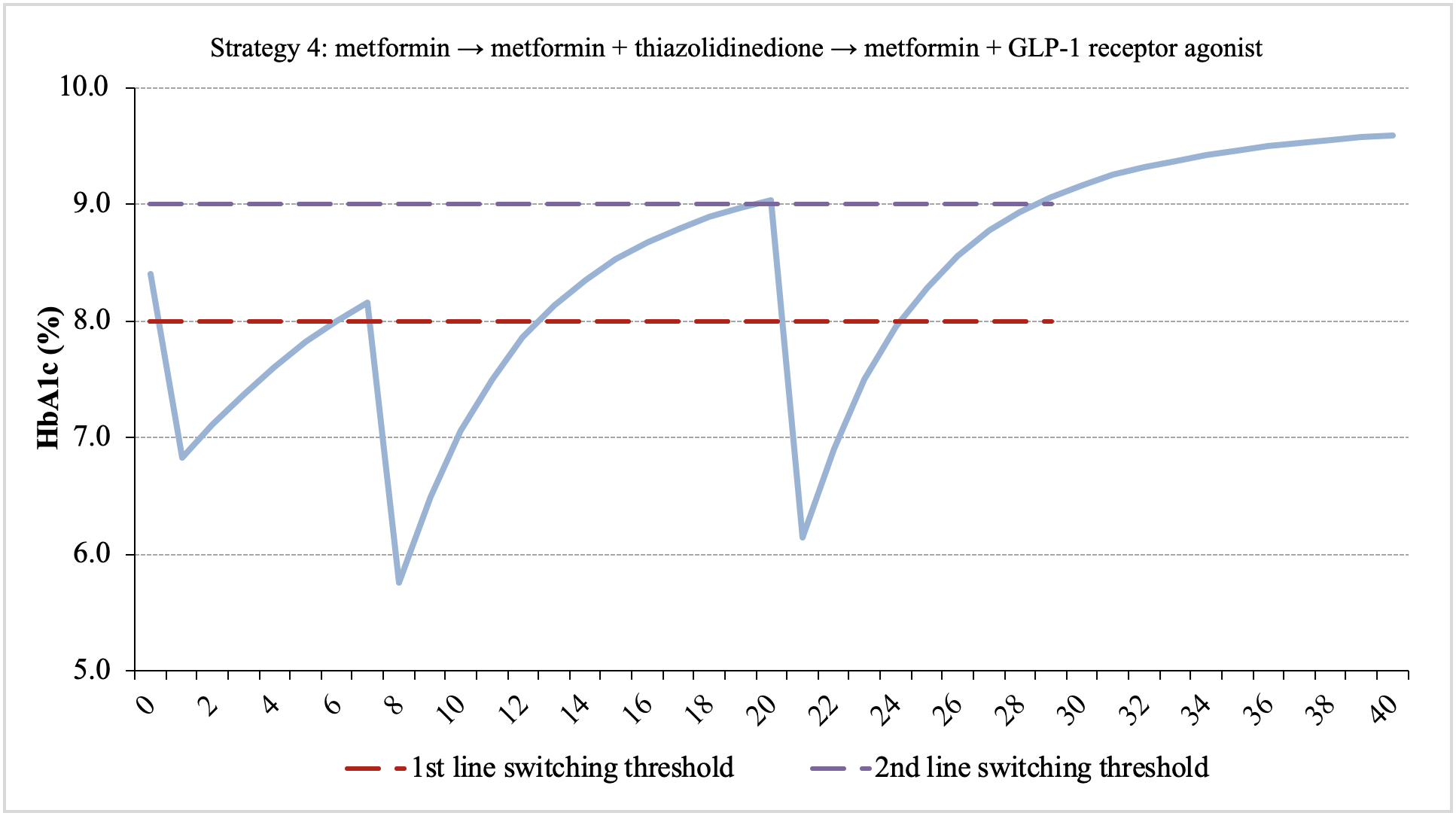


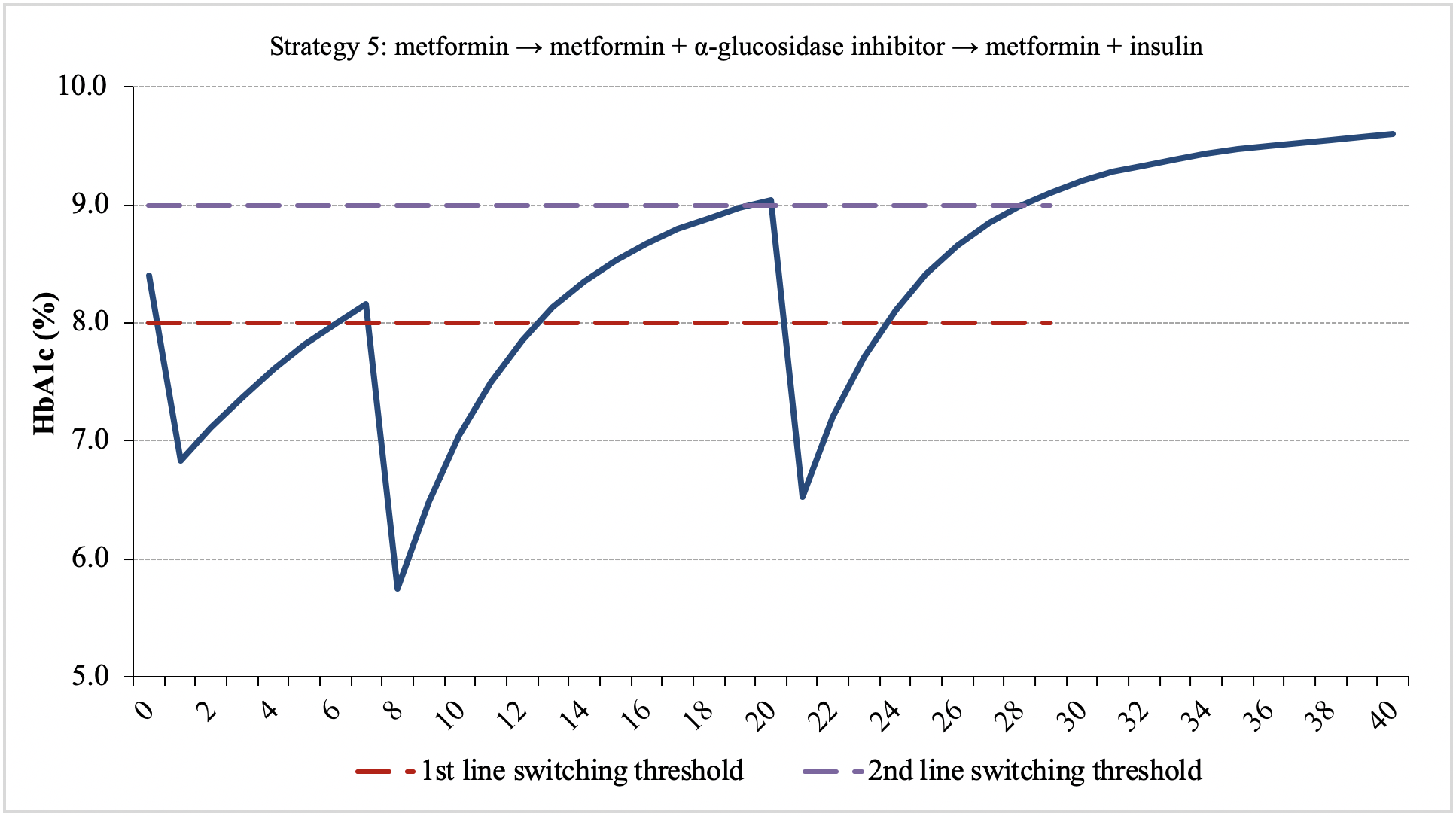

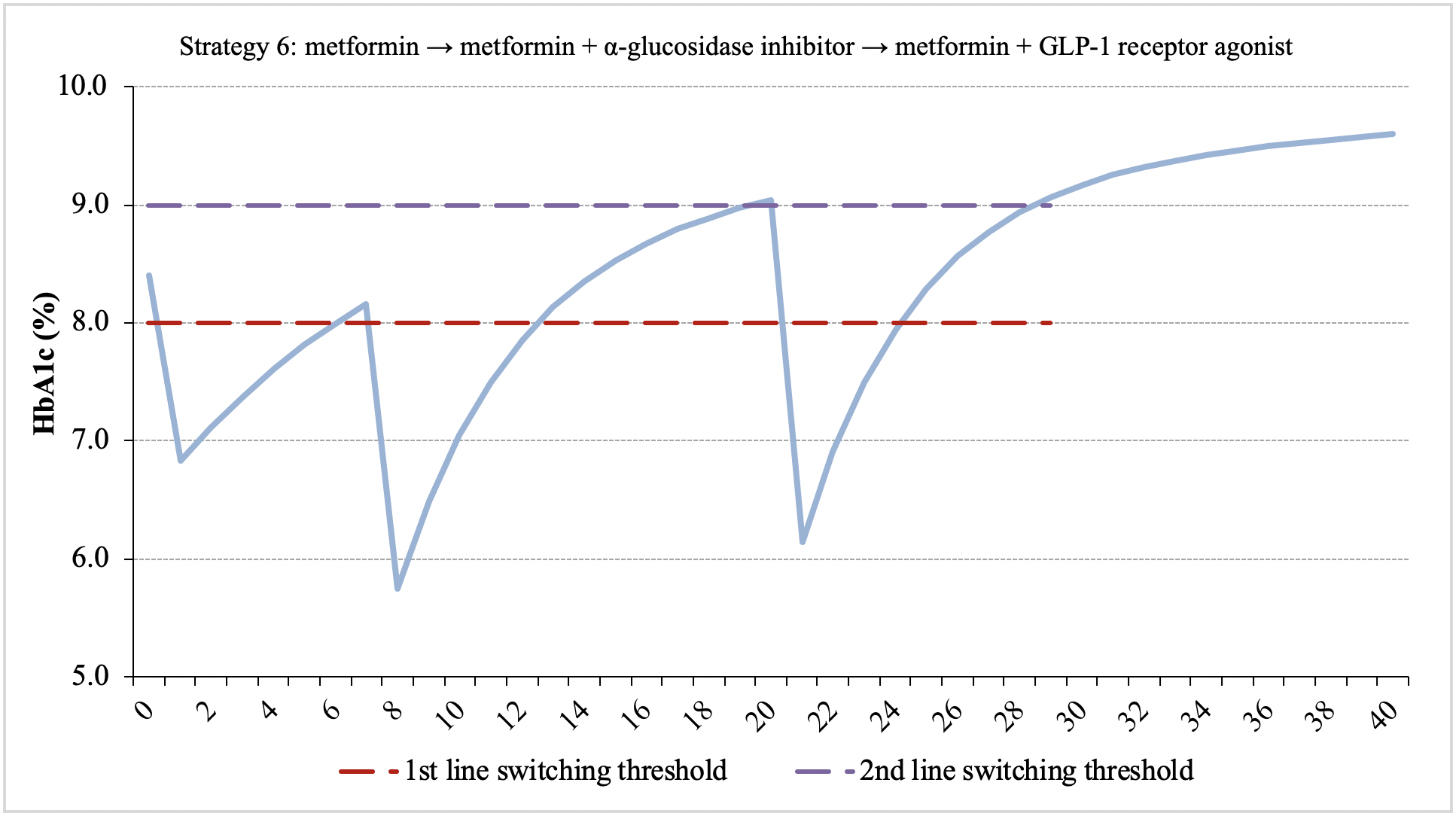


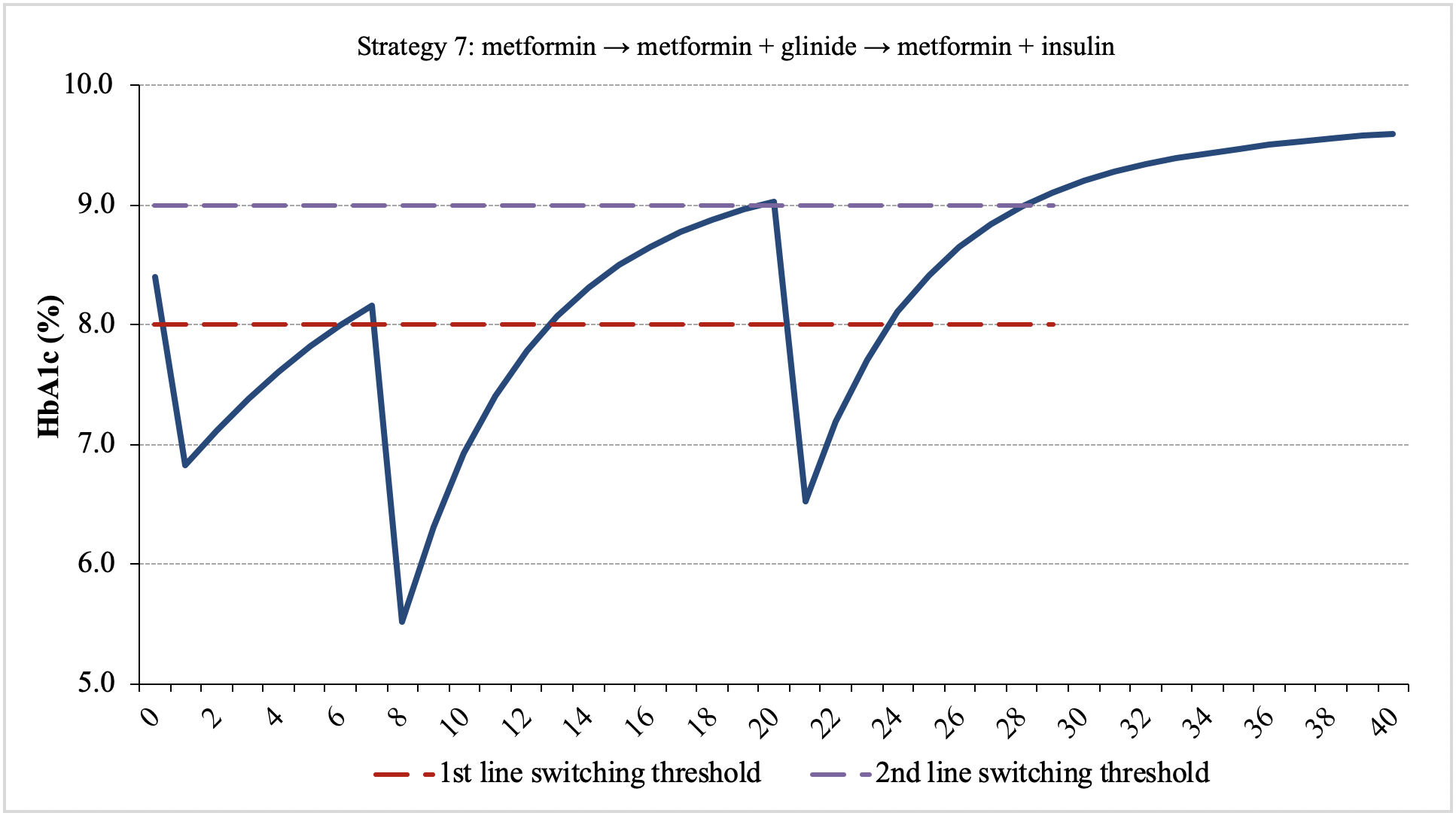

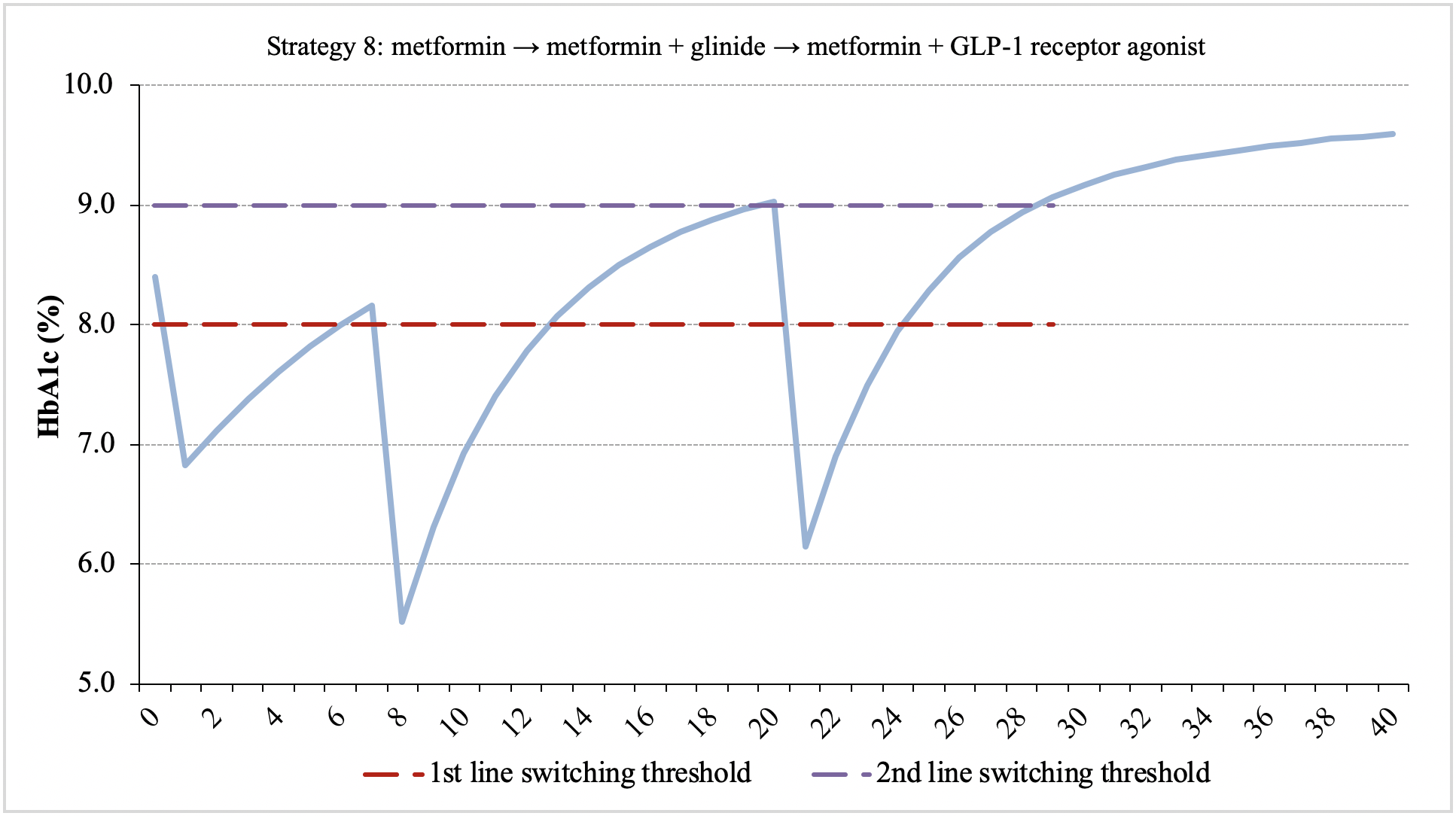


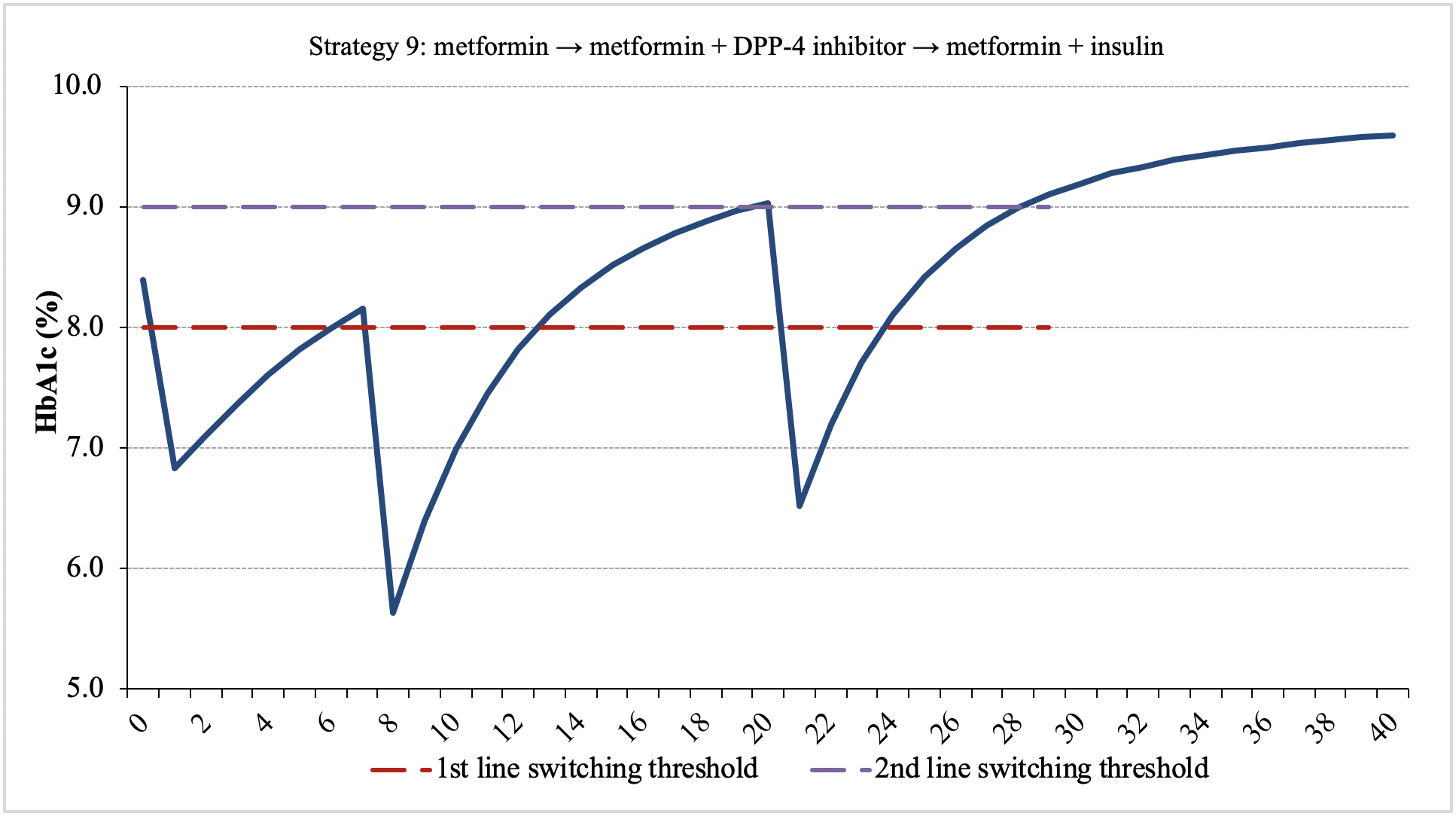

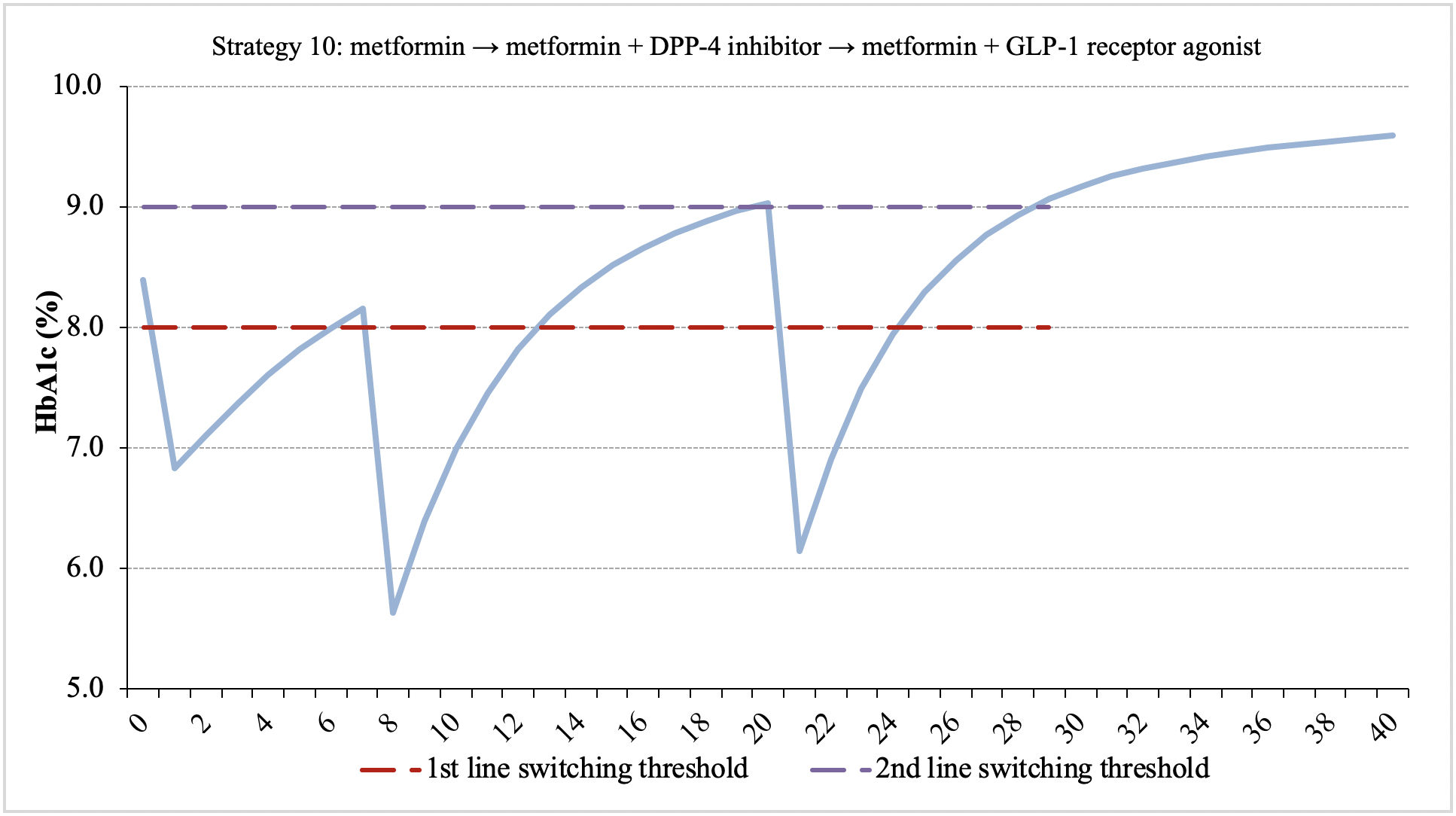


**Fig. S3. The trajectories of HbA1c in ten pharmacologic combination strategies over time: base-case analysis**. DPP-4, dipeptidyl peptidase 4. GLP-1, glucagon-like peptide 1. HbA1c, glycosylated hemoglobin Alc.


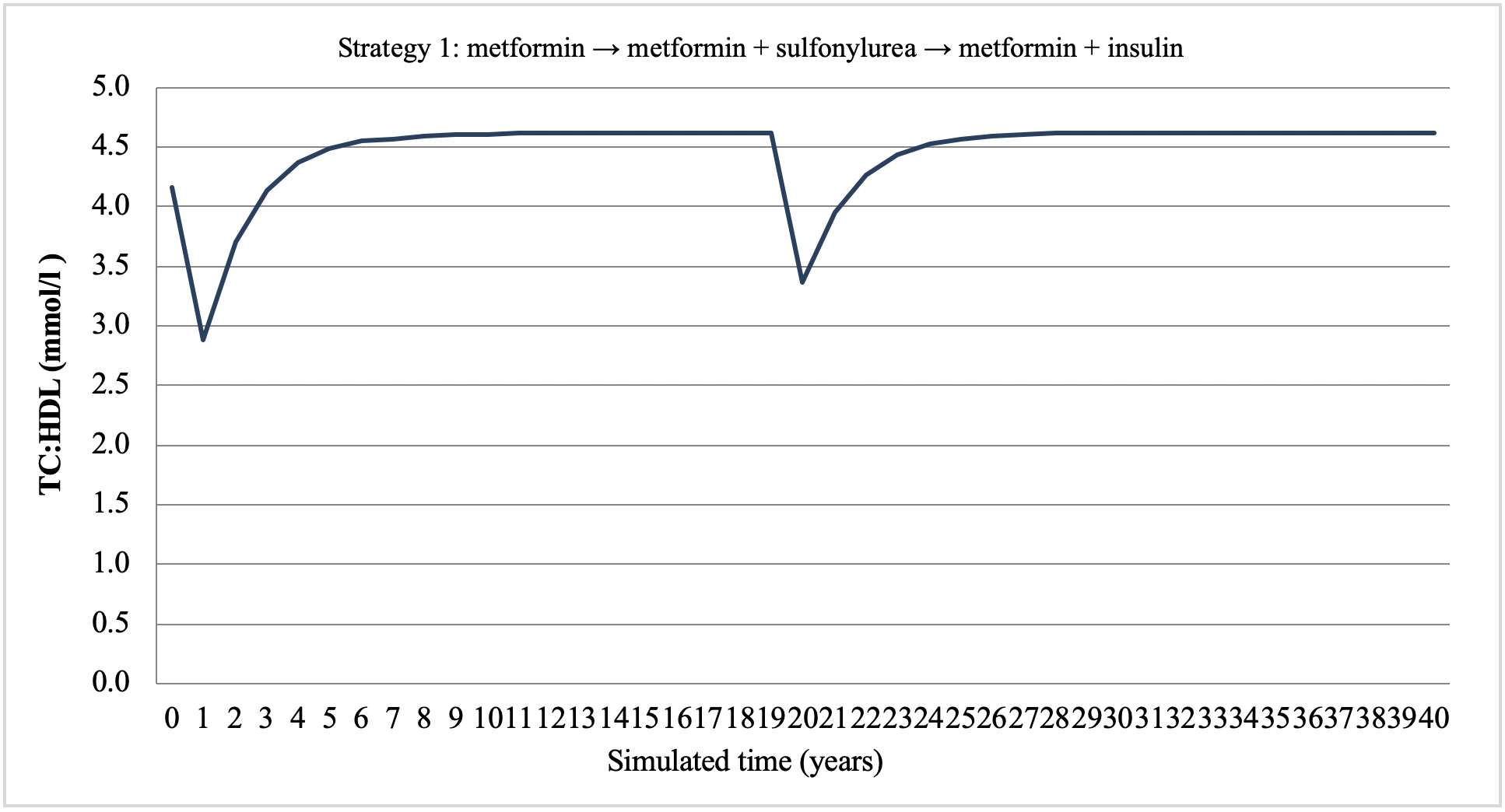

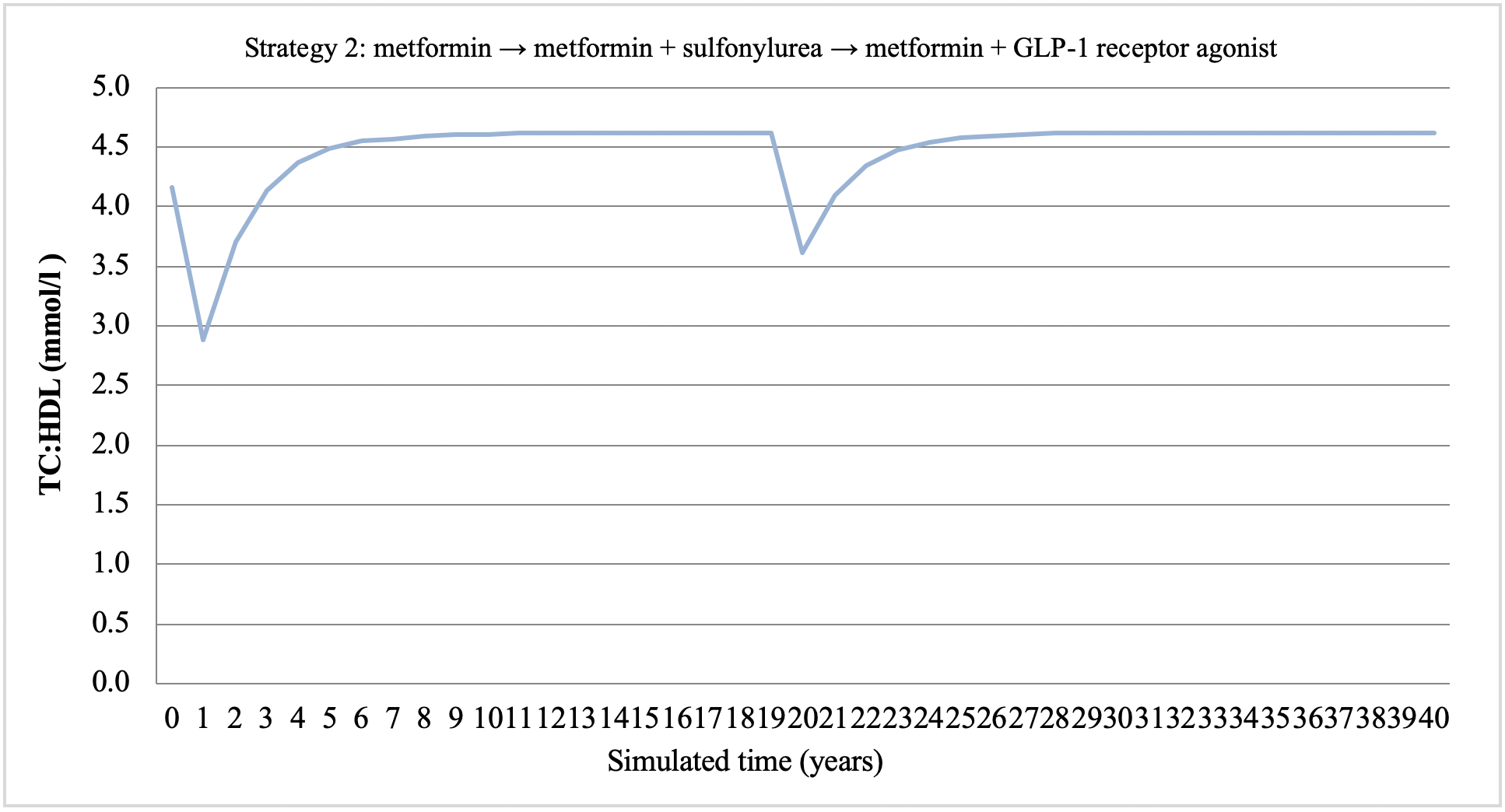

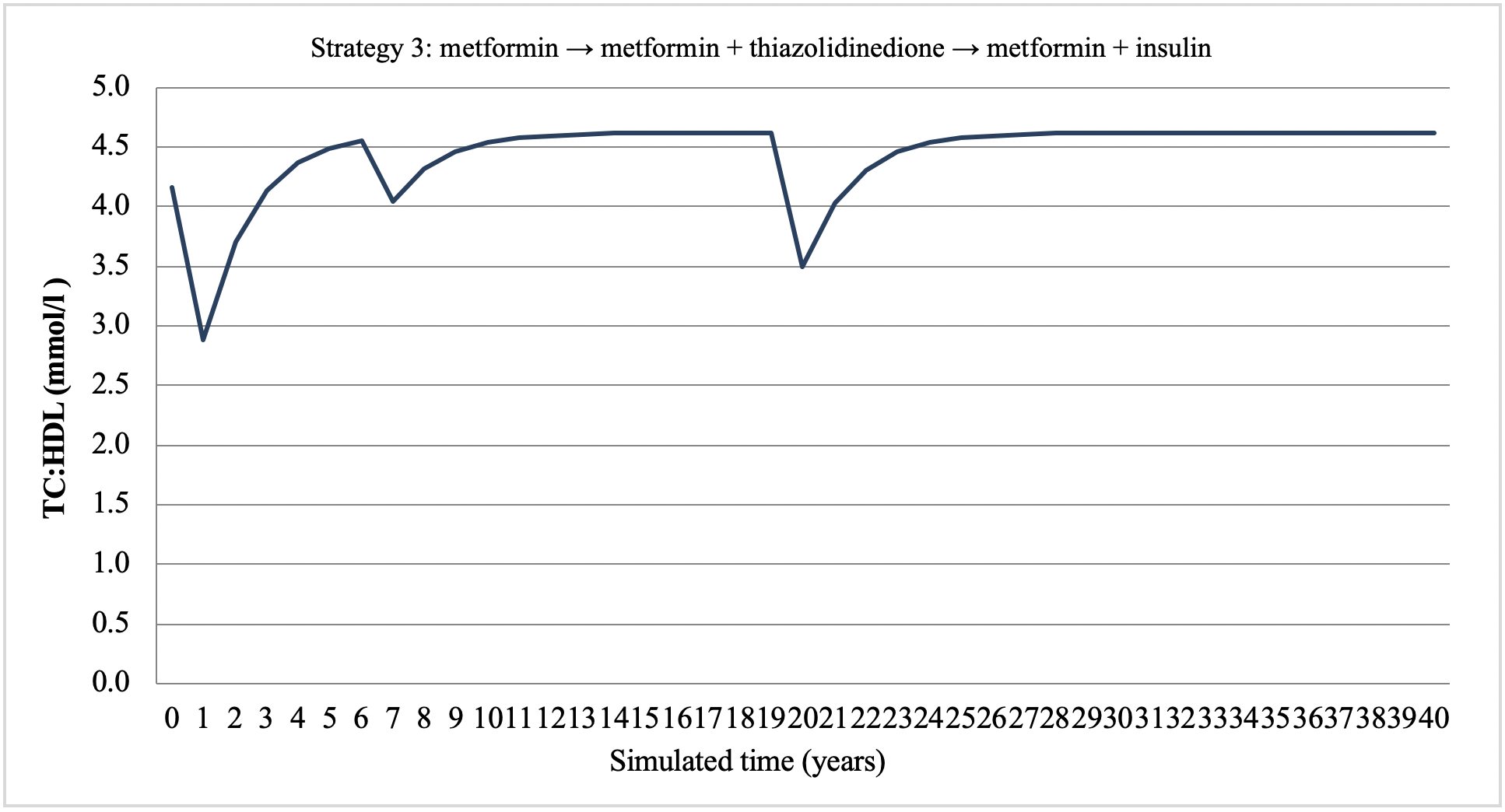

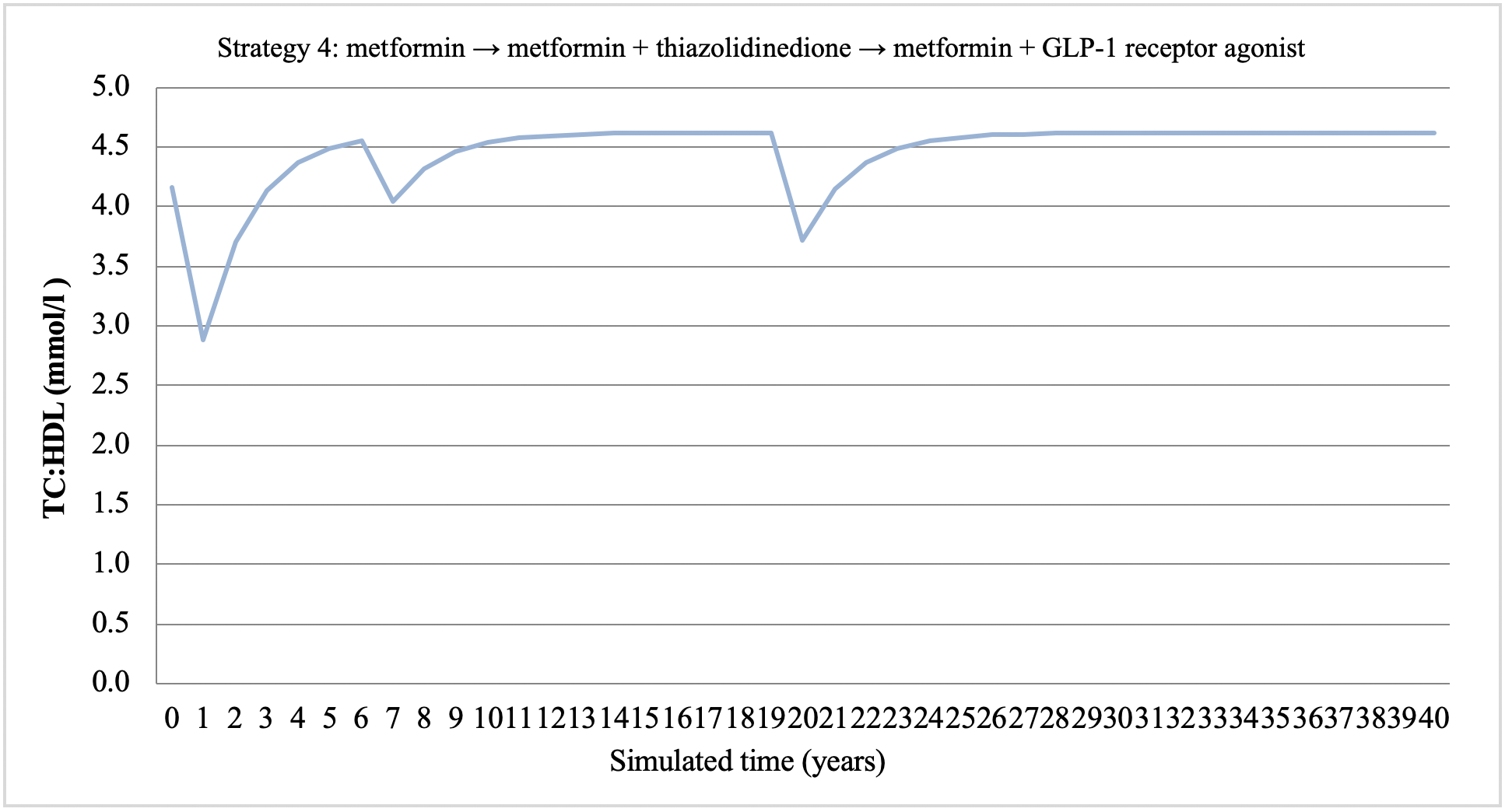


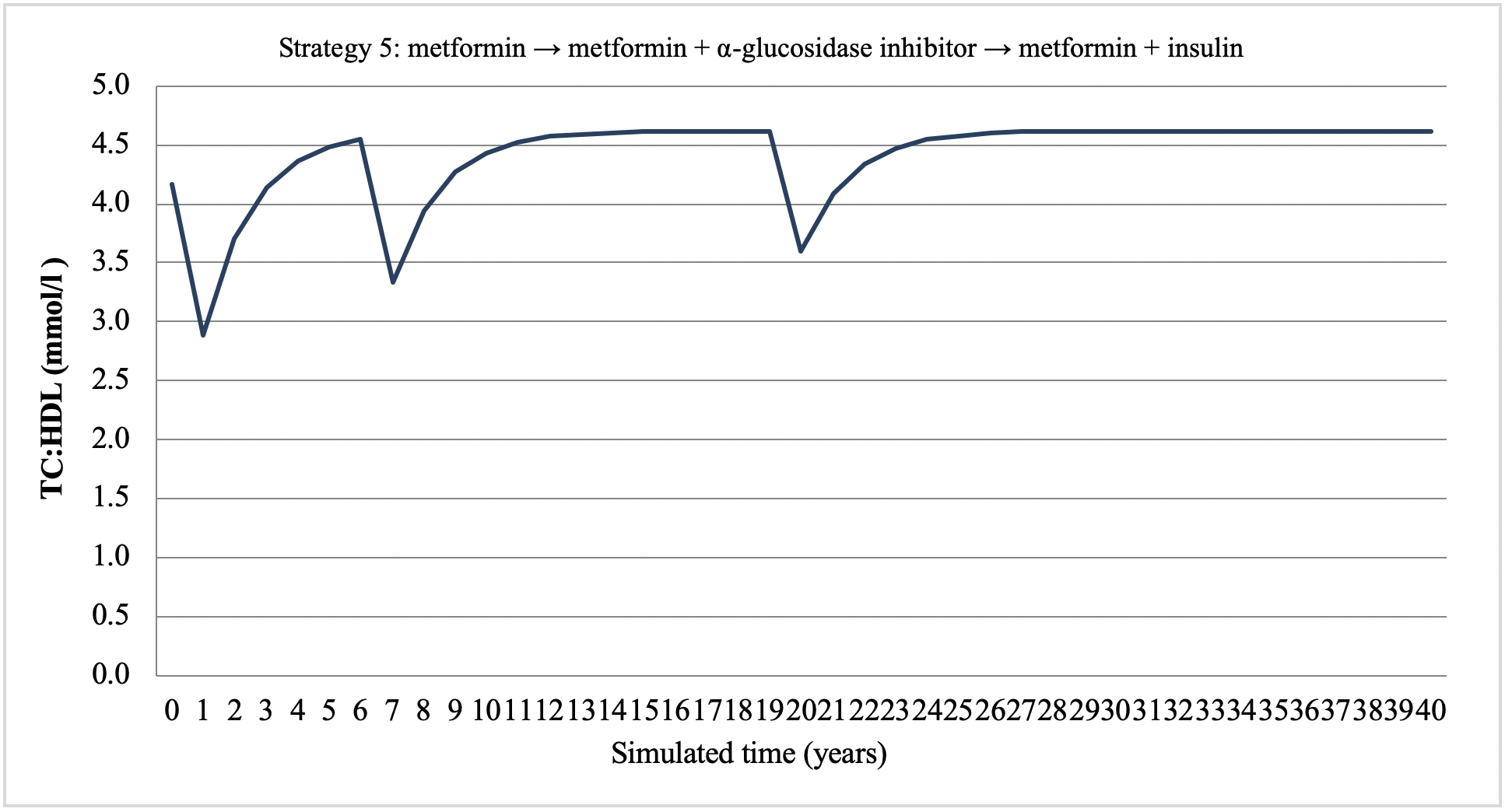

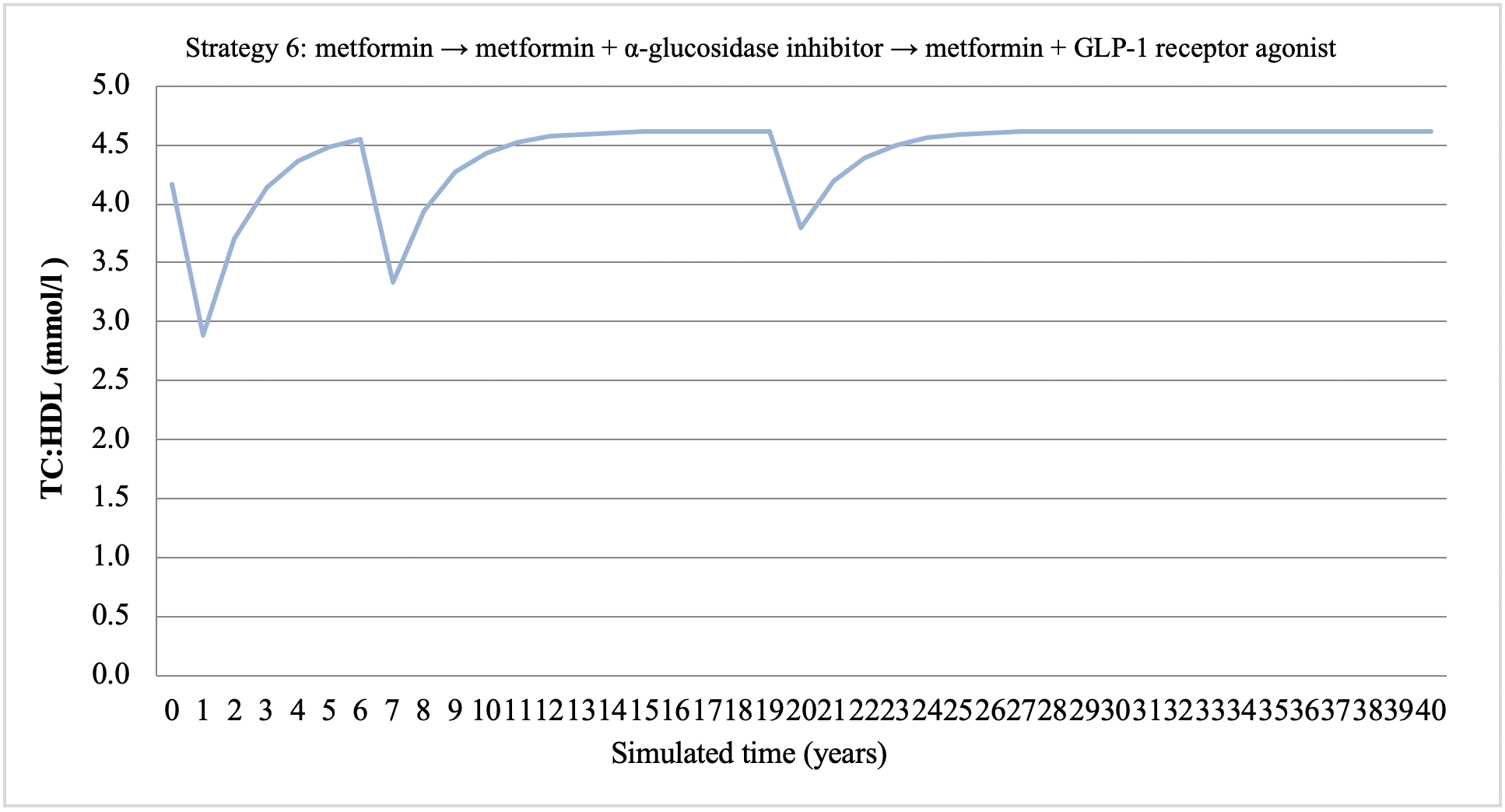


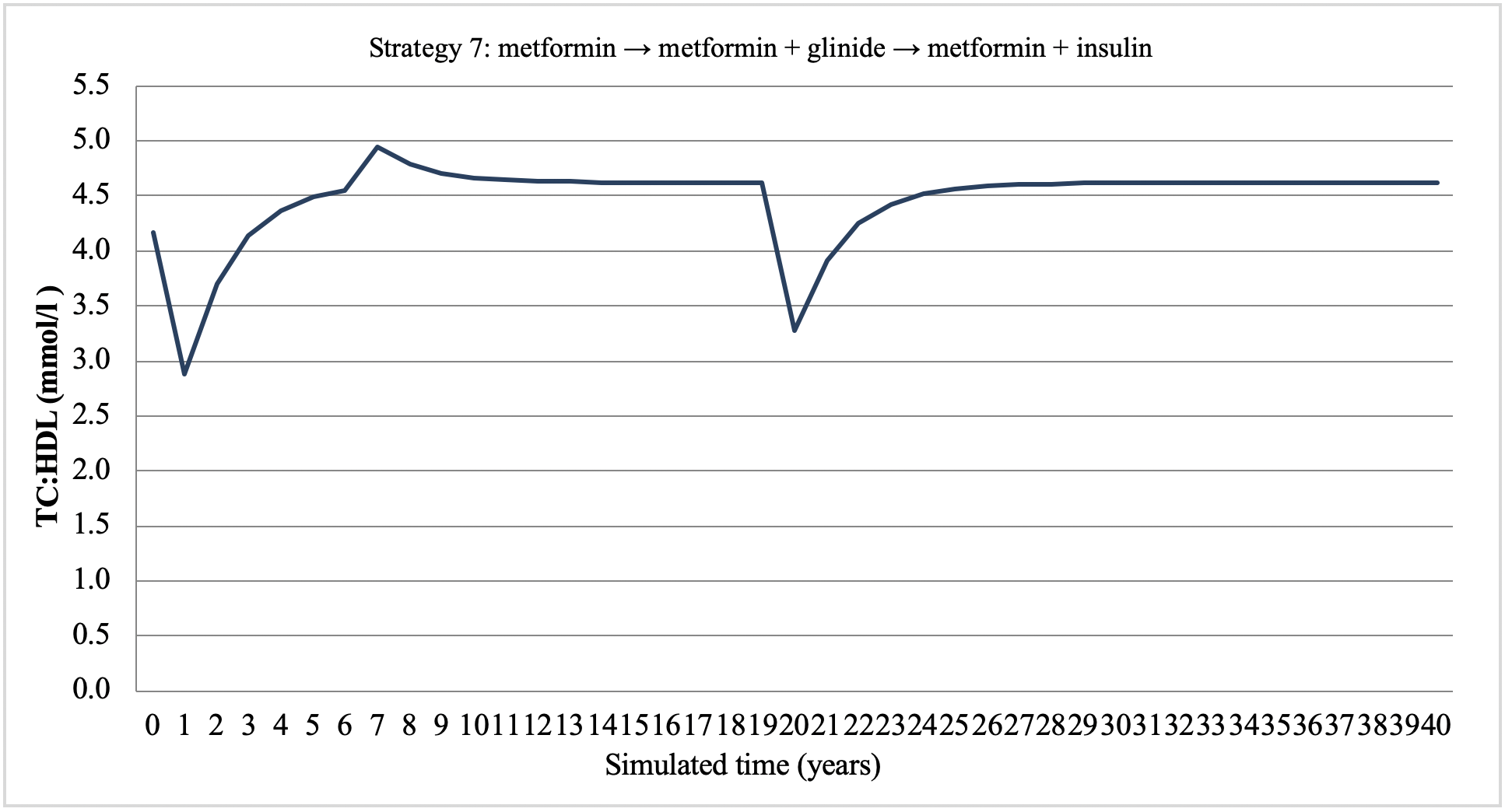

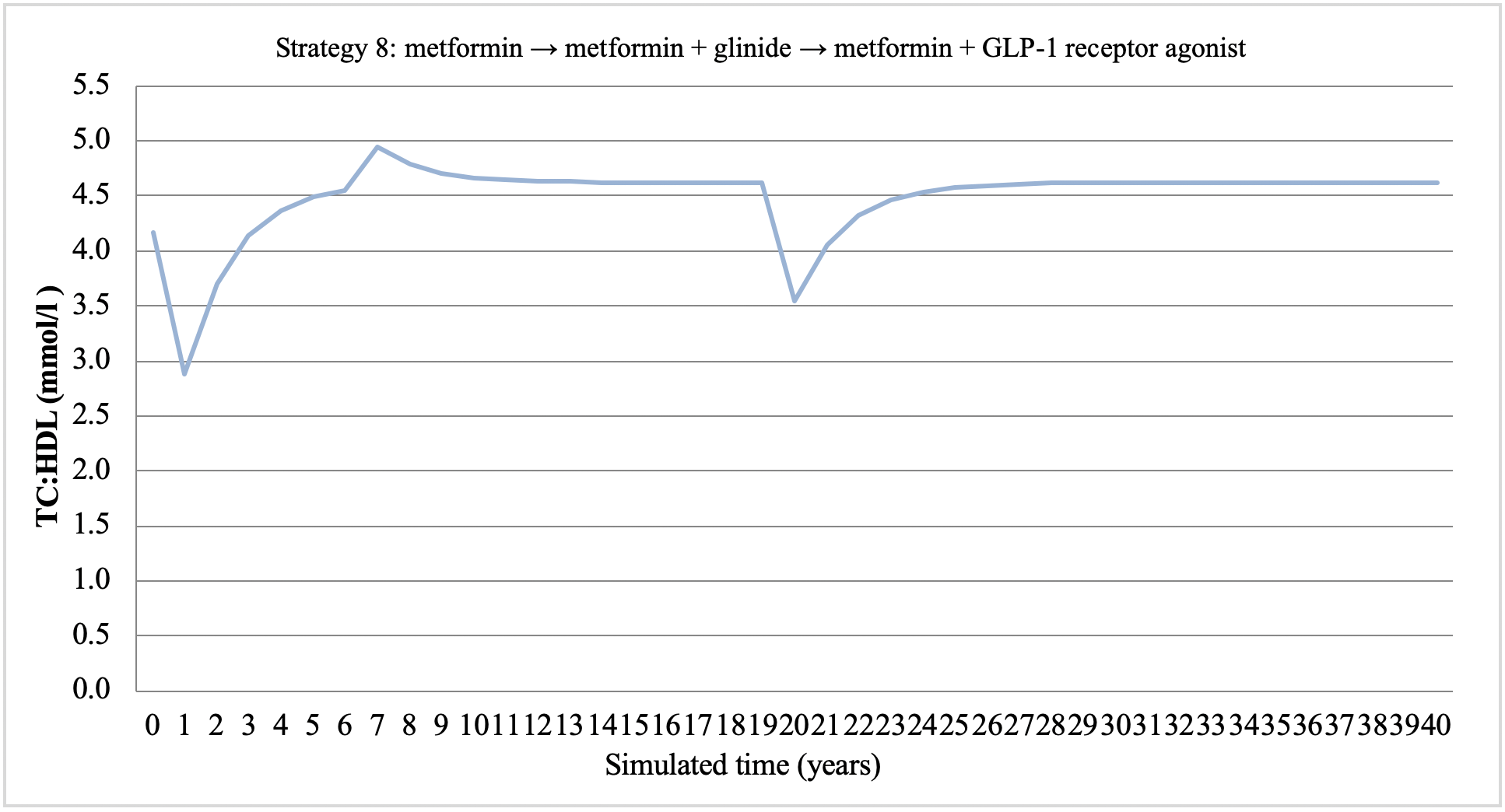


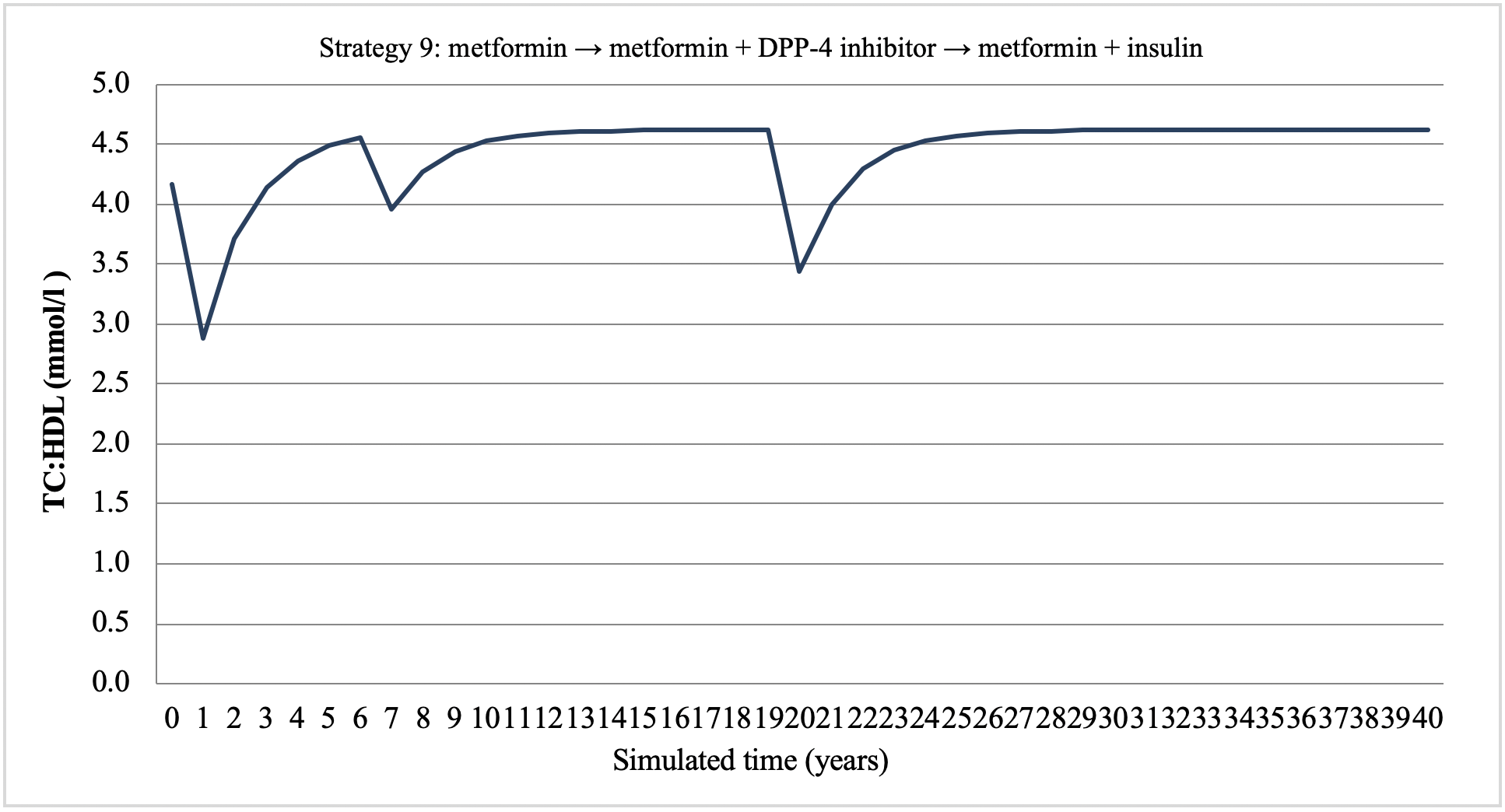

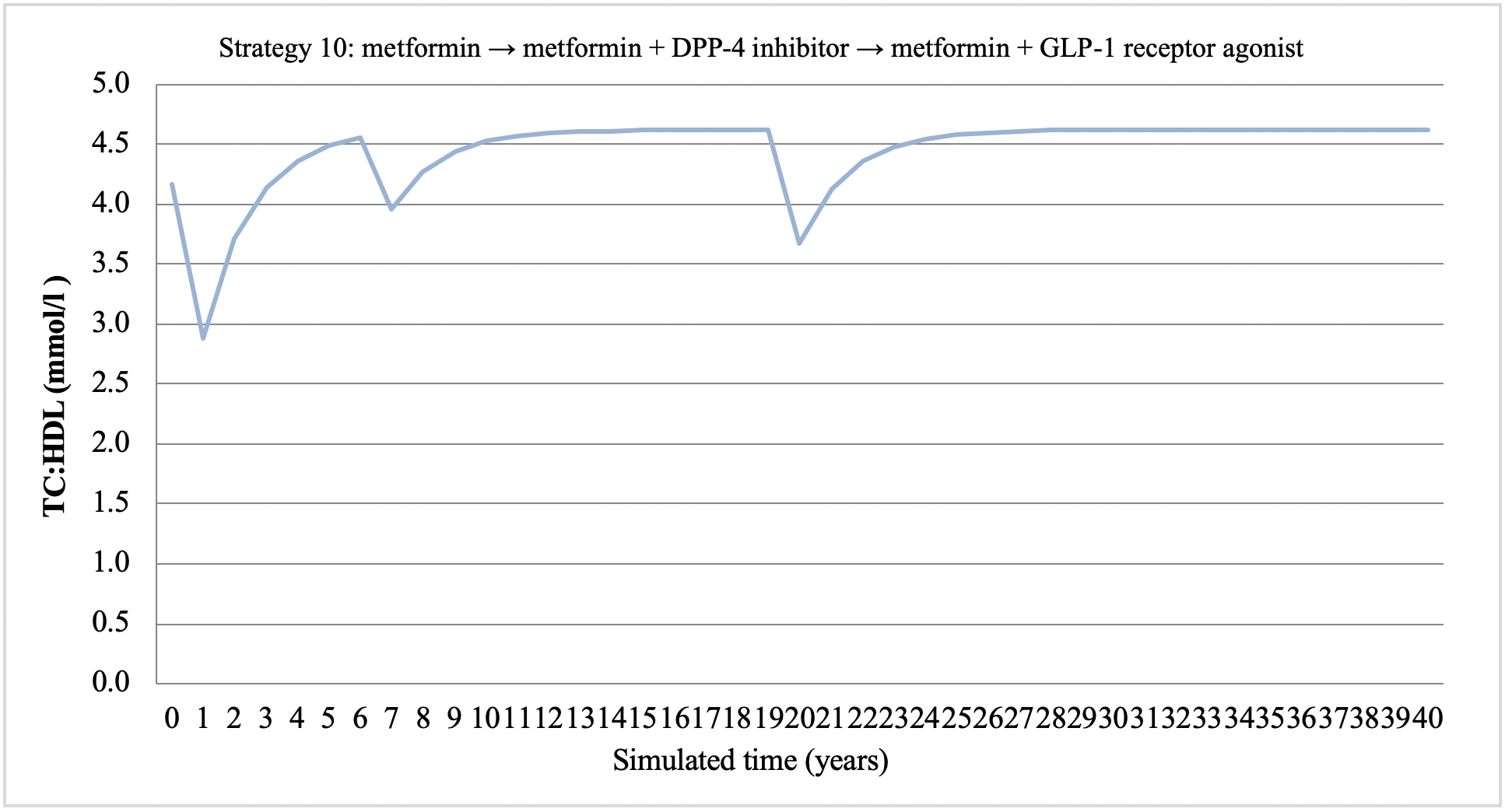


**Fig. S4. The trajectories of cholesterol in ten pharmacologic combination strategies over time: base-case analysis.** DPP-4, dipeptidyl peptidase 4. GLP-1, glucagon-like peptide 1.


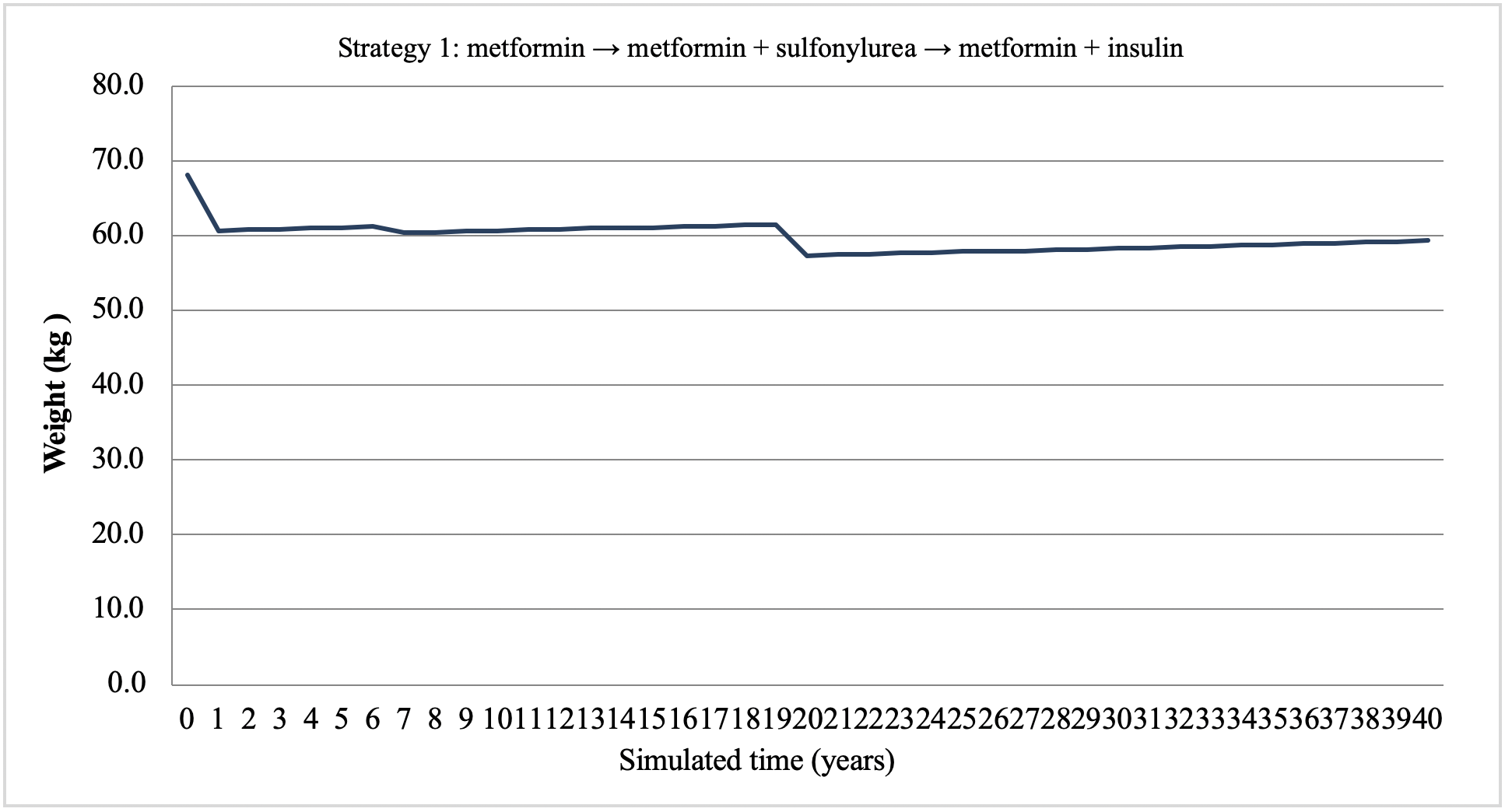

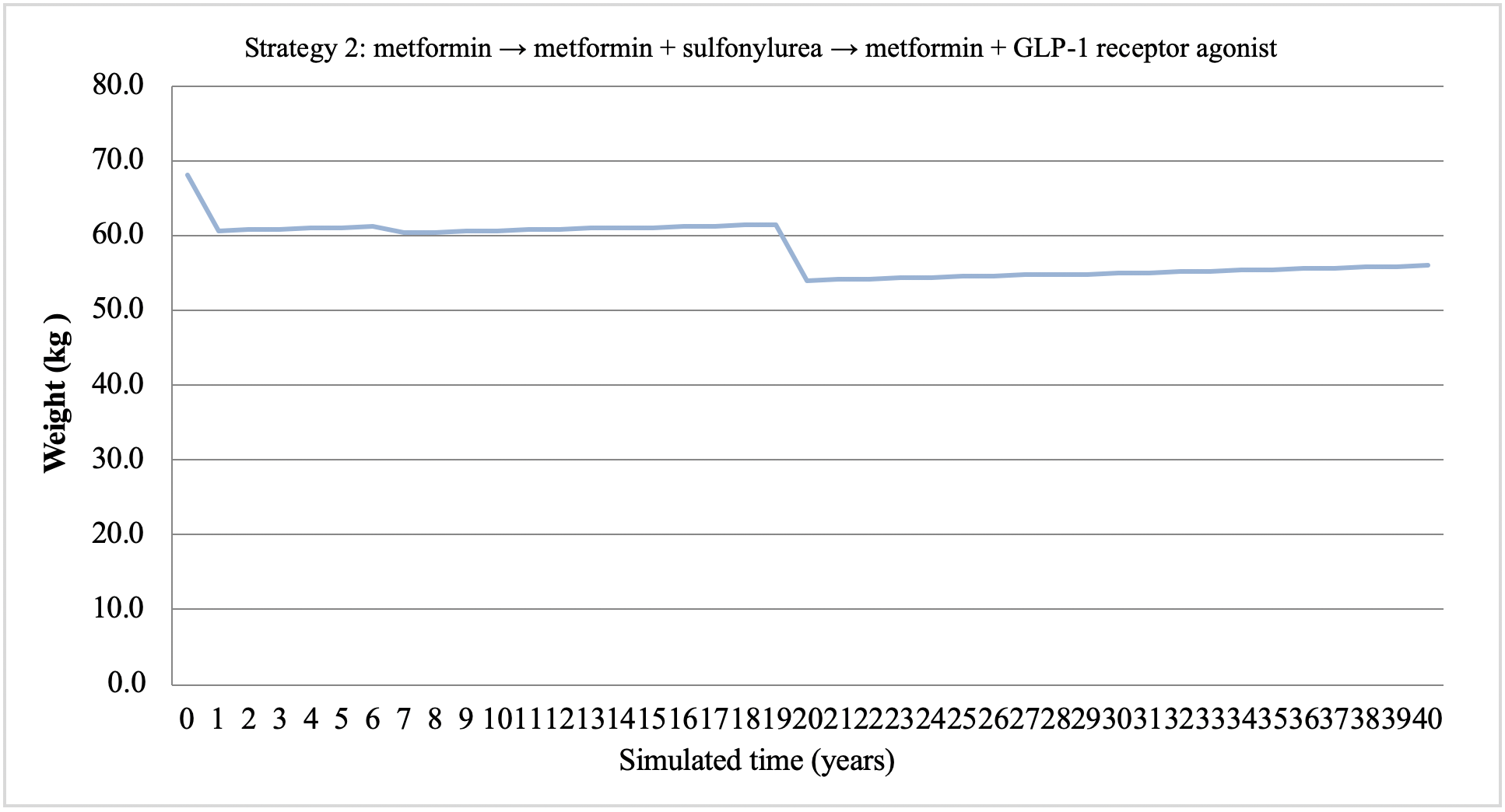


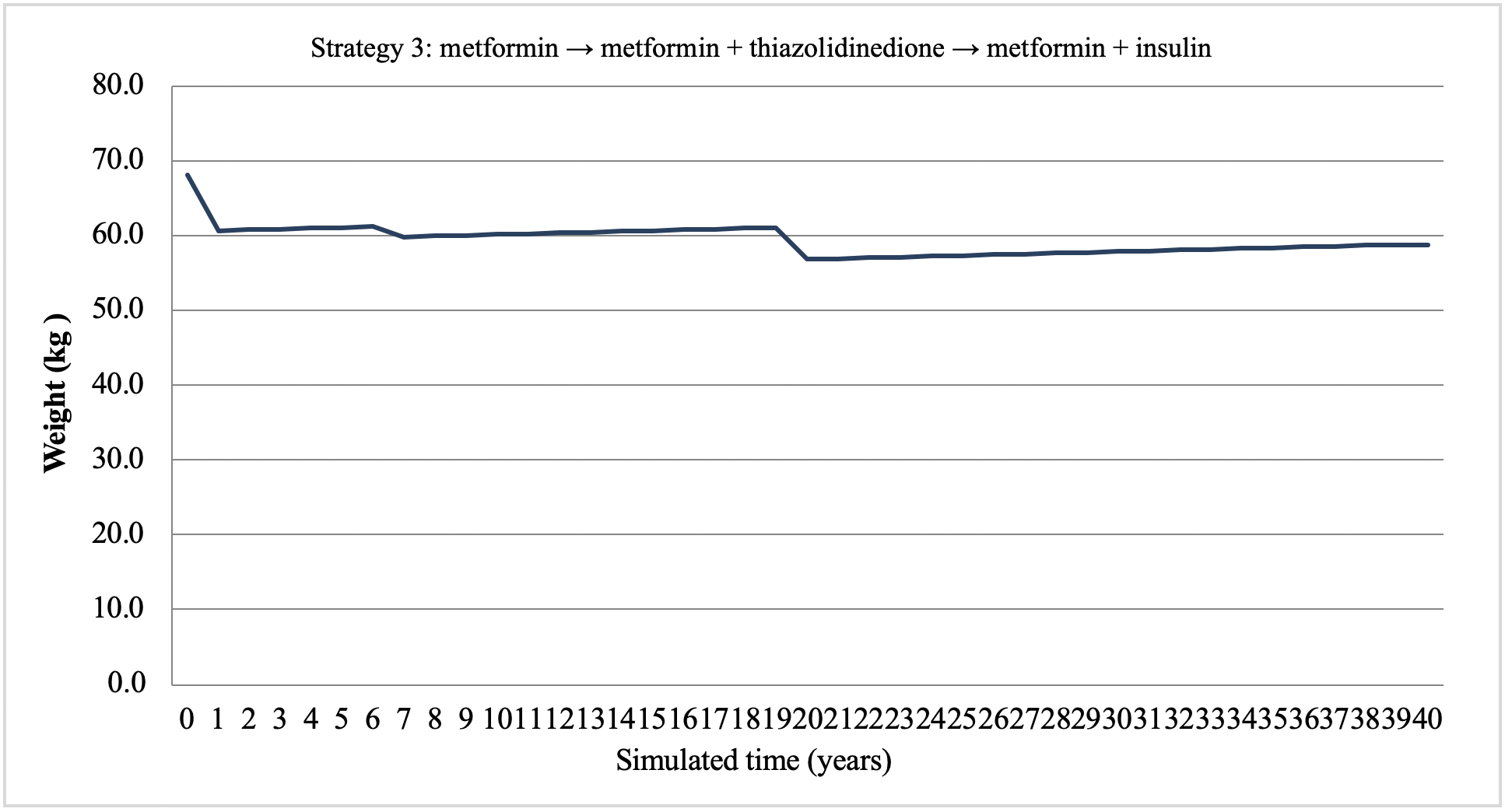

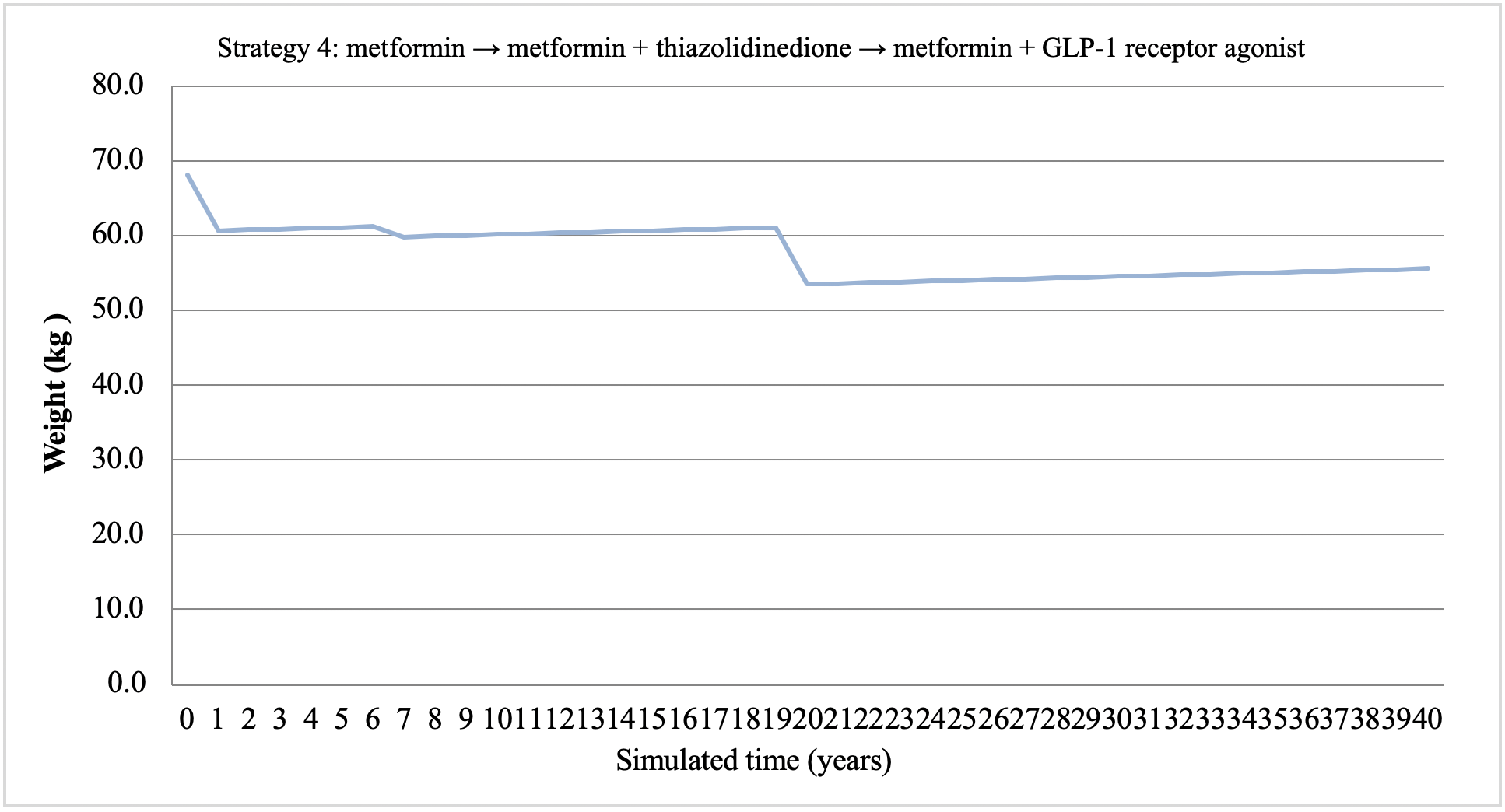


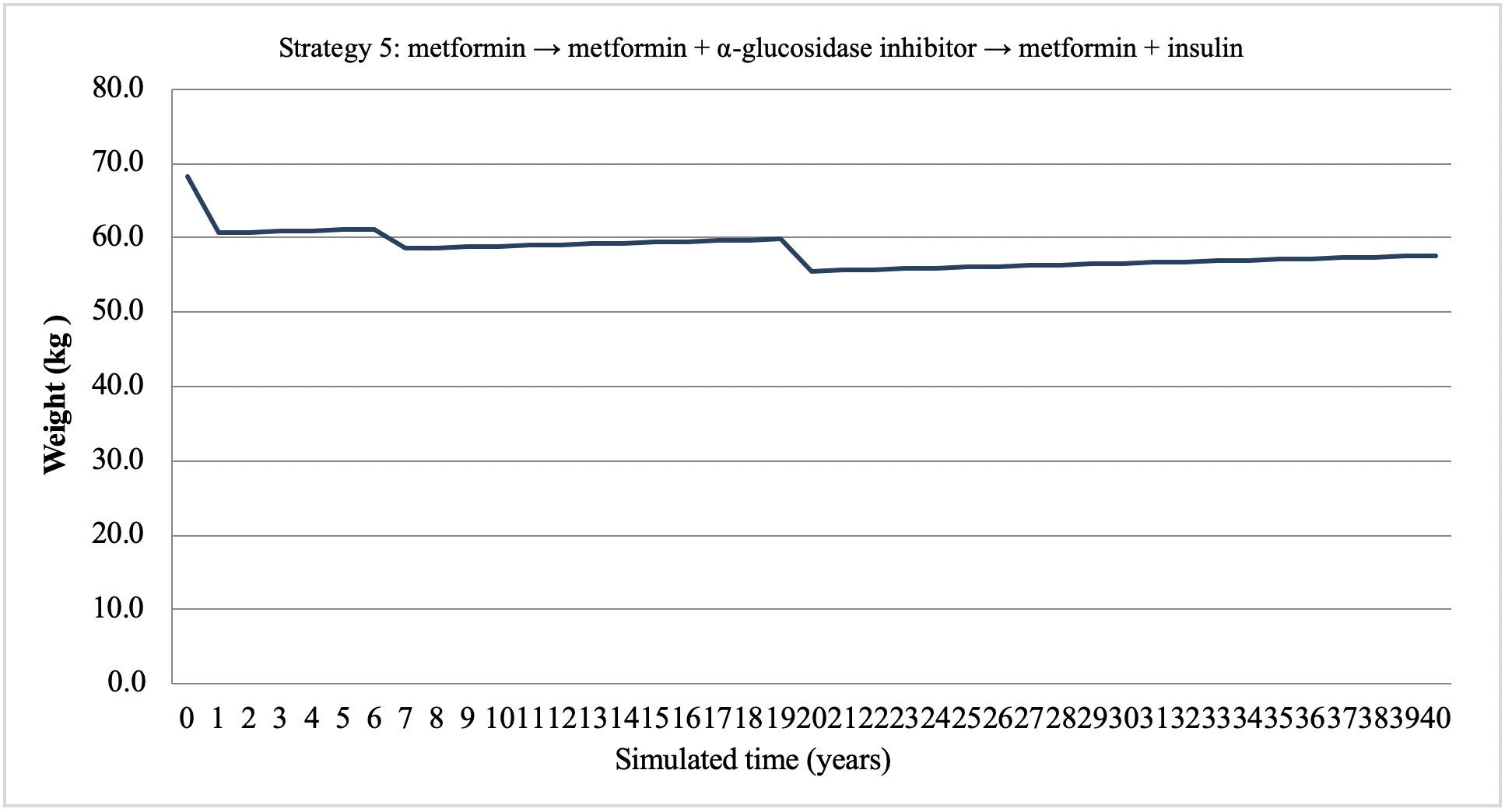

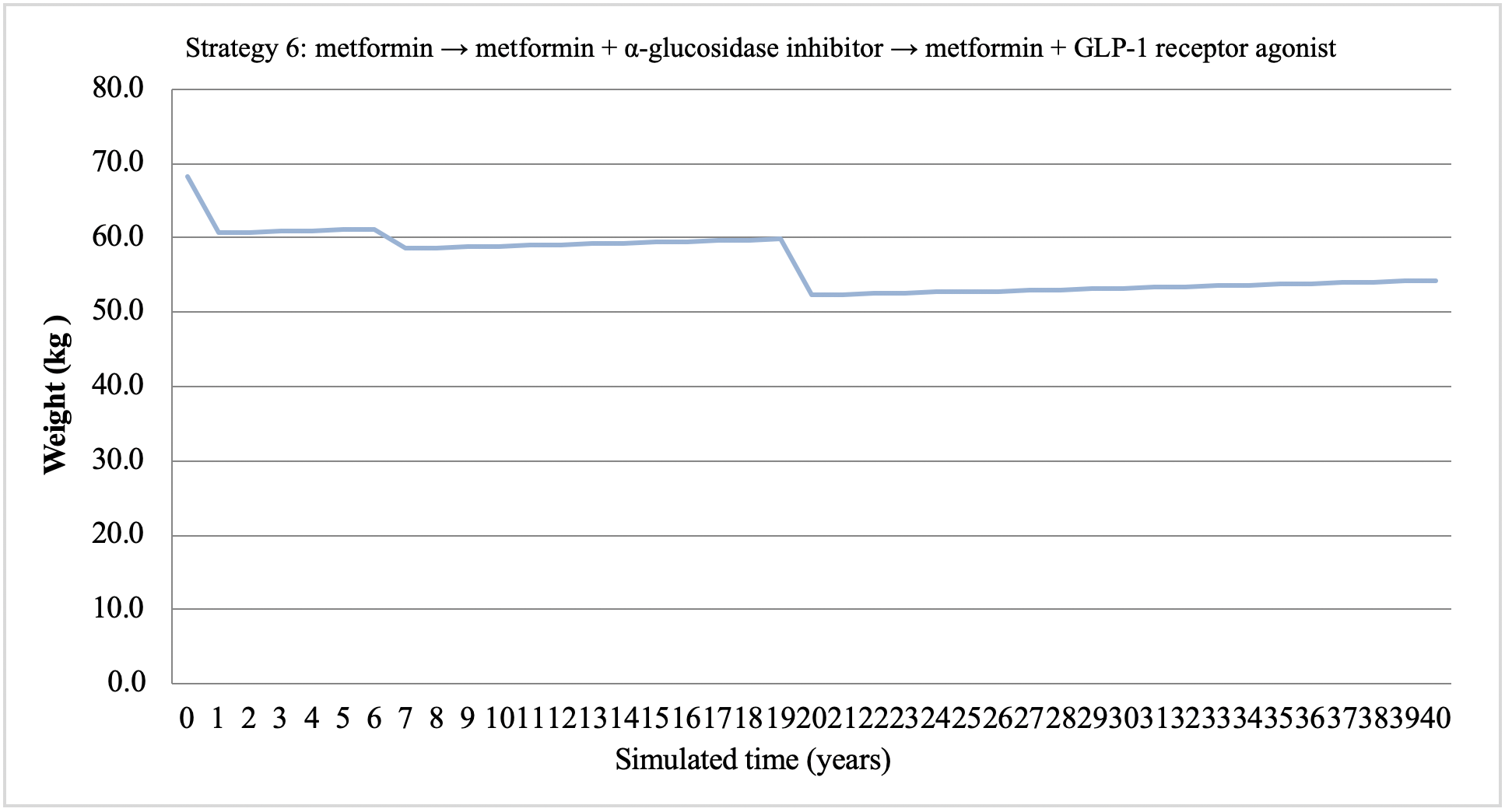


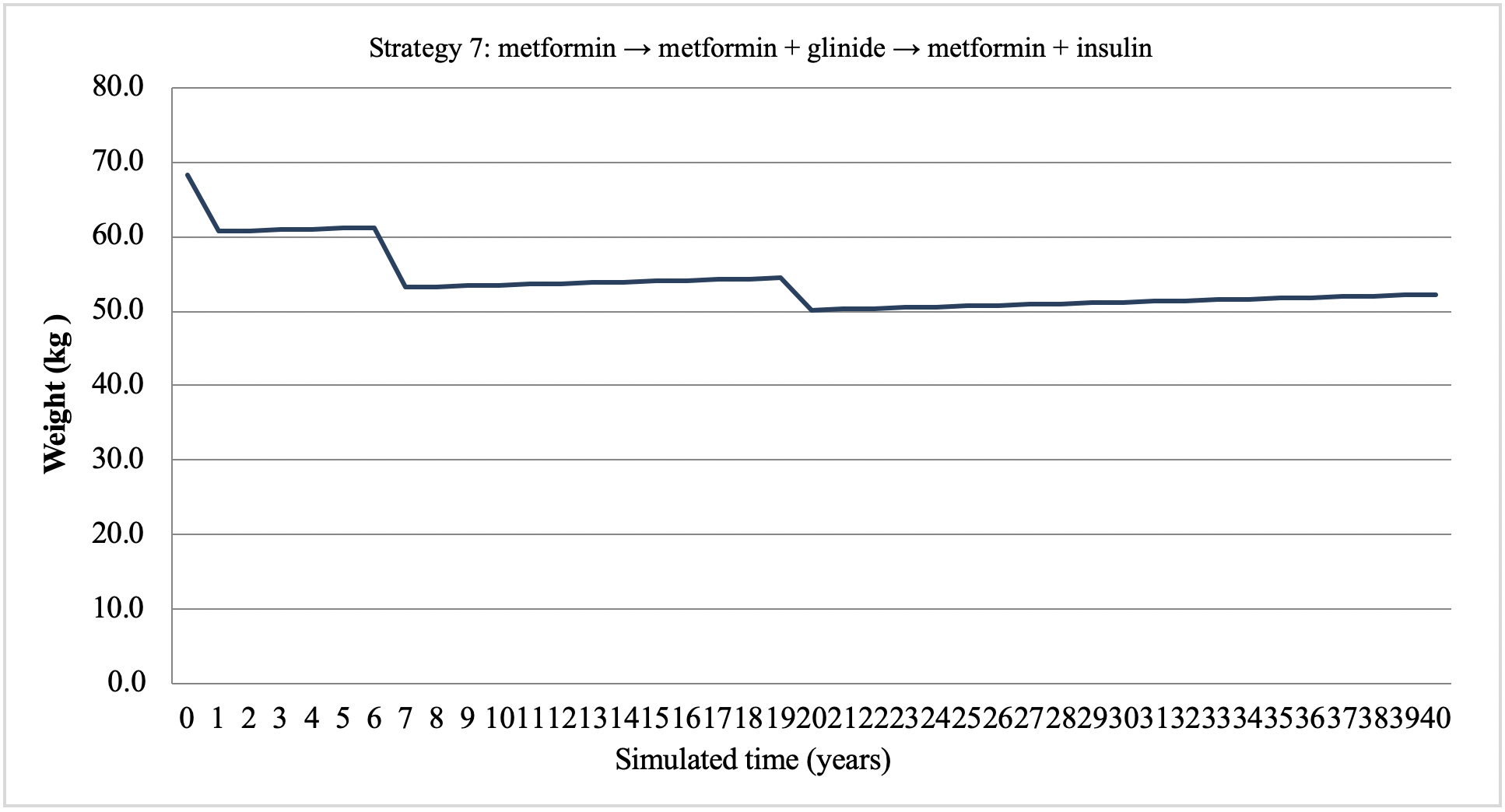

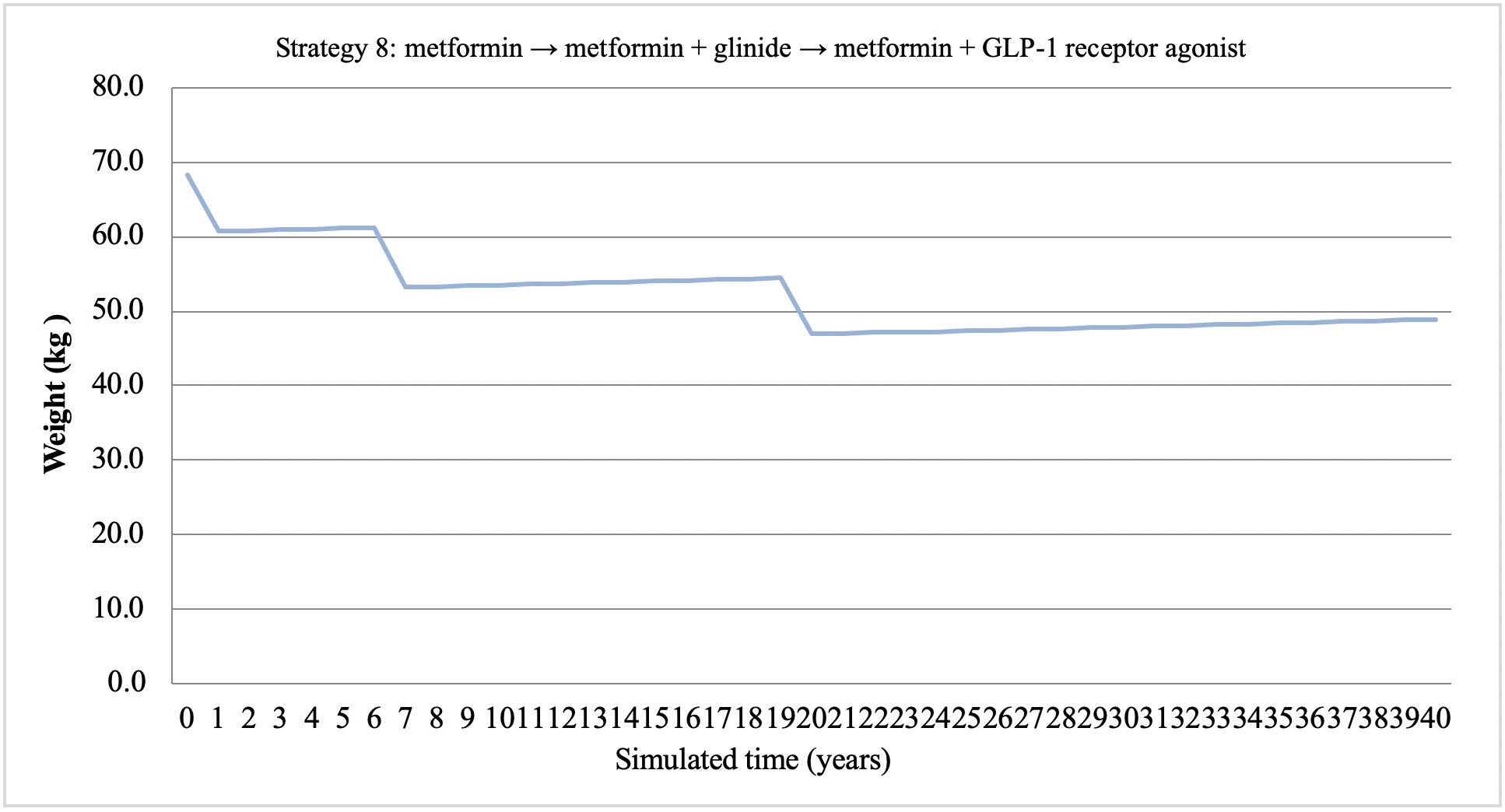


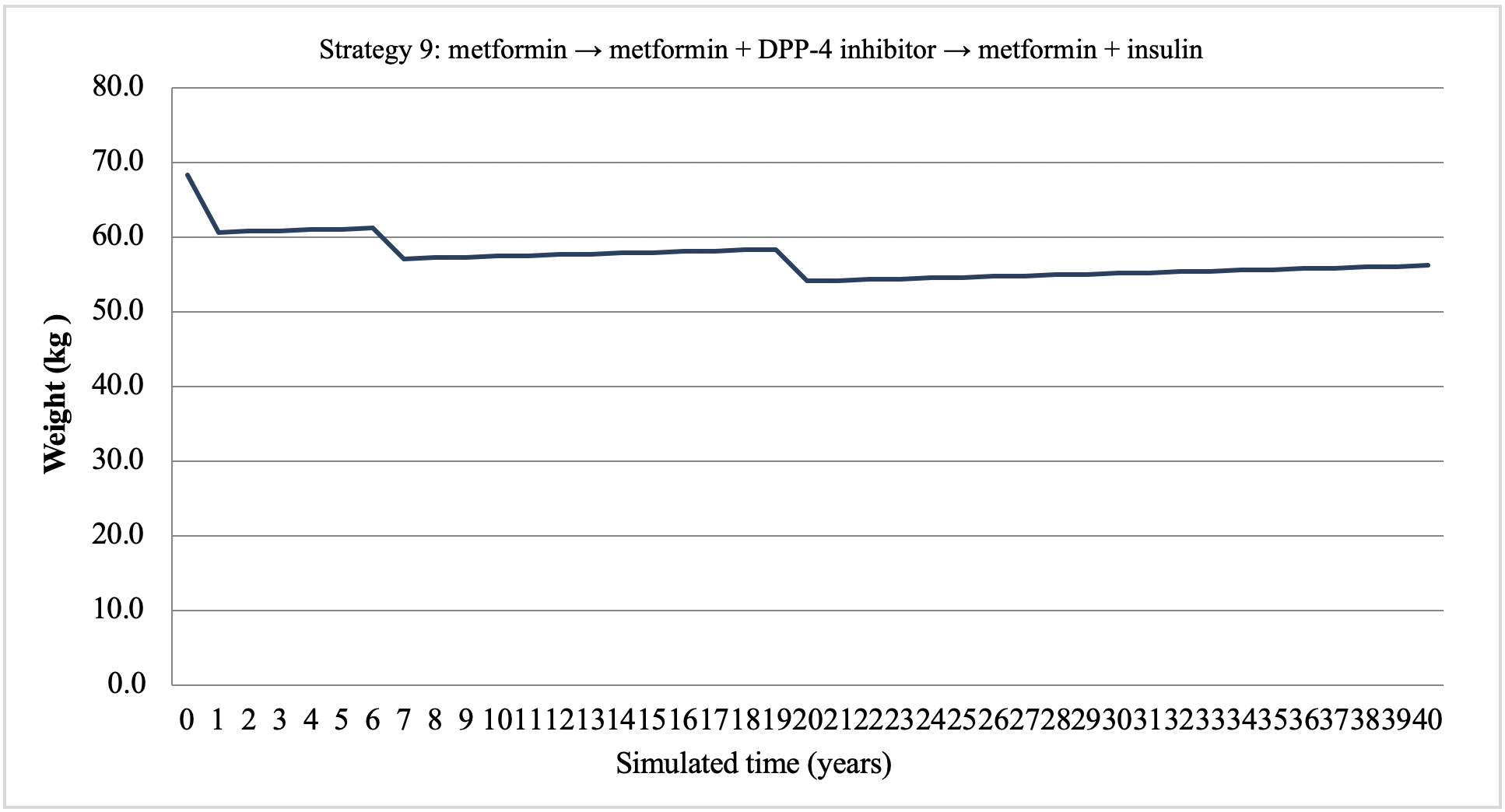

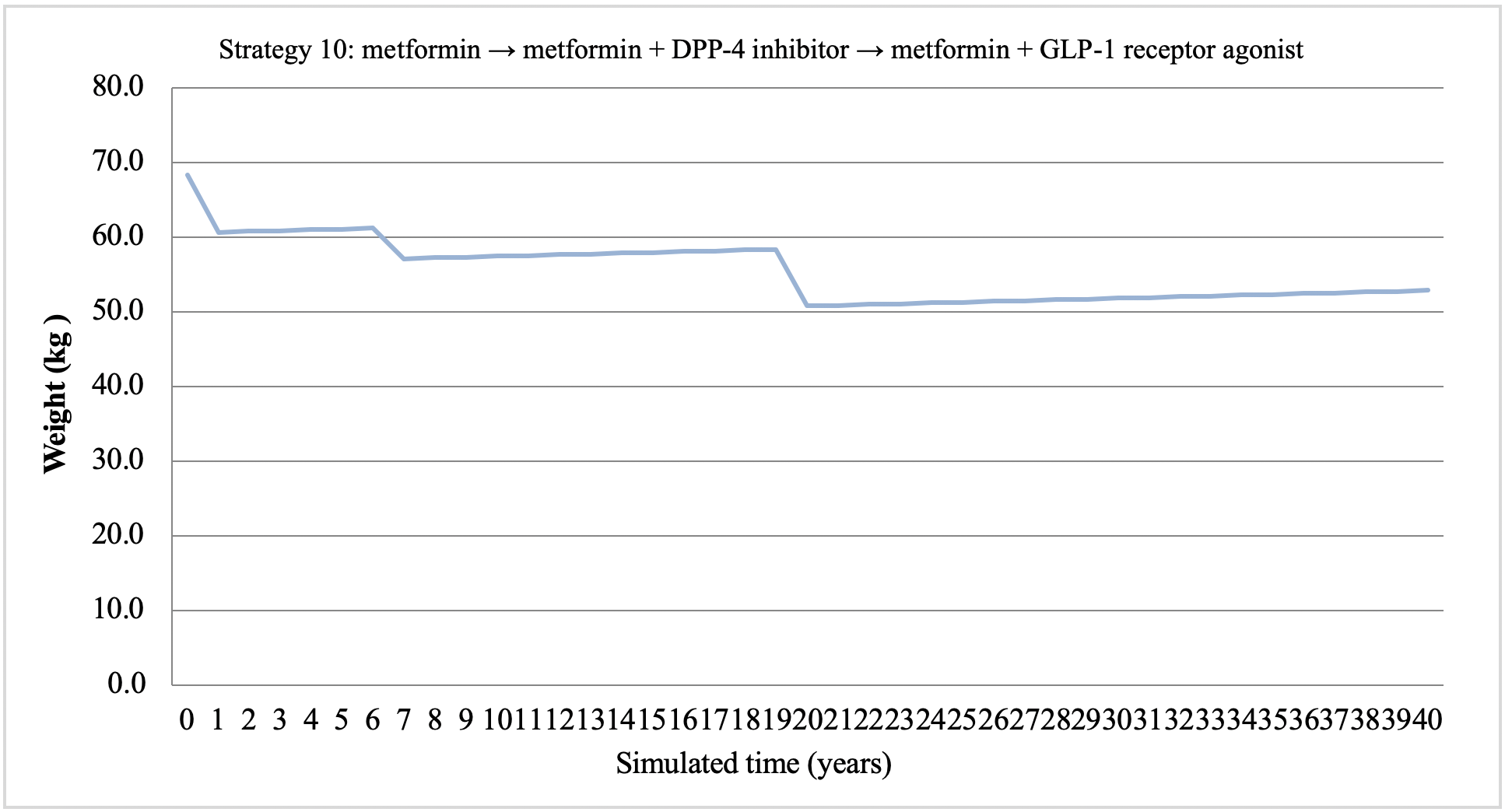


**Fig. S5. The trajectories of weight in ten pharmacologic combination strategies over time: base-case analysis.** DPP-4, dipeptidyl peptidase 4. GLP-1, glucagon-like peptide 1.


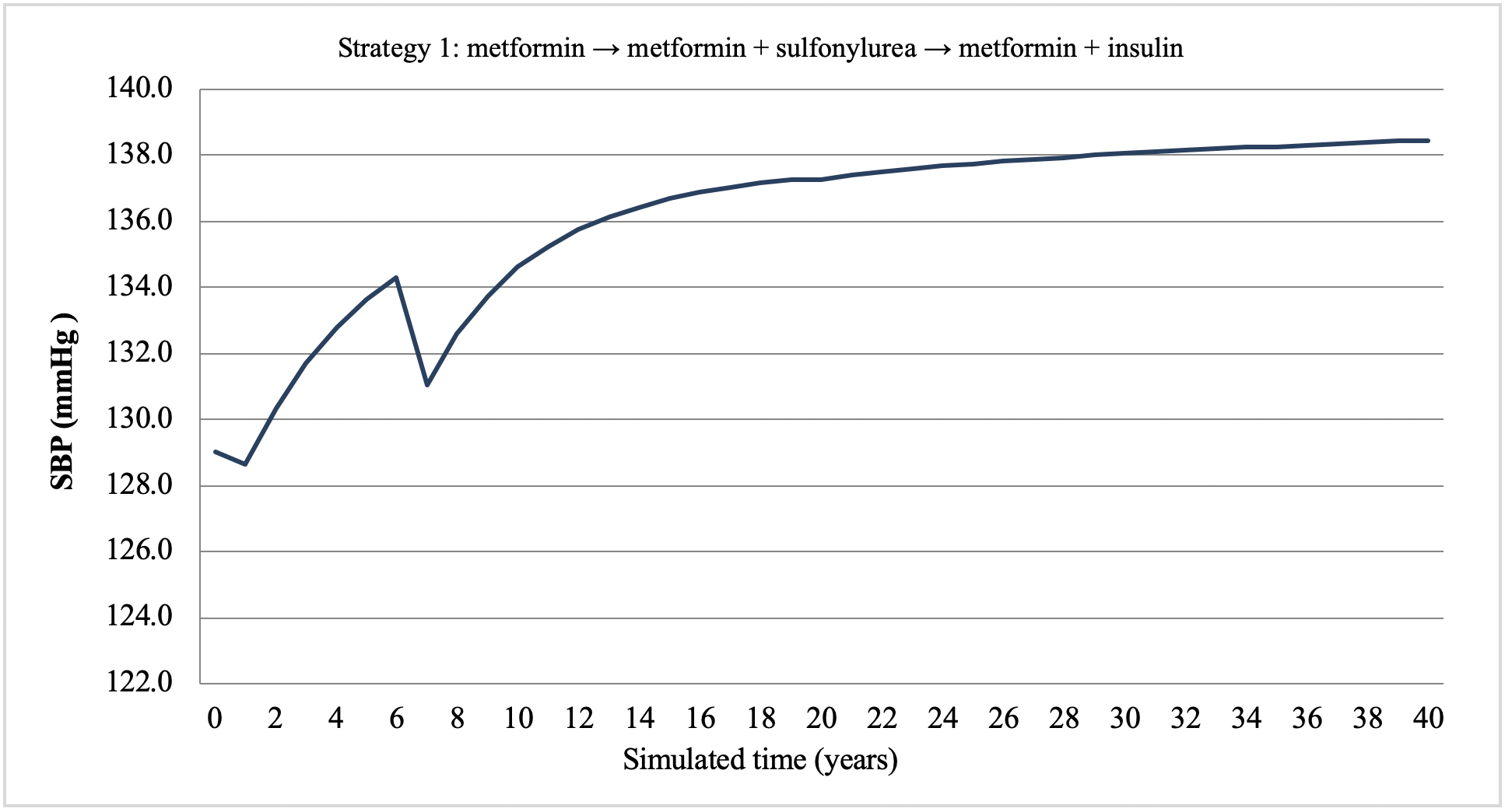

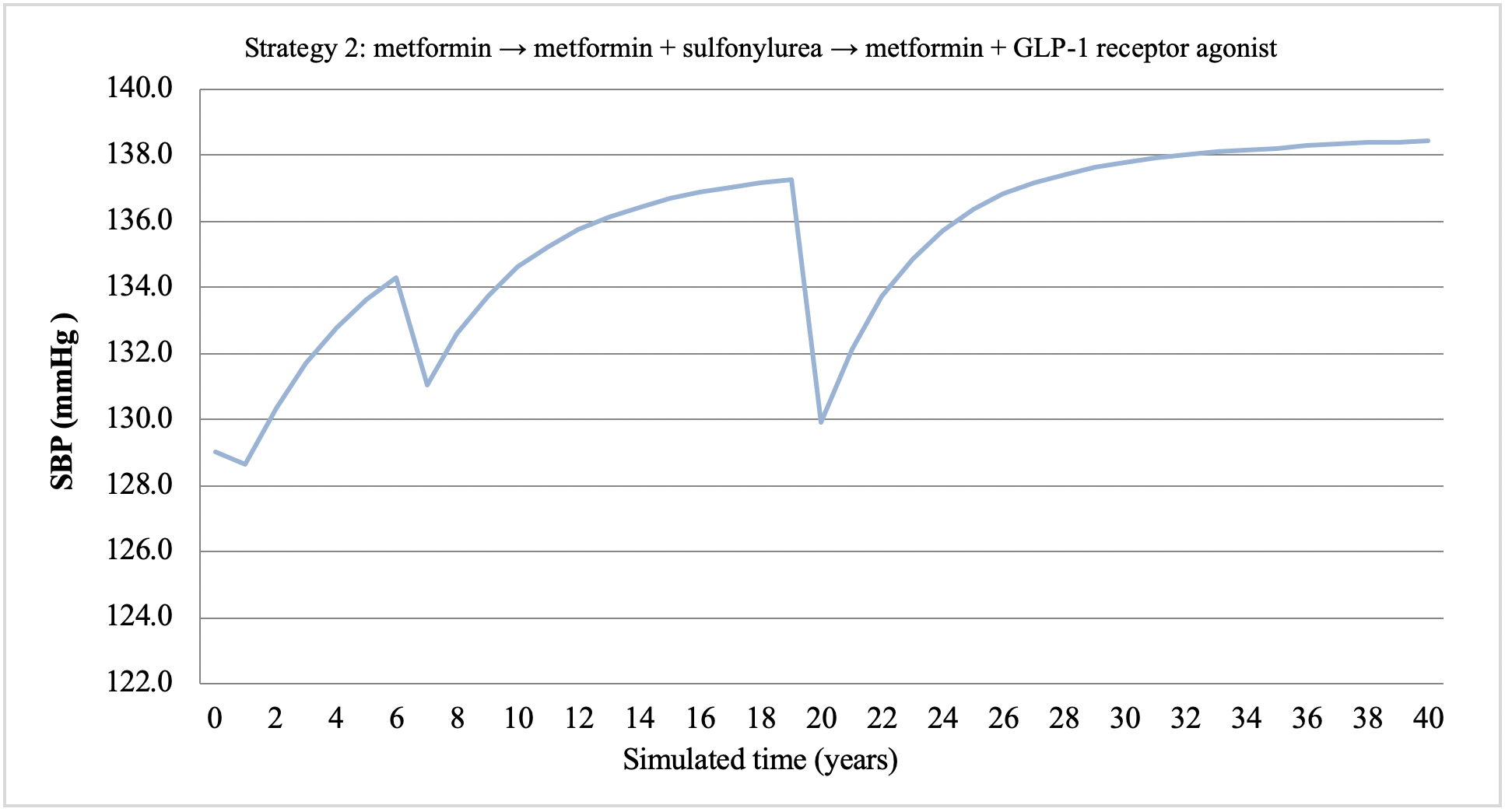


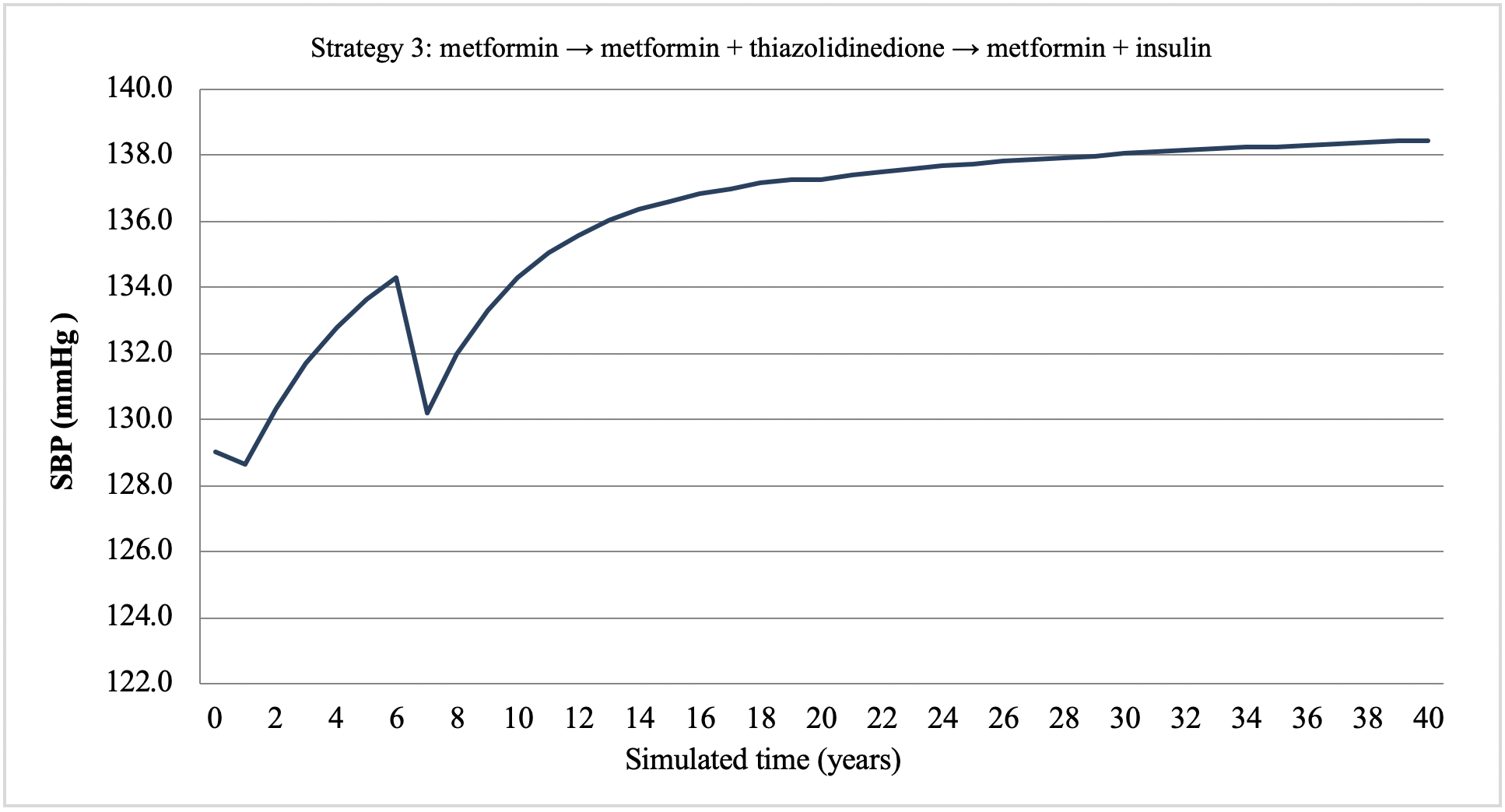

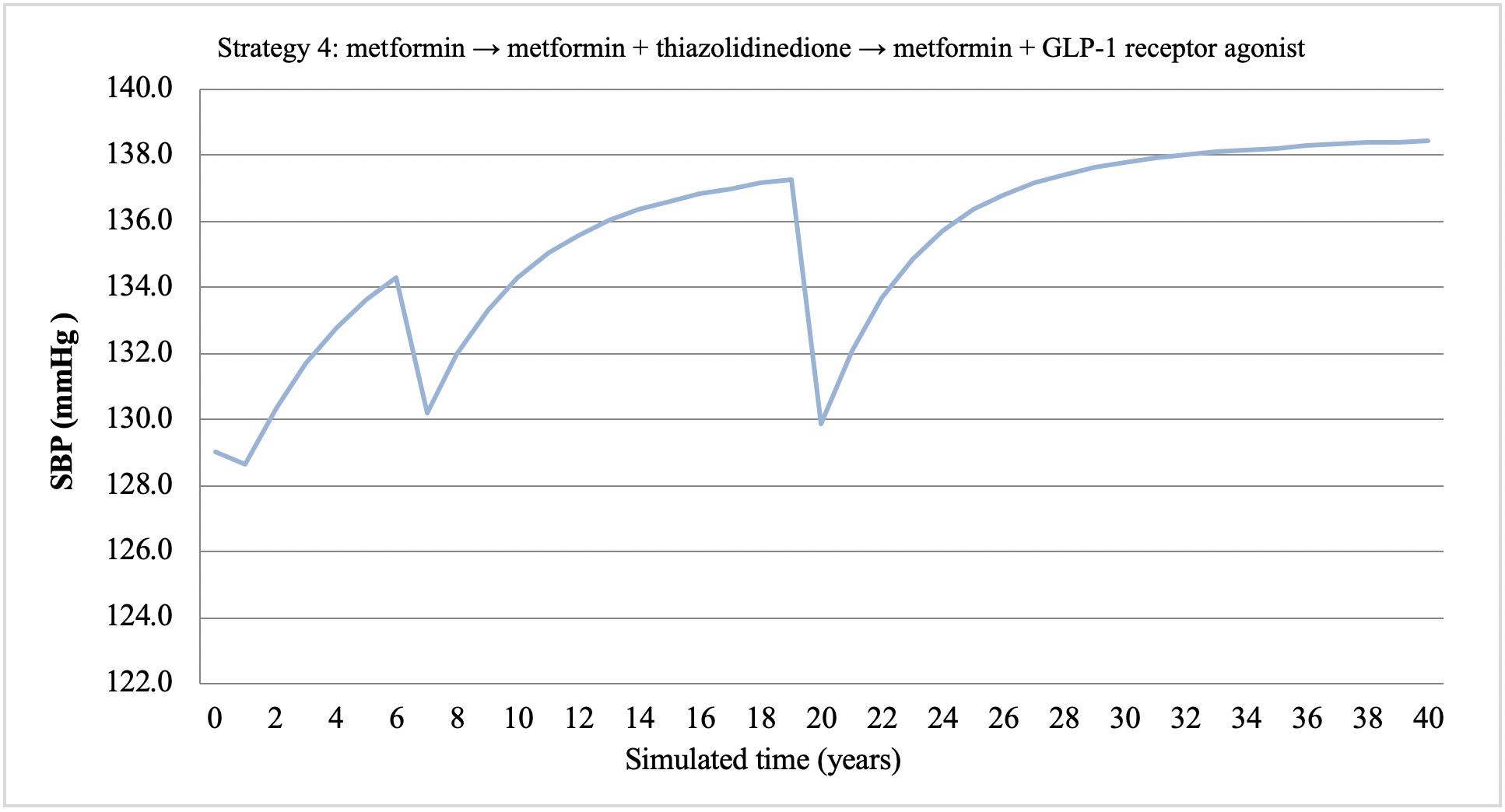


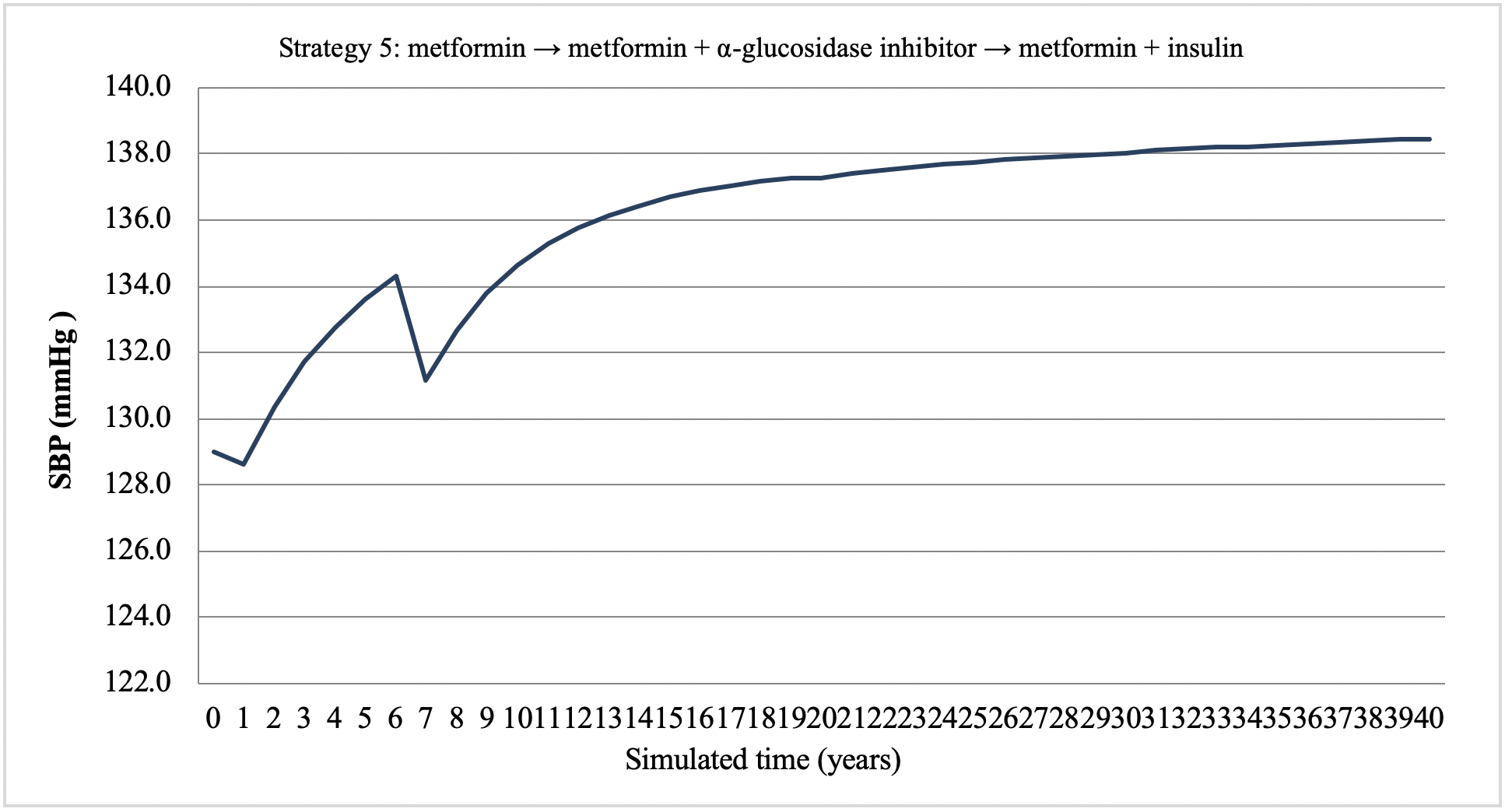

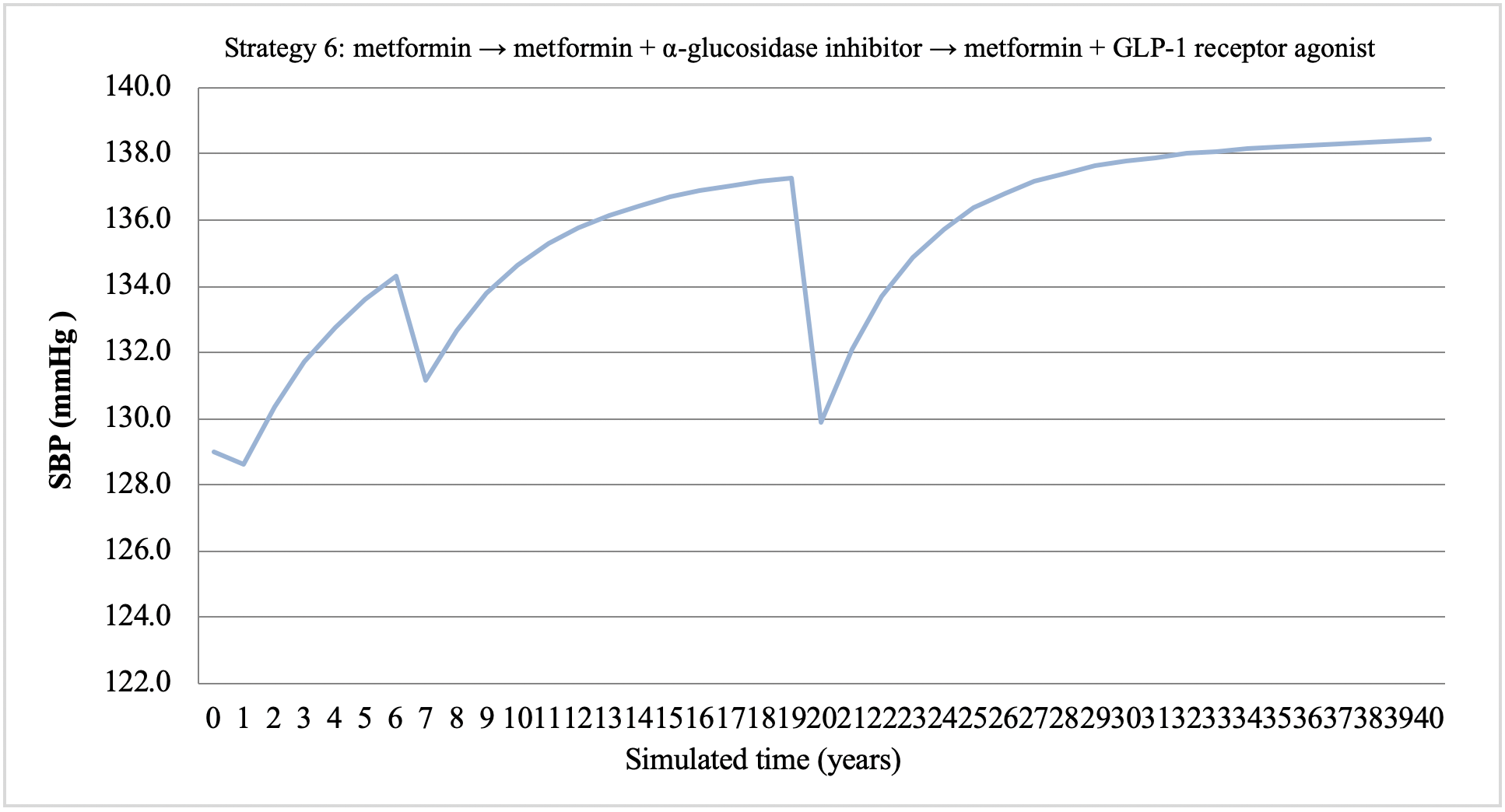


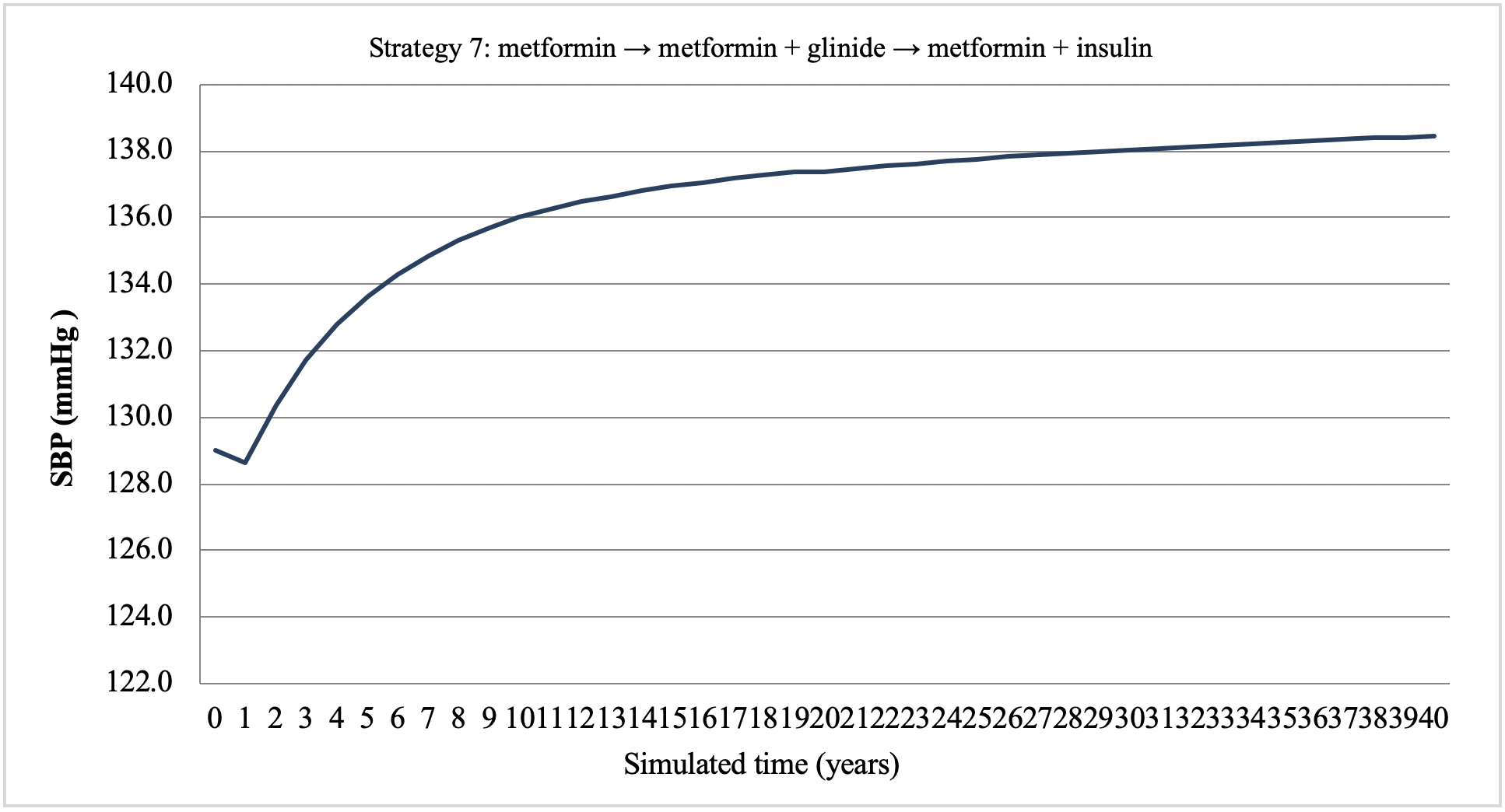

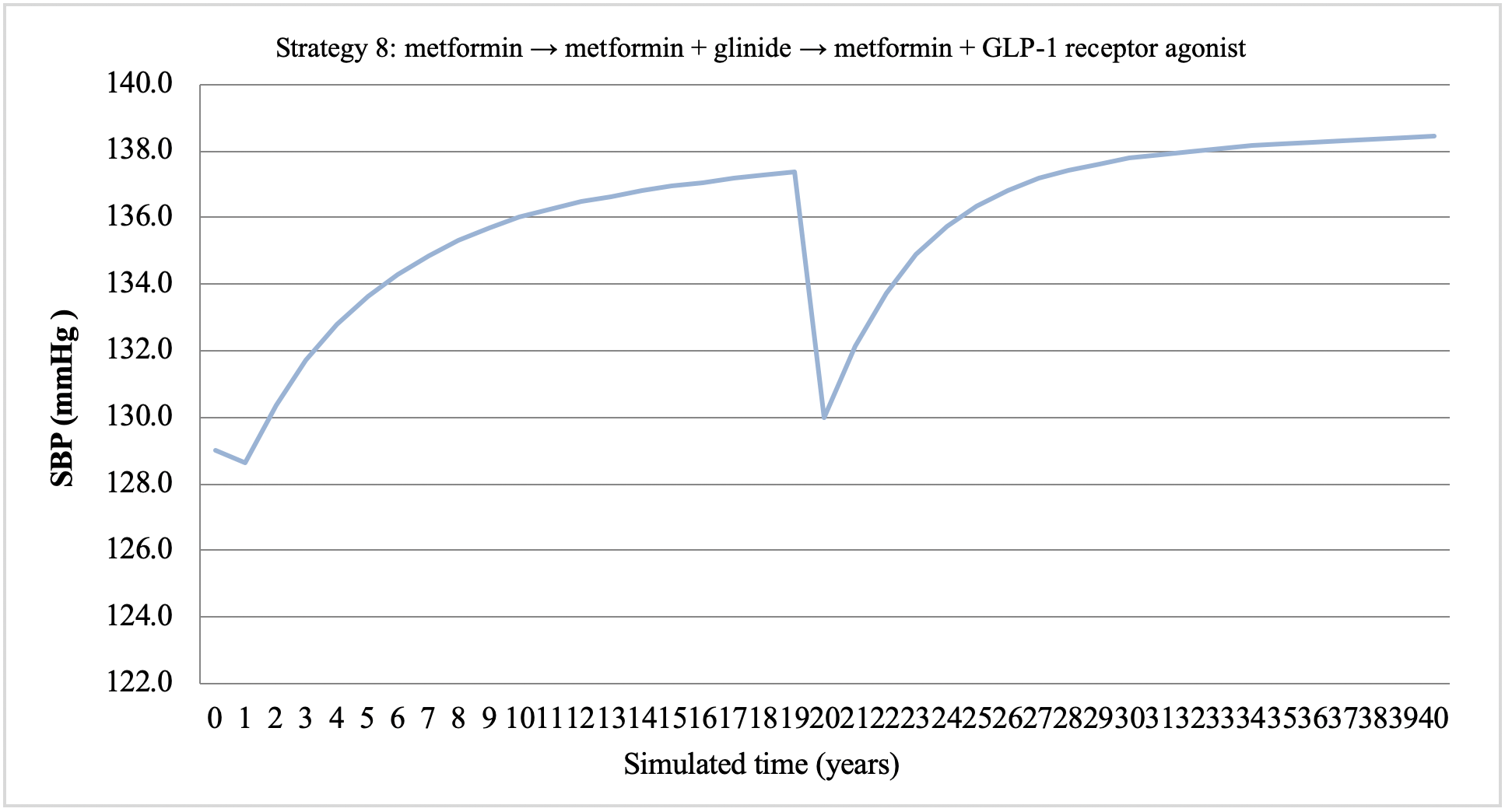


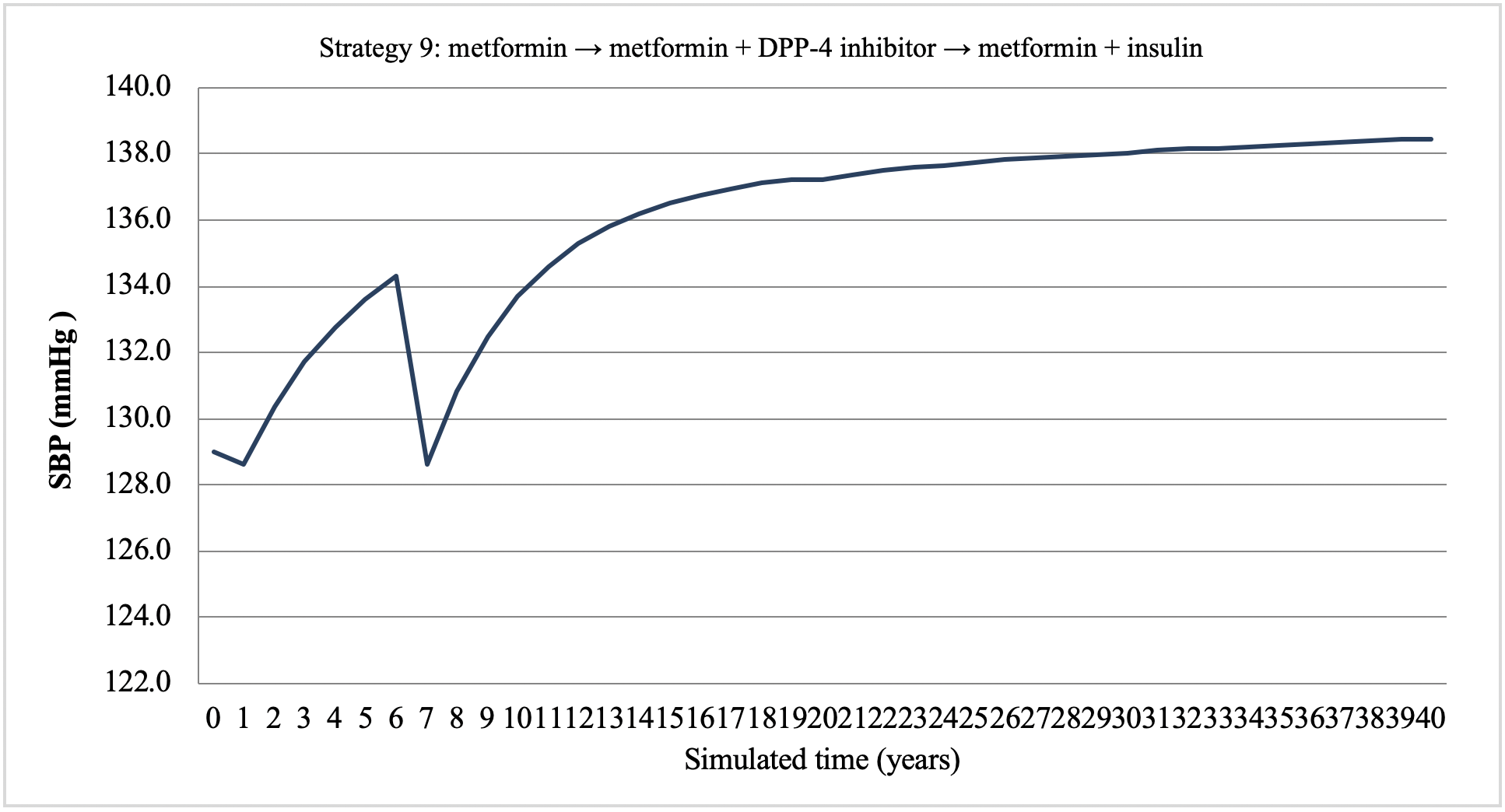

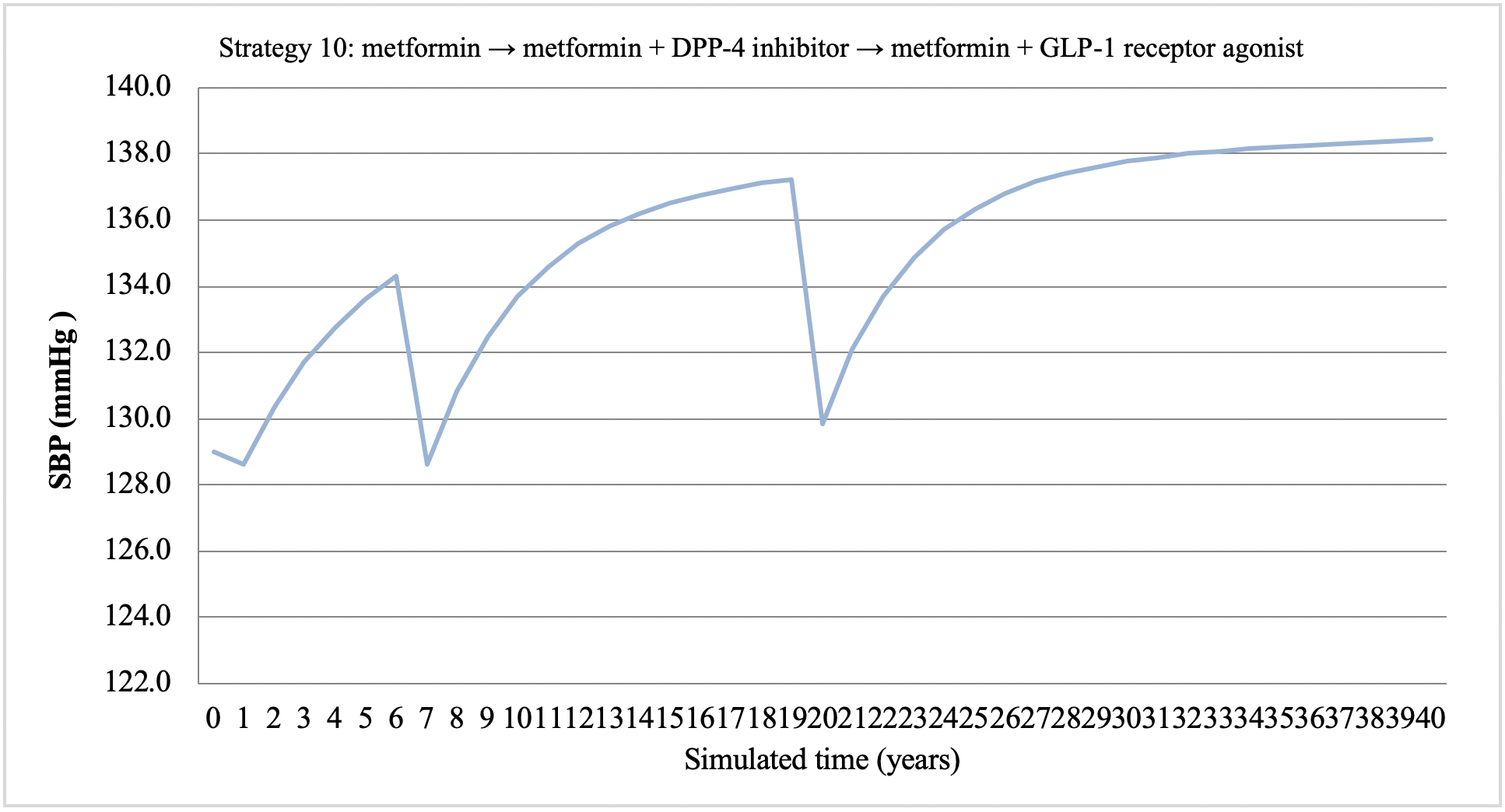


**Fig. S6. The trajectories of SBP in ten pharmacologic combination strategies over time: base-case analysis.** DPP-4, dipeptidyl peptidase 4. GLP-1, glucagon-like peptide 1. SBP, systolic blood pressure.
